# NETs decorated with bioactive IL-33 infiltrate inflamed tissues and induce IFN-α production in patients with SLE

**DOI:** 10.1172/jci.insight.147671

**Published:** 2021-11-08

**Authors:** Spiros Georgakis, Katerina Gkirtzimanaki, Garyfalia Papadaki, Hariklia Gakiopoulou, Elias Drakos, Maija-Leena Eloranta, Manousos Makridakis, Georgia Kontostathi, Jerome Zoidakis, Eirini Baira, Lars Rönnblom, Dimitrios T. Boumpas, Prodromos Sidiropoulos, Panayotis Verginis, George Bertsias

**Affiliations:** 1 Laboratory of Rheumatology, Autoimmunity and Inflammation, University of Crete, Medical School, Iraklio, Greece.; 2 Infections and Immunity, Institute of Molecular Biology and Biotechnology, Foundation for Research and Technology — Hellas (FORTH), Iraklio, Greece.; 3 1st Department of Pathology, National and Kapodistrian University of Athens Medical School, Athens, Greece.; 4 Department of Pathology, University of Crete, Medical School, Iraklio, Greece.; 5 Department of Medical Sciences, Rheumatology and Science for Life Laboratory, Uppsala University, Uppsala, Sweden.; 6 Biotechnology Division, Biomedical Research Foundation of the Academy of Athens, Athens, Greece.; 7 Laboratory of Toxicological Assessment of Pesticides, Scientific Directorate of Pesticides Assessment and Phytopharmacy, Benaki Phytopathological Institute, Athens, Greece.; 8 Center of Clinical, Experimental Surgery & Translational Research, Biomedical Research Foundation Academy of Athens, Athens, Greece.; 9 Joint Rheumatology Program and 4th Department of Internal Medicine, Attikon University Hospital, National and Kapodistrian University of Athens Medical School, Athens, Greece.; 10 Laboratory of Immune Regulation and Tolerance, University of Crete, Medical School, Iraklio, Greece.

**Keywords:** Autoimmunity, Inflammation, Cytokines, Lupus, Neutrophils

## Abstract

IL-33, a nuclear alarmin released during cell death, exerts context-specific effects on adaptive and innate immune cells, eliciting potent inflammatory responses. We screened blood, skin, and kidney tissues from patients with systemic lupus erythematosus (SLE), a systemic autoimmune disease driven by unabated type I IFN production, and found increased amounts of extracellular IL-33 complexed with neutrophil extracellular traps (NETs), correlating with severe, active disease. Using a combination of molecular, imaging, and proteomic approaches, we show that SLE neutrophils, activated by disease immunocomplexes, release IL-33–decorated NETs that stimulate robust IFN-α synthesis by plasmacytoid DCs in a manner dependent on the IL-33 receptor ST2L. IL33-silenced neutrophil-like cells cultured under lupus-inducing conditions generated NETs with diminished interferogenic effect. Importantly, NETs derived from patients with SLE are enriched in mature bioactive isoforms of IL-33 processed by the neutrophil proteases elastase and cathepsin G. Pharmacological inhibition of these proteases neutralized IL-33–dependent IFN-α production elicited by NETs. We believe these data demonstrate a novel role for cleaved IL-33 alarmin decorating NETs in human SLE, linking neutrophil activation, type I IFN production, and end-organ inflammation, with skin pathology mirroring that observed in the kidneys.

## Graphical abstract

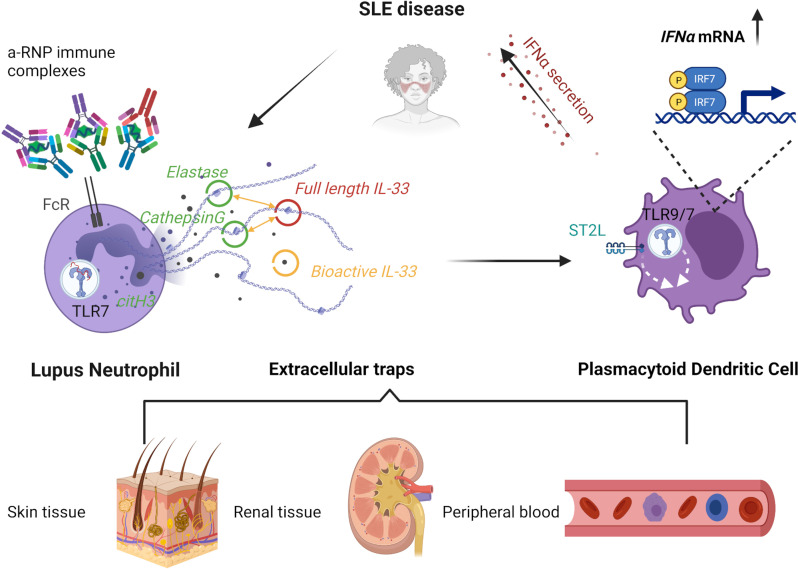

## Introduction

Systemic lupus erythematosus (SLE) is the prototype systemic autoimmune disease characterized by dysregulated innate and adaptive immunity leading to generation of autoantibodies and formation of immune complexes (ICs) deposited to afflicted tissues such as the skin and kidneys (1). Transcriptome studies (2, 3) have implicated neutrophils in SLE, which is further corroborated by genetic (4, 5) and functional (6) evidence. A cardinal disease feature is unabated production of type I IFN (2), which exerts pleiotropic effects on hematopoietic and nonhematopoietic cells, thus propagating inflammation (7, 8). One of the primary sources of IFN is plasmacytoid DCs (pDCs) especially in response to TLR-7 and -9 signaling (8). Genetic depletion (9) or inactivation (10) of pDCs diminishes IFN and ameliorates lupus-like disease in animal models, and likewise, therapeutic targeting of pDCs and/or type I IFN has yielded promising results in human SLE (11, 12). Various molecules can instruct IFN production by pDCs, such as neutrophil extracellular traps (NETs) complexed with autoantibodies and immunostimulatory proteins (13–16); however, the mechanisms that perpetuate IFN in SLE are incompletely understood.

IL-33 is a chromatin-bound alarmin that exists in the nucleus as full-length isoform (31 kDa), whereas it is externalized mainly during necrotic cell death of nonhematopoietic cells as a shorter (18 kDa) cytokine-like isoform that signals through the ST2L receptor (17). Immune cells express and release IL-33 upon stimulation by death- or damage-related agents. Notably, full-length IL-33 ([fl]IL-33) has modest bioactivity extracellularly, which, nevertheless, is profoundly induced after cleavage by neutrophil and mast cell serine proteases or allergen-related cysteine proteases (18).

Previous work (19, 20) has suggested context-specific expression and function of IL-33. Although IL-33 supports regulatory T cell (21) and nonpathogenic Th2 responses (22), it can also drive T cells to become effectors (23). Moreover, IL-33 may confer autoimmunity by augmenting innate immune responses (24). In SLE, elevated serum concentrations of IL-33 and the soluble form of the IL-33 receptor ST2L have been reported (25–27). Genetic studies have also implicated *IL33* gene polymorphisms in susceptibility to SLE (28, 29). In animal studies, exogenous IL-33 induced the B cell activating factor (30), a growth factor implicated in SLE, and MRL/*lpr* lupus–prone mice treated with anti–IL-33 antibody have reduced renal inflammation and serum autoantibodies (31). However, the in vivo expression and function of IL-33, as well as its contribution to immune cell activation and tissue inflammation in SLE, remain ill defined.

Herein, driven by our striking finding of extracellular IL-33 complexed with NETs in lupus-afflicted tissues, we reasoned that NETting neutrophils may represent an important source of IL-33 alarmin contributing to excessive type I IFN. In addition to skin and kidneys, screening of serum samples from patients with active SLE revealed IL-33–complexed NETs, thus reinforcing the disease pathogenesis and end-organ injury roles of NETs. Through genetic, imaging, and proteomic assays, we confirmed that ex vivo, cultured, human SLE neutrophils and their NETs express significant amounts of IL-33. Our studies denote a critical role for IL-33 as a chromatin-bound alarmin mediating the IFN-inducing effect of SLE NETs through interaction with the ST2L receptor on pDCs. Importantly, we implicate neutrophil proteases for the production of cleaved interferogenic IL-33 isoforms on lupus NETs, thus suggesting that interference with IL-33 maturation and/or signaling could be therapeutically exploited.

## Results

### IL-33–decorated NETs were detected in inflamed tissues of patients with active SLE.

**Figure 1:**
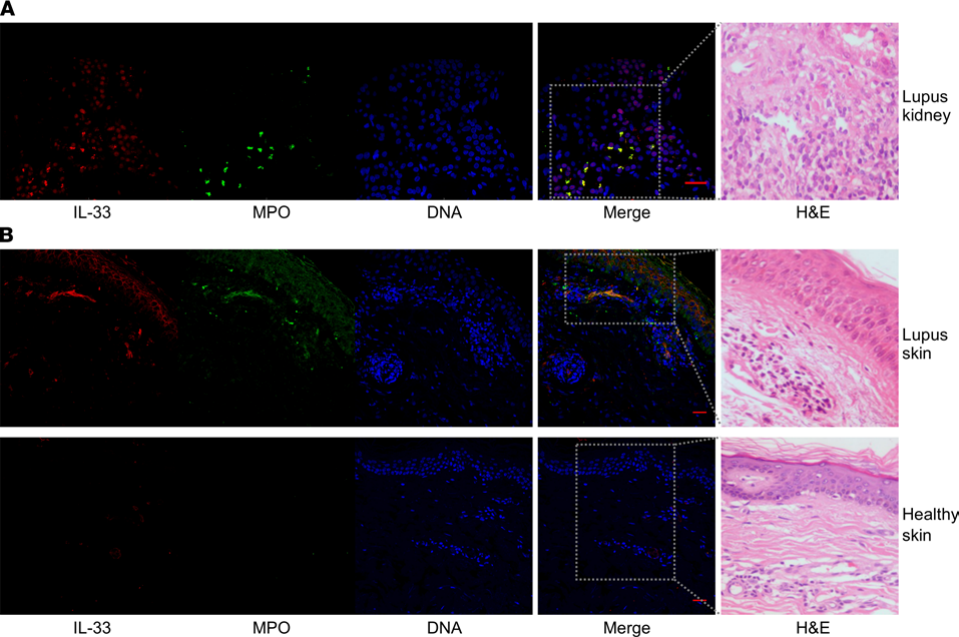
IL-33–decorated NETs are present in inflamed tissues of patients with active SLE. (**A** and **B**) IL-33–complexed NETs visualized by confocal microscopy on kidney and skin sections from patients with active proliferative lupus nephritis and cutaneous lupus, respectively. Skin sections from healthy donors were used as controls. IL-33 NETs are identified through immunostaining with anti-MPO and anti–IL-33 (IL-33) antibodies (green: MPO; red: IL-33; blue: DAPI/DNA). Representative confocal image (scale bar: 30 μm) of 4 patients. The same tissue sections were also stained with H&E (×400 magnification).

We focused on the role of IL-33, a nuclear alarmin released during cell death, in SLE. To gain initial insights, we monitored biopsy specimens from the kidney and skin, which represent 2 frequently affected organs in SLE, of patients with active disease (1). We identified extracellular structures in SLE kidneys, especially at the tubulointerstitium, where IL-33 and NET markers were colocalized, indicative of IL-33–containing NETs (Figure 1A). IL-33–decorated chromatin structures were also detected in lupus-affected dermis (corroborated by H&E staining), contrary to healthy skin, which exhibited no inflammation or IL-33 expression (Figure 1B).

**Figure 2:**
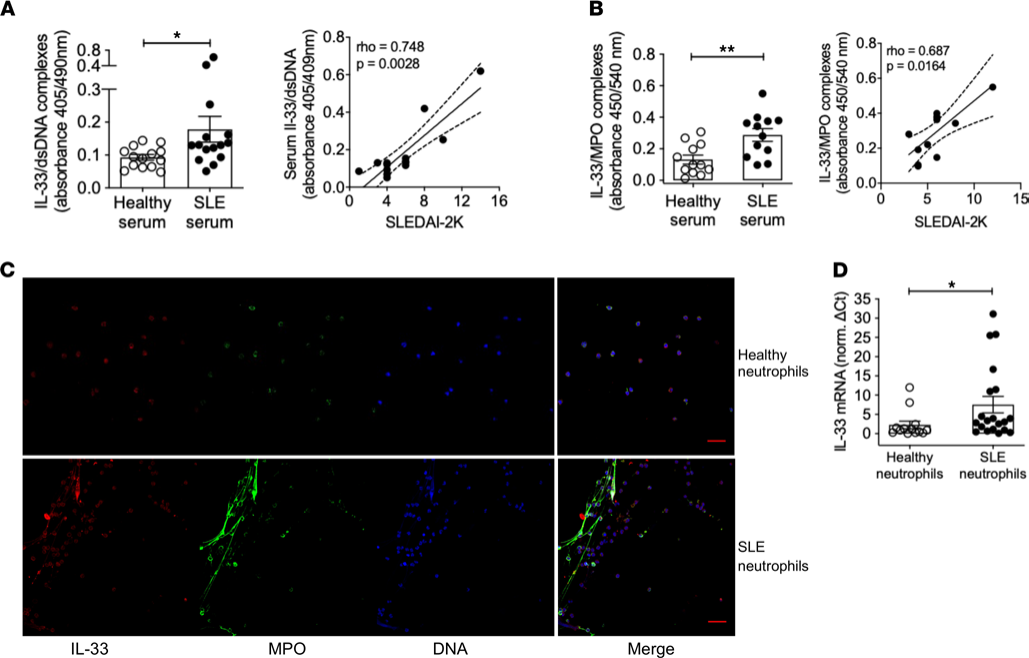
SLE neutrophils are prone to releasing IL-33–decorated NETs. (**A** and **B**) IL-33/dsDNA and IL-33/MPO complexes were quantified by sandwich ELISA in serum samples from healthy individuals (*n* = 14 and *n* = 12, respectively) and patients with SLE (*n* = 15 and *n* = 12, respectively). Each dot represents a different donor, and bar plots show the mean ± SEM absorbance (405/490 nm and 450/540 nm in **A** and **B**, respectively). *P < 0.05; **P < 0.01 (2-tailed, Mann-Whitney test). Serum IL-33/dsDNA and IL-33/MPO complexes were positively correlated with disease activity (assessed by the SLEDAI-2K) in patients with SLE (Spearman’s ρ = 0.748 and 0.687, respectively). Dashed lines demonstrate the 95% boundaries of the regression line. (**C**) Blood neutrophils were purified from healthy donors and patients with SLE, seeded onto coverslips coated with poly-l-lysine and cultured for 3 hours, followed by staining with anti-MPO and anti–IL-33 antibodies. DAPI was used for DNA staining. Representative confocal images of 3 experiments (scale bar: 30 μm) are shown. (**D**) Real-time PCR was performed to assay differential expression of *IL33* mRNA in freshly isolated blood neutrophils from healthy individuals (*n* = 13) versus patients with SLE (*n* = 20). Data were normalized using the average of healthy donors ΔCt (i.e., IL-33 Ct minus GAPDH Ct) values. Each dot represents a different donor, and bar plots show the mean ± SEM expression. *P < 0.05 (2-tailed; Mann-Whitney test).

Based on previous work linking blood neutrophil activation and NETosis in active severe SLE (2, 32–34), we screened for IL-33 NETs in the serum of patients with SLE. For this, we developed 2 sandwich ELISA systems based on an anti–IL-33 coating antibody and a detection antibody specific for either dsDNA, the most prevalent nucleic acid in SLE ICs, or neutrophil myeloperoxidase (MPO). Using both assays in independent patient cohorts, serum samples from patients with SLE exhibited increased levels of IL-33–complexed NETs as compared with those of healthy counterparts (Figure 2, A and B). Serum IL-33 NET concentration correlated significantly with patient disease activity assessed by the validated SLE Disease Activity Index (SLEDAI) (35) (Figure 2, A and B), and longitudinal reduction in serum IL-33/MPO complexes was noted in patients with good clinical response to belimumab treatment (36) (Supplemental Figure 1A; supplemental material available online with this article; https://doi.org/10.1172/jci.insight.147671DS1). To validate our technique, and in agreement with previous reports (32, 37), we also detected increased NETs containing neutrophil MPO (MPO/dsDNA complexes) in SLE versus healthy sera (Supplemental Figure 1B).

Lastly, we purified blood neutrophils and used confocal microscopy to characterize spontaneous NETs release. Consistent with the serum data, SLE neutrophils generated IL-33 NETs, whereas healthy neutrophils did not release NETs and displayed minimal IL-33 expression (Figure 2C). We also detected IL-33 decoration on NETs from SLE low-density granulocytes (Supplemental Figure 1C), a subset of pathogenic neutrophils with enhanced NETosis potential. In line with the immunofluorescence observations, *IL33* mRNA levels were elevated in neutrophils from patients with SLE versus healthy individuals (Figure 2D). Collectively, SLE neutrophils release IL-33 NETs in the blood and other inflamed tissues, such as the kidneys and skin, suggesting their implication in the disease.

### SLE ICs were potent inducers of IL-33–containing NETs.

Soluble factors in the serum contribute to the NETotic potential of SLE neutrophils (14, 32). By culturing healthy neutrophils in medium supplemented with serum derived from patients with SLE who were positive for anti-dsDNA and anti-ribonucleoprotein autoantibodies, we noted production of IL-33–decorated NETs and upregulation of *IL33* mRNA (Supplemental Figure 2, A–C). Within lupus serum, ICs induce NETs through activation of neutrophil Fcγ receptors (FcRs) and TLR-7 receptors (14). To address this, neutrophils were pretreated with FcR-blocking reagent or the TLR-7 antagonist IRS661, followed by incubation with lupus serum. Inhibition of either receptor led to reduced production of IL-33–containing NETs and decreased *IL33* mRNA levels (Supplemental Figure 2, A**–**C), consistent with a mediating effect of ICs.

**Figure 3:**
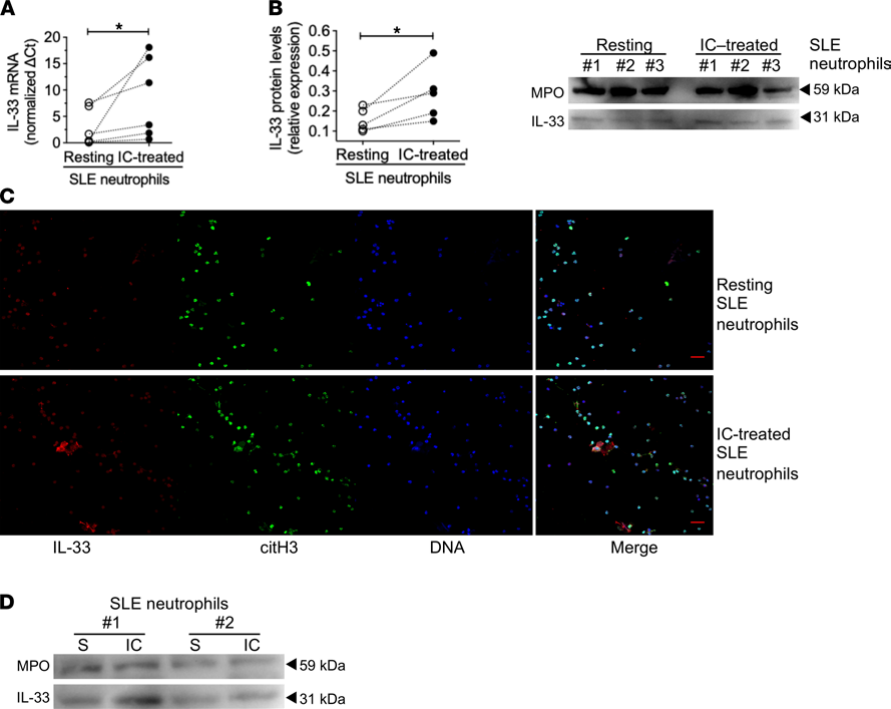
Stimulation of neutrophils, derived from patients with SLE, with nucleic acid-containing ICs led to increased IL-33 expression and IL-33 decoration of NETs. (**A**) Real-time PCR was performed to confirm differential gene expression of *IL33* in resting versus IC-treated neutrophils from the peripheral blood of patients with SLE (*n* = 6). Data were normalized using the average value of resting neutrophils ΔCt (*IL33* Ct minus GAPDH Ct) values. Each dot represents a different donor and bar plots show the mean ± SEM expression. *P < 0.05 (2-tailed, paired *t* test). (**B**) Western immunoblotting was performed to examine intracellular IL-33 protein in unstimulated and IC-treated neutrophils from patients with SLE (*n* = 5). Results were normalized and quantified via densitometry followed by calculation of the relative expression of IL-33 over MPO (loading control). Each dot represents a different donor and bar plots show the mean ± SEM expression. *P < 0.05 (2 tailed, paired *t* test) (left panel). Representative blot (*n* = 2 experiments) from unstimulated and IC-treated neutrophils obtained from 3 patients (right panel). (**C**) Unstimulated or IC-treated SLE neutrophils were cultured for 3 hours and then stained with anti–citrullinated histone-3, anti–IL-33 (IL-33) antibodies, and DAPI for DNA. Representative confocal images (scale bar: 30 μm) in 1 of 3 replicates demonstrate that IC-treated SLE neutrophils generate abundant amounts of IL-33–decorated NETs. (**D**) Western immunoblotting for IL-33 protein in spontaneously released versus IC-mediated NETs precipitates derived from SLE neutrophils (*n* = 3).

Next, we sought to examine the effect of ICs directly on neutrophils and in relation to the production of IL-33 NETs. By culturing SLE neutrophils with nucleic acid–containing ICs generated as previously described (38), we observed increased NET production (Supplemental Figure 2D). In confirmation of our experimental setup, IC-induced NETs stained positive for 8-Oxo-2′-deoxyguanosine (8-OH-dG; a marker of oxidized DNA), TOMM20 (an outer-membrane mitochondrial protein), and citrullinated histone 3 (a marker indicative of the NOX-independent pathway of NETosis), which are all hallmarks of SLE NETosis (16) (Supplemental Figure 3, A and B). In contrast, resting (unstimulated), healthy neutrophils displayed minimal IL-33 and 8-OH-dG expression, and TOMM20 staining was cytoplasmic (Supplemental Figure 3A). SLE neutrophils exposed to ICs exhibited increased intracellular *IL33* mRNA and protein levels (Figure 3, A and B), notwithstanding the low IL-33 abundance in myeloid cells (39, 40). Accordingly, both confocal microscopy and immunoblotting in precipitated NETs revealed elevated IL-33 protein levels in IC-released versus spontaneously released SLE NETs (Figure 3, C and D), whereas SLE ICs, per se, contained no measurable IL-33 (Supplemental Figure 4A). Contrary to SLE neutrophils, healthy neutrophils produced IL-33 NETs only after priming with exogenous recombinant IFN-α followed by administration of ICs (Supplemental Figure 4B) (13, 14), which reiterates the role of the SLE milieu in determining the neutrophil NETotic potential. Furthermore, when comparing NOX-dependent (namely, phorbol 12-myristate 13-acetate–treated [PMA-treated] healthy neutrophils) versus NOX-independent (namely, monosodium urate–treated [MSU-treated] healthy neutrophils, IC-treated SLE neutrophils) NETs, only the latter exhibited significant IL-33 decoration (Supplemental Figure 3B), thus suggesting a mechanistic link between NOX-independent pathway of NETosis and IL-33 release. Conclusively, nucleic acid–containing IC stimulation of SLE neutrophils recapitulates typical lupus NETosis and is associated with increased IL-33 expression.

### SLE NETs activated pDCs to produce type I IFN in an ST2L-dependent manner.

In SLE, IC-treated neutrophils externalize NETs that trigger pDCs to produce type I IFN (14–16). Besides the nucleic acid content, NETs interferogenic capacity is also regulated by the protein cargo, thus the NET complex is a potent mediator of the characteristic dysregulated immune response in lupus (41). We examined whether IL-33 contributes to the interferogenic effect of IC-induced SLE NETs.

First, we assessed the expression of ST2L on the membrane of peripheral blood CD123^+^ pDCs by flow cytometry and confirmed that healthy and SLE pDCs are already receptive of IL-33 signaling (Supplemental Figure 5, A and B). We administered NETs derived from IC-treated SLE neutrophils to healthy pDCs that had been pretreated with an inhibitory anti-ST2L antibody (aST2L) to block IL-33 signaling. pDCs were pretreated with FcR blocker to minimize any IC carryover or nonspecific aST2L effects.

**Figure 4:**
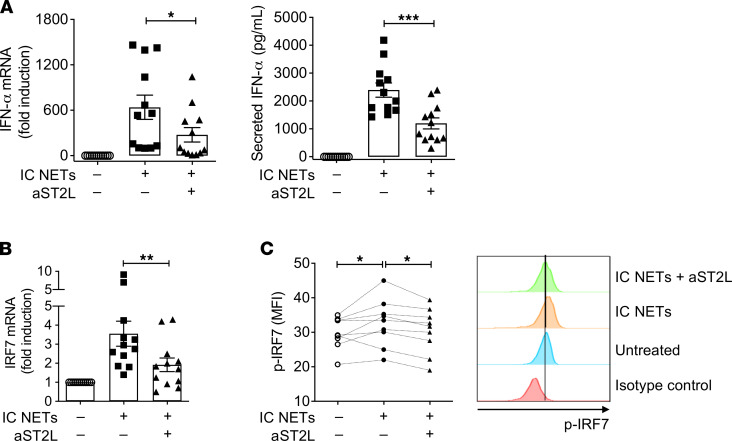
SLE NETs induced a robust IFN-α response by pDCs in an ST2L-dependent manner. (**A**) Real-time PCR (left panel) and ELISA (right panel) were performed to determine mRNA and protein expression or secretion of IFN-α, respectively, by healthy pDCs treated overnight with IC-induced SLE NET-containing supernatants (IC NETs) (25% v/v). The contribution of the IL-33/ST2L axis on IFN-α response was assessed by pretreating pDCs with an antibody against ST2L (a-ST2L, 3 μg/mL). FcR blocking reagent was used to avoid any IC-carry over effect or non-specific a-ST2L binding. Each dot represents a different pDC donor (*n* = 12) and bar plots show the mean ± SEM expression. *P < 0.05; ***P < 0.001 (2-tailed, repeated measures ANOVA with Holm-Sidak correction). (**B**) Real-time PCR for *IRF7* mRNA expression in pDCs (*n* = 12 healthy donors) stimulated with IC NETs (25% v/v), with or without pre-treatment with FcR blocking agent and a-ST2L, as described for **A**. Quantification was performed using the 2^-ΔΔCT^ method, where ΔCt = *IRF7* Ct minus *GAPDH* Ct. **P < 0.01 (2-tailed, repeated measures ANOVA with Holm-Sidak correction). (**C**) Intracellular p-IRF7 staining was performed 4 hours after stimulation of purified pDCs with IC NETs (25% v/v), with or without pretreatment with FcR blocking agent and a-ST2L, as described for **A**. Each dot (open circle, full circle, triangle) corresponds to the kinetics of p-IRF7 in 9 independent donors, and bar plots show the mean ± SEM expression. *P < 0.05 (2-tailed, repeated measures ANOVA with Holm-Sidak correction). *Right panel* illustrates a representative flow cytometry histogram of intracellular p-IRF7 levels in pDCs treated as described for **A**. MFI, mean fluorescence intensity.

In agreement with findings in previous reports (14–16), SLE IC NETs induced robust *IFNA* mRNA and protein expression, which was significantly ameliorated upon ST2L blockade (Figure 4A). A similar effect was noted with the mRNA levels of *IRF7* (IFN regulatory factor 7), a characteristic IFN-stimulated genes (Figure 4B). Likewise, SLE IC NET-containing supernatants pretreated with anti–IL-33 blocking antibody displayed significantly reduced inteferogenic capacity (Supplemental Figure 5C), thus further supporting the role of the IL-33/ST2L axis on NET-mediated IFN-α production by pDCs. Of note, PMA-induced NETs released from healthy neutrophils induced a less profound type I IFN response (such as *IFNA* and *IRF7* mRNA expression) by pDCs, which was not significantly altered by ST2L blockade (Supplemental Figure 5D).

IFN production by pDCs is mediated through activation of TLR-7 and TLR-9, with cytosolic receptors playing an auxiliary role (14–16). To gain insights into the intracellular events linking IL-33–complexed NETs with IFN-α production by pDCs, we examined the levels of phosphorylated IRF7 (p-IRF7), which is essential for the expression of type I IFN genes downstream to TLR signaling. Treatment of pDCs with SLE IC NETs caused upregulation of p-IRF7, which was reversed following ST2L blockade (Figure 4C), therefore reiterating the role of the IL-33/ST2L axis in SLE NETs’ interferogenic effect.

To discern the relative effects of NET-associated DNA and IL-33, we cultured pDCs using cytochalasin D, which blocks endocytosis of NETs, or chloroquine, disrupting endosomal TLR trafficking. With either treatment, IFN-α response by pDCs was attenuated (Supplemental Figure 5E), suggesting that the NET DNA scaffold is essential for the immunostimulatory effects of IL-33. In line with this, treatment of pDCs with an exogenous cytokine isoform (18 kDa) of IL-33 alone failed to induce significant IFN-α production (Supplemental Figure 5F). These results indicate that IL-33 associated with SLE NET DNA may interact with ST2L on pDCs to regulate type I IFN through IRF7.

### Loss of IL-33 impaired the interferogenic potential of SLE-like NETs.

**Figure 5:**
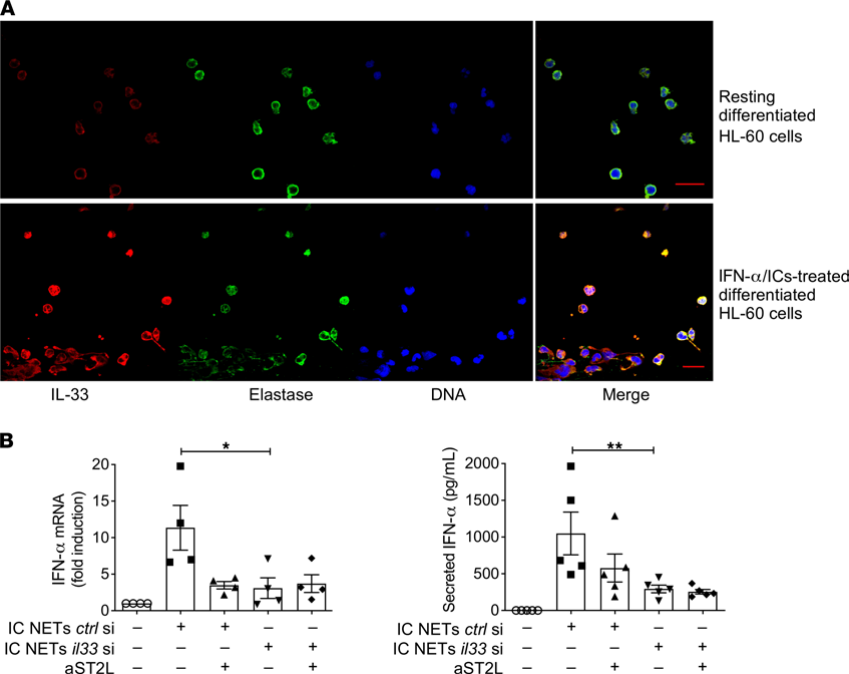
*IL33* silencing impaired the interferogenic potential of NETs produced by neutrophil-like cells cultured under lupus-mimicking conditions. (**A**) Retinoic acid–differentiated, neutrophil-like HL-60 cells were primed with recombinant IFN-α (2000 U/mL for 1 hour) and treated with SLE ICs for 3 hours or left untreated, followed by staining with anti–IL-33 (IL-33) antibody, anti-elastase (Elastase) antibody, and DAPI for DNA. HL-60 cells produced IL-33–decorated NETs as illustrated in the representative confocal image (out of *n* = 3 experiments; scale bar: 30 μm). (**B**) Real-time PCR (left panel) and ELISA (right panel) to monitor IFNA mRNA and IFN-α protein expression or secretion, respectively, by pDCs cultured with IC NETs (25% v/v) derived from control-silenced (ctrl si; *scramble*) or *IL33*-silenced (*IL33* si) differentiated HL-60 cells. The contribution of the IL-33/ST2L axis to IFN-α response was assessed by pretreating pDCs with a-ST2L (3 μg/mL) for 45 minutes. FcR blocking reagent was used to avoid any IC-carryover effect or nonspecific a-ST2L binding. Each dot represents an independent replicate (*n* = 4 [number of donors included]and *n* = 5 [number of donors used in **C**]) and bar plots show the mean ± SEM expression. *P < 0.05; **P < 0.001 (2-tailed, repeated measures ANOVA with Holm-Sidak correction).

To provide direct evidence for the implication of IL-33 in the interferogenic capacity of SLE NETs, we pursued gene-silencing experiments. Due to the short lifespan of primary neutrophils, we differentiated the HL-60 myeloid cell line into neutrophil-like cells using a retinoic acid–based protocol (42). Differentiated HL-60 cells expressed characteristic neutrophil markers (Supplemental Figure 6A), and following priming with recombinant IFN-α and treatment with SLE ICs, they produced NET-like structures, as previously described (43), which were decorated with IL-33 (Figure 5A). We performed *IL33* gene silencing in neutrophil-like HL-60 cells, which resulted in efficient knockdown of IL-33 protein (Supplemental Figure 6, B and C) without affecting the release and DNA content of IC-induced NETs (Supplemental Figure 6D). Notably, NETs derived from HL-60 cells transfected with scramble siRNA induced significant amounts of *IFNA* mRNA and protein by healthy pDCs in an ST2L-dependent manner, thus recapitulating the effects of SLE NETs (Figure 5B). In contrast, NETs from *IL33*-silenced neutrophil-like cells lost their interferogenic capacity, and ST2L blockade had no additional effect (Figure 5B). These data support that the IL-33/ST2L axis is critical for the interferogenic potential of SLE IC NETs.

### SLE IC–induced NETs were enriched in IL-33 isoforms with putative enhanced bioactivity.

**Figure 6:**
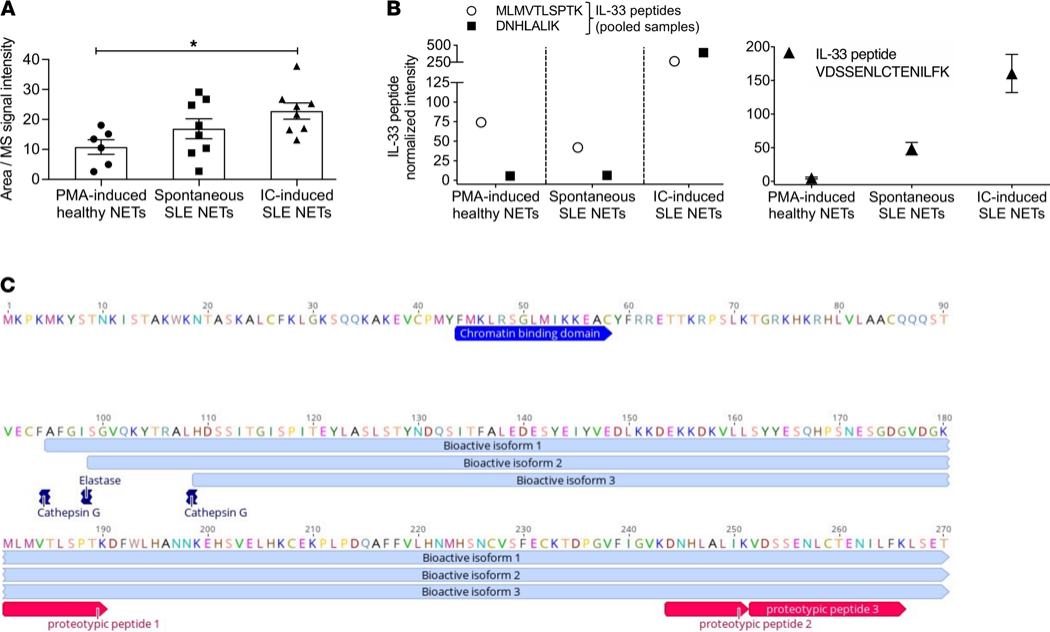
Proteomic analysis in IC-induced SLE neutrophil-derived NETs revealed enrichment in IL-33-proteotypic peptides. (**A**) IL-33–targeted proteomic analysis (parallel reaction monitoring, PRM) in NETs protein precipitates from PMA-treated healthy neutrophils (PMA-induced healthy NETs), unstimulated SLE (Spontaneous SLE NETs), and IC-treated SLE (IC-induced SLE NETs) neutrophils. The signal intensity of the IL-33 proteotypic peptide -VDSSENLCTENILFK[aa251-265] was higher in IC-induced SLE NETs. Signal quantification was performed based on the area of the corresponding peptide peaks in the ion chromatograms. Each dot represents the quantification values from different donors (*n* = 6–8 in each group) and bar plots show the mean ± SEM expression. *P < 0.05 (2-tailed, 1-way ANOVA). (**B**) Proteomic analysis was repeated in pooled NETs from an independent cohort of PMA-treated healthy (*n* = 6), resting SLE (*n* = 8), and IC-treated SLE (*n* = 8) neutrophils. Quantification was performed as described for **A**, and data were normalized by dividing the IL-33 peptide signal intensity by the normalized signal intensity (ppm) of MPO (derived from the whole proteome and used as an indicator of NETosis) in each sample. In the left panel, intensities of the -MLMVTLSPTK[aa180-189] (open circle) and -DNHLALIK[aa243-250] (full square) IL-33–proteotypic peptides are shown. Using the proteomic analysis of individual samples shown in **A**, we also calculated the mean ± SEM normalized intensity (using the respective MPO ppm values) of the IL-33 proteotypic peptide -VDSSENLCTENILFK[aa251-265] in the same groups (right panel). (**C**) Amino acid sequence of (fl)IL-33 is depicted using Geneious Prime software. Nuclear localization domain, NET protease cleavage sites, previously detected bioactive isoforms, and proteotypic peptides derived from our proteomic analysis are annotated.

Our findings suggest that NET-complexed IL-33 from IC-treated lupus neutrophils drives IFN-α production by pDCs. IL-33 exists in chromatin-associated, full-length nuclear form that can be cleaved by neutrophil serine proteases into highly bioactive forms (18, 44, 45). We asked whether IC SLE NETs contain cleaved IL-33 isoforms not detectable by immunoblotting, due to their low abundance. We performed parallel reaction monitoring mass spectrometry in NET-precipitated proteins from untreated and IC-treated SLE neutrophils, as well as from healthy neutrophils undergoing PMA-induced NETosis (*n* = 6–8 individuals in each group) (Supplemental Tables 2–4). In line with the immunofluorescence data, IC-treated SLE NETs demonstrated the highest intensity of the IL-33 proteotypic peptide VDSSENLCTENILFK (aa 251–265) (Figure 6).

We next designed the same focused proteomic analysis using pooled NET proteins to obtain a richer proteomic profile and gain additional insights on possible NET protease–mediated IL-33 cleavage and activation. We collected NET-associated proteins from a new cohort of the same 3 groups of neutrophils followed by targeted IL-33 peptide quantification (normalized over MPO intensity to allow for comparative analyses). Through this approach, we identified 2 additional IL-33 proteotypic peptides, namely MLMVTLSPTK (aa 180–189) and DNHLALIK (aa 243–250), which displayed increased levels in IC-induced versus untreated SLE NETs and PMA-treated healthy NETs (Figure 6B). Importantly, all 3 IL-33 peptides found to be upregulated in SLE IC NETs were localized near the C-terminus, which exists in the bioactive isoforms of IL-33 and accounts for their signaling potential (17, 18), and not in the N-terminus, which is found only in the full-length protein (Figure 6C). Together, these results suggest that IC-induced SLE NETs may be enriched in cleaved IL-33 isoforms with putative enhanced bioactivity.

### NET proteases were implicated in the generation of interferogenic NET-complexed IL-33.

**Figure 7:**
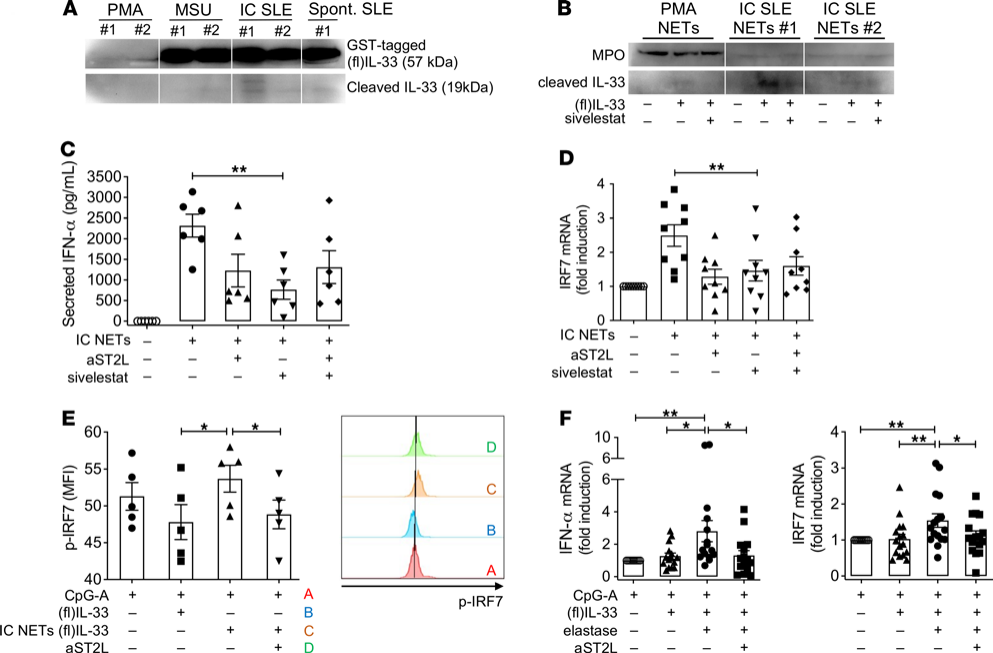
Neutrophil elastase cleaved NET-bound IL-33 into highly interferogenic bioactive isoforms. (**A**) Recombinant (fl)IL-33 was added to cultures of PMA-treated, MSU-treated healthy, unstimulated, or IC-treated SLE neutrophils. After 3 hours, DNase (200 U/mL, 30 minutes, 37°C) was added and NET-proteins were harvested. Immunoblotting revealed band approximately equal to 19 kDa corresponding to protease-generated IL-33 isoform in the supernatants from IC-treated SLE neutrophils. (**B**) The same assay was repeated using cultures of IC-treated SLE neutrophils pretreated (or not) with sivelestat (2 μΜ). A representative blot of 2 experiments is shown. (**C**) SLE neutrophils were stimulated with ICs, followed by addition of sivelestat. NETs were collected and administered to pDCs. Supernatants were assayed by ELISA for IFN-α. Each dot represents a different donor (*n* = 5) and bar plots show the mean ± SEM expression. **P < 0.01 (2-tailed, repeated measures (RM) ANOVA with Holm-Sidak correction). (**D**) The same experiment as in **C** was repeated and pDCs were assayed for *IRF7* mRNA (*n* = 9 donors) **P < 0.01 (2-tailed, RM-ANOVA with Holm-Sidak correction). (**E**) pDCs were cultured with CpG-A (0.1 μΜ) and either (fl)IL-33 (100 nM) or supernatants from the incubation of IC-treated SLE neutrophils with (fl)IL-33 for 4 hours. IL-33 NET-cleaved supernatants were treated with DNAse. pDCs were assayed by flow cytometry for intracellular p-IRF7 mean fluorescence intensity (MFI). *Left panel* summarizes the results from 5 donors represented by different dots, and *right panel* illustrates a representative flow cytometry histogram. *P < 0.05 (2-tailed, RM-ANOVA with Holm-Sidak correction). (**F**) pDCs were cultured with CpG-A and supernatants (2.5% v/v) derived from the in vitro reaction of (fl)IL-33 (1.8 μg/mL) with or without elastase (50 ng/mL). pDCs were assayed for *IFNA* (left panel) and *IRF7* (right panel) mRNA (*n* = 16 donors). *P < 0.05; **P < 0.01 (2-tailed, RM-ANOVA with Holm-Sidak correction). Spont., spontaneous.

IC SLE NETs are enriched in serine proteases, including elastase and cathepsin G, which are potent IL-33 activators (14, 18). Driven by our proteomic data, we hypothesized that NETs may act as a platform for neutrophil proteases to target NET-bound IL-33, thus generating bioactive isoforms that augment the interferogenic potential of SLE NETs. To evaluate this possibility, and due to the low endogenous level of IL-33, we administered recombinant (fl)IL-33 in cultures of IC-treated NETotic neutrophils from patients with SLE. As a control, we used spontaneously NETotic SLE neutrophils and PMA- or MSU-induced NETotic neutrophils from healthy donors, all supplemented with the same amount of (fl)IL-33. Immunoblotting in the supernatants revealed an approximately 19 kDa protein band in SLE NETting neutrophils, especially the IC-treated ones, which might correspond to protease-generated IL-33 isoforms (18) (Figure 7A).

To address more directly the implication of neutrophil proteases in the interferogenic properties of NET-bound IL-33, we blocked neutrophil elastase using the selective inhibitor sivelestat, optimizing the inhibitor concentration and timing of administration to minimize any interference with the NETotic process (Supplemental Figure 7A). We repeated the (fl)IL-33 plus NETs mixture assay using IC-stimulated SLE NETting neutrophils, which were pretreated with sivelestat. Immunoblotting of the sivelestat-treated supernatants showed reduced abundance of the approximately equal to 19 kDa protein band of putative bioactive IL-33 isoforms (Figure 7B), thus suggesting diminished elastase-mediated cleavage of (fl)IL-33. Following treatment with sivelestat, IC-treated SLE NETs abolished their capacity to induce IFN-α response by pDCs (Figure 7, C and D), implying that NET-bound IL-33 signaling is abrogated due to lack of protease-mediated activation. A similar effect was observed using an inhibitor of cathepsin G (Supplemental Figure 7B), another NET-associated neutrophil protease that mediates IL-33 cleavage.

To further assess IL-33 bioactivity, we administered SLE NET-cleaved or unprocessed (fl)IL-33 to cultures of pDCs, which were stimulated with the TLR-9 ligand CpG-A. Supernatants derived from the incubation of IC-treated SLE NETting neutrophils with (fl)IL-33 caused a significant, ST2L-dependent increase in p-IRF7 levels of pDCs, suggesting enrichment in bioactive IL-33, whereas an equivalent dosage of uncleaved (fl)IL-33 had the opposite effect (Figure 7E). Furthermore, SLE NET-processed IL-33-containing supernatants promoted *IFNA* and *IRF7* mRNA expression by CpG-A-activated pDCs (Supplemental Figure 7C).

Finally, to recapitulate in vitro the elastase and IL-33 interaction presumed to occur on SLE NETs, we incubated (fl)IL-33 with recombinant elastase to obtain supernatants enriched in bioactive IL-33 (Supplemental Figure 7D). Notably, CpG-A–treated pDCs supplemented with bioactive (elastase-treated) IL-33–containing supernatants showed significant upregulation of *IFNA* and *IRF7* mRNA expression, as compared with the effect of unprocessed recombinant (fl)IL-33 (Figure 7F). These results suggest that neutrophil proteases play a critical role in augmenting IC SLE NETs’ interferogenic capacity through cleavage-mediated IL-33 activation.

## Discussion

A poorly explained feature of SLE is unabated type I IFN signaling that spurs autoreactive responses and persists even during clinical remission (2, 8). pDCs are a major source of IFN-α, and lupus-prone mice with defective pDC-mediated IFN response have reduced autoantibody formation, reduced lymphadenopathy, and prolonged survival (46). Perpetual production of IFN-α in SLE is triggered by self-derived nucleic acids complexed with autoantibodies or immunostimulatory proteins, such as in the form of NETs (13–15). We focused on IL-33, a chromatin-bound alarmin with context-specific immunomodulatory effects, and evaluated its role in human SLE. Driven by our observation that neutrophils infiltrate and release IL-33–bearing NETs in the blood and other inflamed tissues of patients with SLE, we herein demonstrate that lupus neutrophils are prone to producing IL-33 NETs, which induce a robust IFN-α response by pDCs through the ST2L receptor. Importantly, our NETs proteome data, coupled with results of ex vivo inhibition assays, implicate NET proteases in the generation of bioactive IL-33 of high interferogenicity, thus offering what we believe are novel mechanistic insights linking neutrophil activation and NETosis with IFN-α and end-organ injury in human SLE.

Extracellular IL-33 augments immune responses during tissue inflammation and injury; however, its precise role in autoimmunity remains elusive. We show that IL-33 is externalized on SLE NETs and contributes to their interferogenic capacity through ST2L on pDCs. This is in line with findings of a previous study suggesting that chromatin binding regulates ST2-mediated bioactivity of IL-33 (47). Possible explanations for the adjuvant effect of NET-complexed IL-33 include protecting the NET structure from degradation (15) or facilitating NET uptake through ST2L (48). IL-33/ST2L axis can both enhance (49) and inhibit (50) TLR signaling in a context-specific fashion, and IL-33 induces IRF7 expression in innate lymphoid cells (51), which might represent a mechanism by which NET-complexed IL-33 renders pDCs more responsive to immunostimulatory DNA. IL-36, another IL-1 family cytokine, induces IFN-α production by facilitating endosomal TLR trafficking in pDCs (52). Of interest, previous studies have indicated cross-talk signaling between cyclic GMP–AMP synthase–stimulator of interferon genes (cGAS-STING), known to be triggered by NET DNA (53), and IL-33/ST2L in the context of allergic airway inflammation (54, 55), therefore raising the hypothesis that NET-associated IL-33 might also amplify non-TLR cytoplasmic nucleic acid–sensing signaling pathways.

IL-33 complexed with or processed by SLE NETs, but not the recombinant cytokine form, was capable of inducing robust IFN-α production by pDCs. This raised the possibility that IL-33 may be modified by NETs to gain biological activity. Indeed, IL-1 family cytokines require proteolytic processing for activation, and IL-33 can be cleaved into highly bioactive isoforms by neutrophil proteases (18, 44, 45). However, the topology of this process in the setting of autoimmunity has not been demonstrated. To this end, our proteomic analysis of SLE IC NETs revealed upregulation of 3 distinct IL-33 peptides exclusively localized near the C-terminal cytokine domain, which exists in mature IL-33 isoforms (17, 18). In this regard, SLE neutrophils and their NETs exhibit a potent serine protease signature (14); moreover, our proteomic analysis revealed the absence of a major endogenous inhibitor of elastase (SerpinB1) from IC SLE NETs (Supplemental Tables 2–4). Accordingly, inhibition of elastase and cathepsin G in NETting SLE neutrophils abrogated the IL-33–mediated interferogenic effect of NETs. Our data are in line with findings implicating NET protease–processed IL-1β in gout attacks (56) and of neutrophil protease-generated IL-33 in acute lung injury (18), pointing to neutrophil proteases generating NET-associated IL-33 with high interferogenic activity in the context of lupus.

Limited studies have detected proteolytically processed forms of IL-33 in biological samples because characterization of human extracellular IL-33 has been challenging due to its low abundance. Genetic evidence supports that even small changes in IL-33 expression are implicated in disease susceptibility (57). Under inflammatory conditions, neutrophils are rapidly recruited into afflicted tissues, and the expression level of alarmins may increase significantly, reaching locally high concentrations. Intriguingly, under high neutrophil densities, NETs may accumulate (58) and, presumably, act as platforms for the extracellular scavenging and processing of IL-33 released by neighboring damaged epithelial and endothelial cells (18, 45).

Previous work has suggested that oxidation of IL-33 limits its bioactivity, due to formation of disulfide bridges that impede the IL-33/ST2L interaction (59). Since neutrophil activation and NETosis in SLE occur under oxidative stress driven by mitochondrial ROS (16, 60), it is of interest to discern how NET-associated IL-33 may retain its bioactivity. Notably, oxidized isoforms have not been detected in nuclear (DNA-bound) IL-33 (59), which could indicate that the NET-DNA scaffold or histones are protective against oxidation. Also, according to published data (61) and our proteomics analysis, SLE NETs are decorated with molecules that display reducing capacity, including thioredoxin reductase, peroxiredoxin-2, and glutathione S-transferase, which might counteract IL-33 oxidation. Finally, if IL-33 abolishes NET protection extracellularly, it is possible that it exerts its biological effects acutely before its oxidation, pertinent to the close proximity of NETting neutrophils and pDCs in lupus-inflamed tissues (62, 63).

Our group (2, 32) and others (13, 33, 64) have associated neutrophils and aberrant NET formation with lupus nephritis, but the implication of specific alarmins externalized on SLE NETs to the kidney disease is not well understood. Our ex vivo functional assays, coupled with evidence that pDCs infiltrate the inflamed kidneys in SLE (62), raise the possibility that IL-33–decorated NETs trigger intrarenal type I IFN production in lupus nephritis, as recently suggested by single-cell transcriptomic studies (65). Interestingly, we found that IL-33 NETs were predominantly localized within the tubulointerstitium, and tubulointerstitial inflammation has been correlated with pDC infiltrates (66) and poor prognosis in lupus nephritis (67). IL-33 exhibits myeloid cell chemoattractant properties (68), thus it might also attract neutrophils to orchestrate a neutrophil/NETs IL-33/IFN-α feedback loop within lupus-inflamed kidneys.

Considering that IL-33–decorated NETs were also detected in SLE cutaneous lesions, it is possible that these structures can be sensed by skin-infiltrating pDCs, thus contributing to the profound type I IFN signature observed in lupus skin (69). This finding corroborates those of previous studies showing that kidney and skin tissue of patients with SLE share common genomic perturbations, including upregulated type I IFN-inducible genes (65).

Although we focused on the role of IL-33 in regulating the interferogenic capacity of NETs, it is possible that IL-33 might contribute to SLE through other mechanisms. Thus, IL-33–bearing NETs might interact with other ST2L-expressing immune cell types such as Th2, Tregs, or NK cells (17, 19). In addition, the IL-33/ST2L axis has been shown to promote fibrosis under inflammatory conditions (70).

Our results have potential implications for the design of novel therapeutics to counteract aberrant IFN-α production in SLE and other relevant pathologies such as infection-induced autoinflammatory pneumonitis (71). Since IL-33 is released by activated neutrophils undergoing NETosis, targeting IL-33/ST2L might be beneficial in selectively neutralizing excessive IFN-α response without inducing generalized immunosuppression. Anti–IL-33 and/or aST2L antibodies are currently under clinical development (72); in a preliminary report, administration of anti–IL-33 antibody in MRL/*lpr* lupus–prone mice reduced anti-dsDNA and IC levels, kidney inflammation, and proteinuria (31).

In conclusion, we provide evidence that SLE NET-derived IL-33 processed by neutrophil proteases may contribute to disease pathogenesis by augmenting IFN-α production by pDCs. IL-33–bearing NETs infiltrating lupus end organs such as the kidneys and skin suggest they play an important role in regulating local autoimmune inflammation and tissue injury. Accordingly, IL-33 and/or its maturation process represent druggable targets toward ameliorating excessive IFN-α production and SLE pathology.

## Methods

### Patients.

We included patients with SLE followed at the Rheumatology Clinic, University Hospital of Heraklion (Heraklion, Greece), who met the 1997 American College of Rheumatology revised classification criteria (73) (Supplemental Table 1). Active SLE was defined according to an SLEDAI 2000 score (SLEDAI-2K) equal to or greater than 6 (74) on the day of sampling. Clinical response to treatment was evaluated according to the SLE Responder Index 4 (75). Patients abstained from their medications for at least 12 hours prior to blood-sample collection.

### Isolation and culture of blood neutrophils.

Human peripheral blood neutrophils were isolated as previously described (32). Cell viability was measured at 99% by trypan blue dye exclusion. Neutrophils were cultured with no phenol red RPMI medium (catalog 11835030; Gibco) supplemented with 1% volume per volume (v/v) heat-inactivated FBS and 10 mm HEPES (catalog 15630080; Gibco) in a humidified 37°C and 5% CO_2_ incubator. Serum from patients with SLE and healthy individuals was used at 10% v/v final concentration. To inhibit serum IC-mediated effects, neutrophils were pretreated for 45 minutes with FcR-blocking reagent (Miltenyi Biotec) or the TLR-7 antagonist oligonucleotide IRS661 (1 μM, 5′-TGCTTGCAAGCTTGCAAGCA-3′; Invitrogen).

### HL-60 differentiation and gene silencing.

The HL-60 promyelotic cell line, provided by Despoina Aggouraki (Laboratory of Translational Oncology, School of Medicine, University of Crete, Heraklion, Greece) was cultured in RPMI-1640/l-glutamine (catalog 21875-034; Gibco), supplemented with 10% v/v heat-inactivated FBS, 100 IU/mL penicillin, 100 μg/mL streptomycin, and 10 mm HEPES (Gibco) in a humidified 37°C 5% CO_2_ incubator. Exponentially growing cells (1 × 10^6^) were incubated with 1 μM ATRA (all-trans retinoic acid; catalog R2625; Sigma-Aldrich). After 6 days, flow cytometry was used to monitor the expression of neutrophil markers (CD11b, CD66b, CD16). For gene silencing, we collected 4 × 10^6^ cells/sample on day 4 of differentiation, which were electroporated with 25 nM ON-TARGETplus IL-33 siRNA (catalog L-HUMAN-XX-0005; Dharmacon) and ON-TARGETplus Non-targeting siRNA (catalog D-001810-01-05; Dharmacon) using the Amaxa Nucleofector Kit V (catalog VCA-1003; Lonza Bioscience) and program T019. Cells were incubated in serum-free medium for 6 hours to recover and then were cultured in complete medium plus 1 μM ATRA for 2 days. Immunofluorescence and Western immunoblotting were used to evaluate silencing efficiency on day 6.

### Generation, isolation, and quantification of NETs.

SLE neutrophils (1 × 10^6^) or differentiated HL-60 cells were seeded in 12-well tissue-culture plates and cultured with no phenol red RPMI (Gibco) supplemented with 1% v/v heat-inactivated FBS, 5 μM 2-mercaptethanol (catalog 31350010; Gibco) and 10 mm HEPES (Gibco), for 3 hours in a humidified 37°C 5% CO_2_ incubator. ICs, provided by Lars Rönnblom and Maija-Leena Eloranta (Department of Medical Sciences, Rheumatology and Science for Life Laboratory, Uppsala University, Uppsala, Sweden), were prepared as previously described (76). Differentiated HL-60 cells were primed with 2000 U/mL Universal IFN-α (catalog PBL11200-2; PBL Assay Science) for 1 hour prior to stimulation with lupus ICs. Supernatants were discarded, cells were carefully washed once with prewarmed medium, and NET–containing supernatants were vigorously collected and centrifuged at 150*g* for 5 minutes at 4°C to obtain cell-free supernatants. Inhibitors of elastase (2 μM sivelestat; catalog S7198; Sigma-Aldrich) or Cathepsin G (20 μM; catalog 219372; Calbiochem) were added after 75 minutes of neutrophil stimulation.

In NET-mediated cleavage experiments, recombinant glutathione-*S*-transferase–tagged, (fl)IL-33 (100 nM; catalog H00090865; Abnova) was added after 2 hours of neutrophil stimulation for 1 hour. PMA (100 nM; catalog P1585; Sigma-Aldrich) and MSU crystals (100 μg/mL; catalog tlrl-msu; Invivogen) were used as controls. Both cell supernatants and NETs were precipitated for Western blot analysis. To quantify NET-containing supernatants, we used the SYTOX Green Nucleic Acid Stain Kit (catalog S7020; Invitrogen) according to the manufacturer’s instructions. NETs were quantified as previously described (77). Briefly, decondensed nuclei or filamentous DNA structures stained for both DAPI and MPO were considered NETs. We used the FIJI software (ImageJ2; NIH) and assessed 5 randomly selected coverslip fields for each treatment and 3 biological replicates (independent experiments) to calculate the proportion of NET-releasing cells in the total number of cells.

### Isolation and culture of pDCs.

Buffy coats from healthy donors were used for isolation of peripheral blood mononuclear cells by density-gradient centrifugation (650*g*, 22°C, 30 minutes) (catalog MST00T41004; Lymphosep). pDCs were obtained with the CD304 (BDCA-4/Neuropilin-1) MicroBead Kit, human, isolation kit (catalog 130-090-532; Miltenyi Biotec) at a purity greater than 95%. pDCs were cultured in RPMI-1640/l-glutamine supplemented with 10% v/v heat-inactivated FBS, 100 IU/mL penicillin, 100 μg/mL streptomycin, and 10 mm HEPES (Gibco) for up to 18 hours. Prior to stimulation with NETs (25% v/v NET-containing supernatants or cleaved IL-33–containing supernatants) and CpG-A (0.1 μM; catalog tlrl-2216; Invivogen), pDCs were pretreated with aST2L (3 μg/mL; catalog AF523; R&D) and FcR blocking reagent (catalog 130-090532; Miltenyi Biotec) to minimize any IC carryover effect and nonspecific binding of aST2L. IC SLE NET-containing supernatants were pretreated with goat anti–human IL-33 antibody (4 μg/mL; catalog AF3625; R&D) or left untreated at 37°C for 45 minutes before their administration to pDC cultures. pDCs were also pretreated with cytochalasin D (5 μg/mL; catalog C2618; Sigma-Aldrich) or chloroquine (4 μM; Plaquenil, ATC code 8P01BA02; Sanofi Aventis) for 30 minutes to block endocytosis and TLR trafficking, respectively.

### In vitro cleavage of (fl)IL-33.

Bioactive IL-33–containing supernatants were generated as previously described (45). Specifically, reactions consisting of recombinant elastase (50 ng/mL; catalog 324681; Calbiochem) and recombinant (fl)IL-33 (1.8 μg/mL; Abnova) were carried out (15 μL final volume) in protease reaction buffer (50 mM HEPES [pH 7.4], 75 mM NaCl, 0.1% CHAPS [3-([3-cholamidopropyl] dimethylammonio)-1-propanesulfonate], 5 μM 2-mercaptethanol) for 2 hours at 37°C. The reaction was stopped by adding sivelestast (2 μM). As a control, recombinant (fl)IL-33 was incubated for 2 hours at 37°C in protease reaction buffer without recombinant elastase.

### Immunofluorescence.

Neutrophils or differentiated HL-60 cells were seeded on coverslips coated with 0.001% poly-l-lysine (catalog P8920; Sigma-Aldrich) and cultured for 3 hours at 37°C and 5% CO_2_. Cells were fixed with 4% paraformaldehyde, blocked with 5% weight per volume (w/v) BSA/PBS buffer and permeabilized with 0.2% Triton X-100 for 6 minutes. DNA was stained with 300 nM DAPI (catalog D9542; Sigma-Aldrich). IL-33 protein staining (goat antibody [1:50 dilution], catalog AF3625, R&D; mouse “Nessy” antibody [1:250 dilution] for verification, catalog ALX-804-840-C100, Enzo) was performed at 4°C overnight. NET protein staining was performed using anti-citH3 (1:200 dilution; catalog ab5103; Abcam), anti–neutrophil elastase antibody (1:200 dilution; catalog ab21595; Abcam), and anti-MPO (1:350 dilution; catalog A0398; Dako) primary antibody for 1 hour at room temperature, followed by 1-hour incubation with Alexa488-conjugated (catalog A-11008; Molecular Probes), CF555-conjugated (catalog 20039; Biotium) and CF633-conjugated (catalog 20121; Biotium) secondary antibodies (1:750 dilution). Anti-TOMM20 (1:150 dilution; catalog NBP1-81556; NovusBio) and anti–8-OHdG (1:150 dilution; catalog sc-393871; Santa Cruz Biotechnology) were also used. Three washes with 0.5% w/v BSA/PBS were performed between all stainings. After staining, coverslips were mounted on Mowiol 4-88 (catalog 81381; Sigma-Aldrich) and were observed with confocal microscopy (×40 objective; model SP8; Leica). Confocal images were analyzed with the FIJI software.

Paraffin-embedded renal and skin sections from patients with active SLE and healthy donors were cut at 3 μm, mounted, and dried on Superfrost Plus slides (Thermo Fisher Scientific). After dewaxing and rehydration, the sections were incubated in citrate-based HIER (heat-induced epitope retrieval) buffer at 60°C in a water bath for 60 minutes. After antigen retrieval, the sections were left in the respective citrate buffer at room temperature to cool below 30°C, rinsed with deionized water 3 times, then with TBS once at pH 7.4, and permeabilized for 8 minutes with 0.2% Triton X-100 in TBS, followed by 3 rinsing steps with TBS and blocking with 5% w/v BSA/TBS. Immunostaining was conducted as detailed previously. The same tissue sections were also stained with H&E and evaluated using a Nikon Eclipse E-400 light microscope (×400 magnification).

### Western blot.

Cells or NET-precipitated proteins were lysed on ice in SDS lysis buffer (2% SDS, 62.5 mM Tris at pH 6.8, 5% 2-mercaptoethanol, 10% glycerol) supplemented with Complete Roche Inhibitor Cocktail (Complete tablets Mini Easypack [catalog 04693124001] and PhosSTOP Easypack [catalog 04906837001]) and centrifuged at 13,000*g* for 15 minutes at 4°C. Whole-cell lysates (20–30 μg protein) were subjected to SDS-PAGE electrophoresis on 12.5% gels and then transferred to an Immobilon-PSQmembrane (catalog SEQ85R; Millipore). Membranes were blocked with 5% skimmed milk in TBS plus Tween 20 and then incubated with anti-MPO (1:3000 dilution; Dako) as loading control (78) or anti–IL-33 (1:1000 dilution) specific antibodies (mouse “Nessy” antibody; R&D). For HL-60 protein extracts, mouse anti–human tubulin antibody was used as loading control (1:3000 dilution, catalog A11126; Thermo Fisher Scientific). Detection was performed using relevant HRP-linked antibodies (anti–goat-HRP, anti–rabbit-HRP, anti–mouse-HRP; catalog AP180P, 12-348, and 12-349, respectively; all purchased from Millipore) and enhanced chemiluminescent-detection reagents (Amersham Biosciences, RPN2109). Unedited gels are provided in the online supplement.

### NET protein precipitation for mass spectrometry.

Neutrophils (4–7 × 10^6^) were stimulated using PMA (100 nM; Sigma-Aldrich) for 3 hours at 37°C. SLE neutrophils were stimulated using ICs (as described in *Generation, isolation, and quantification of NETs*) or left unstimulated (for 3 hours at 37°C) to release NETs spontaneously. Supernatants were carefully removed, cells were washed twice, and NETs were harvested using DNase (20 U/mL; catalog 4716728001; Roche) in HBSS (catalog 14025; Thermo Fischer Scientific) for 20 minutes. Supernatant-containing NETs were isolated via centrifugation (400*g*, 4°C, 10 minutes). NETs proteins were precipitated using 80% v/v acetone overnight at –20°C. Precipitated pellet was obtained via centrifugation at 10,000*g* for 30 minutes. Then, precipitated proteins were washed twice using an acetone-based buffer (80% v/v acetone, 10 mm Tris-HCl at pH 8). Protein pellets were stored at –80°C until mass spectrometry analysis.

### Mass spectrometry.

Detailed descriptions of sample preparation, mass spectrometry and data analysis, including total proteome and targeted proteomic analyses, are presented in Supplemental Methods.

### Flow cytometry.

Cells were stained for extracellular markers for 20 minutes at 4°C in PBS and 5% FBS. For phosphoprotein staining (PE-conjugated mouse anti–p-IRF7 antibody; catalog K47-671; BD Biosciences), cells were permeabilized and stained using the Intracellular Fixation and Permeabilization Buffer Set Kit (catalog 88-8824-00; eBioscience). Conjugated antibodies against CD123, CD303, CD15, CD14, HLA-DR, and CD10 were from BioLegend (catalog 306012, 354208, 301906, 325604, 307628, 312210, respectively). Ig isotype controls were used in all assays.

### Quantitative real-time PCR.

Total RNA from neutrophils or pDCs was collected using the TRIzol (catalog 15596026; Invitrogen) extraction protocol followed by Turbo DNase (catalog AM2238; Ambion) treatment according to the manufacturer’s instructions. cDNA was prepared using PrimeScript 1st Strand cDNA Synthesis Kit (catalog RR037A; Takara Bio). Transcripts were quantified by incorporation of the KAPA SYBR FAST qPCR Kit Master Mix (catalog KK4602; Kapa Biosystems) in a CFX Connect Real-Time PCR System (BIO-RAD). Expression levels were normalized to *GAPDH* or *HPRT1* and calculated by the change-in-threshold method; that is, 2^–ΔΔCT^. Primer sequences are listed in Supplemental Table 5.

### ELISA.

NET-associated IL-33/dsDNA complexes were quantified by a modified capture ELISA. Specifically, 1 μg/mL mouse anti–human IL-33 (“Nessy”) antibody (Enzo) was coated overnight in 96-well microtiter plates. After blocking with 1% w/v BSA and 1% v/v donkey serum, human serum (1:6 dilution) was added together with a peroxidase-labeled anti-DNA monoclonal antibody in the Cell Death ELISAPLUS Kit (1:25; catalog 11774425001; Roche). The plate was incubated for 2.5 hours, shaking at 300 rpm at room temperature. After 3 washes with PBS, peroxidase substrate (ABTS) was added to incubate at room temperature in the dark for 20 minutes. Absorbance at 405 nm wavelength was measured (490 nm was used as a reference filter). IL-33/MPO complexes were quantified using a goat anti–human IL-33 antibody (R&D) for coating (1:200 dilution) and a mouse anti–human MPO antibody (catalog HM1051; Hycult Biotech) for detection (1:100 dilution). After 3 washes with PBS, anti–mouse HRP antibody (1:10,000 dilution; catalog AP308P; Millipore) was added for 1 hour at room temperature. After 5 washes with PBS, 3,3’,5,5;-tetramethylbenzidine (TMB; catalog 002023; Invitrogen) was added and light absorbance was measured at 450 nm (540 nm was used as a reference filter). Detection of IFN-α (Human IFN Alpha Multi-Subtype ELISA Kit; catalog PBL41110-1; PBL Assay Science) in culture supernatants was performed by precoated sandwich ELISA. Light absorbance at 450 nm was measured using the ELx800 microplate reader (Biotek). Background signal was subtracted. All samples were assessed in duplicate.

### Statistics.

Data were analyzed using GraphPad Prism 8 software. Results are presented as the mean ± SEM and/or dot plots. Data normality was assessed by the Shapiro-Wilk’s test to guide selection of parametric or nonparametric tests. In assays including multiple conditions or treatments, repeated measures 1-way ANOVA was used, followed by the post hoc Holm-Sidak test to correct for multiple comparisons. The association between IL-33 serum complexes (i.e., IL-33/dsDNA, IL-33/MPO) and SLE disease activity (determined using the SLEDAI-2K) was assessed by the Spearman’s rank correlation coefficient. *P* < 0.05 (2-tailed) was considered statistically significant.

### Study approval.

The study was approved by the Ethics Committee of the University Hospital of Heraklion, and all participants gave written informed consent (protocol no. 5944/14-6-2017).

## Author contributions

SG designed and performed experiments, interpreted and analyzed the data, generated the figures and wrote parts of the manuscript. KG, GP, ED, DTB, and PS provided critical feedback, helped shape the research plan and reviewed and edited the manuscript. MLE and LR offered critical reagents and provided useful feedback on the manuscript. HG provided human paraffin-embedded tissue samples from patients with lupus nephritis. JZ analyzed the proteomics experiments and provided feedback on the manuscript. MM conducted and analyzed the proteomics experiments and provided feedback on the manuscript. GK and EB conducted and analyzed the proteomic experiments. PV co-supervised the project, designed experiments, interpreted data, and provided critical feedback on the manuscript. GB designed and supervised the study, recruited and evaluated patients with SLE, interpreted and analyzed data, generated the figures, and wrote the manuscript.

## Supplementary Material

Supplemental data

## Version 1 09/23/2021

In-Press Preview

## Version 2 11/08/2021

Electronic publication
