# Supplementary material for: NETs decorated with bioactive IL-33 infiltrate inflamed tissues and induce IFN-α production in patients with SLE
Source: JCI Insight. 2021 Nov 8;6(21):e147671. doi: 10.1172/jci.insight.147671 (PMC8663547; doi:10.1172/jci.insight.147671)
Supplement: Supplemental data [file jciinsight-6-147671-s137.pdf]

Supplemental Material

Manuscript:

NETs decorated with bioactive IL-33 infiltrate inflamed tissues and induce IFNα production in SLE patients

Supplemental Methods

Mass spectrometry

Sample preparation

Samples were homogenized in lysis buffer consisting of 7M urea, 2M thiourea, 4% w/v CHAPS, 1% w/v DTE, and 2% v/v IPG ampholytes. Samples were sonicated for 10 minutes in a water bath sonicator followed by centrifugation at 16.000 rcf for 20 min at room temperature to collect supernatants. Protease inhibitors (3.6% v/v) were added to the extracts and the protein concentration was determined by the Bradford assay.

Samples were processed with the GeLC-MS method as previously described (1). Briefly, 10μg of the samples were loaded on SDS PAGE (5% stacking, 12% separating) and the electrophoresis was stopped when the samples just entered the separating gel. Gels were fixed with 30% v/v methanol, 10% v/v acetic acid for 30 min at room temperature, followed by wash with water (3 × 10 min washes). Gels were stained with coomassie colloidal blue stain overnight at room temperature. After washing the gels with water, protein bands were excised and were sliced into small pieces (1-2mm). Gel pieces were destained with 40% acetonitrile, 50mM NH_4HCO_3, reduced with 10mM DTE in 100mM NH_4HCO_3 for 20 min at room temperature, and alkylated with 10mg/mL iodoacetamide in 100mM NH_4HCO_3 for 20 min at room temperature, in the dark. After alkylation, the samples were washed with 100mM NH_4HCO_3 followed by another wash with 40% acetonitrile, 50mM NH_4HCO_3 and a final wash with ultra-pure water was performed (20 min, at room temperature for each wash). Gel pieces were dried in a centrifugal vacuum concentrator (speed vac). Trypsinization was performed overnight at room temperature in the dark. Six hundred ng of trypsin was added per sample (10ng/μL tryspin stock solution in 10mM NH_4HCO_3, pH 8.5). Finally, the peptides were extracted after incubation with 50mM NH_4HCO_3 for 15min, room temperature, followed by two incubations with 10% formic acid, acetonitrile (1:1) for 15 min, room temperature. The peptide solution was filtered with PVDF filters

(Merck Millipore) and was dried in a centrifugal vacuum concentrator. Samples were stored at -20°C until further use.

LC-MS/MS analysis

Samples were resuspended in 10µL mobile phase A (0.1% FA). A 5µL volume was injected into a Dionex Ultimate 3000 RSLS nano flow system (Dionex, Camberly, UK) configured with a Dionex 0.1 × 20 mm, 5 µm, 100 Å C18 nano trap column with a flow rate of 5 µL / min. The analytical column was an Acclaim PepMap C18 nano column 75 µm × 50 cm, 2 µm 100 Å with a flow rate of 300 nL/min. The trap and analytical column were maintained at 35°C. Mobile phase B was 100% ACN:0.1% formic acid. The column was washed and re-equilibrated prior to each sample injection. The eluent was ionized using a Proxeon nano spray ESI source operating in positive ion mode. For mass spectrometry analysis, a Q Exactive Orbitrap (Thermo Finnigan, Bremen, Germany) was operated in MS/MS mode. The peptides were eluted under a 60 minutes gradient from 2% (B) to 33% (B). Gaseous phase transition of the separated peptides was achieved with positive ion electrospray ionization applying a voltage of 2.5 kV. For every MS survey scan, the top 10 most abundant multiply charged precursor ions between m/z ratio 300 and 2200 and intensity threshold 500 counts were selected with FT mass resolution of 70,000 and subjected to HCD fragmentation. Tandem mass spectra were acquired with FT resolution of 35,000. Normalized collision energy was set to 33 and already targeted precursors were dynamically excluded for further isolation and activation for 5 seconds with 5 ppm mass tolerance.

MS data processing

Total proteome analysis

Raw files were analyzed with Proteome Discoverer 1.4 software package (Thermo Finnigan), using the Sequest search engine and the Uniprot human (Homo sapiens) reviewed database, downloaded on December 15, 2017, including 20,243 entries. The search was performed using carbamidomethylation of cysteine as static and oxidation of methionine as dynamic modifications. Two missed cleavage sites, a precursor mass tolerance of 10 ppm and fragment mass tolerance of 0.05 Da were allowed. False discovery rate (FDR) validation was based on q value: target FDR (strict): 0.01, target FDR (relaxed): 0.05. The intensity of the signal of MPO peptides was normalized for each sample by dividing with the total intensity of identified peptides and converted to ppm. The list of identified proteins is shown in **Supplemental Tables S2–S4**.

Targeted proteomics analysis.

Proteotypic peptides (peptides that uniquely represent the target protein) for IL-33 were selected based on available data from spectral libraries (http://www.nist.gov/) that were imported to the Skyline software for targeted proteomics analysis. Proteotypic peptides were also evaluated with the Protein Basic Local Alignment Search Tool (BLAST, http://blast.ncbi.nlm.nih.gov) and their proteotypicity was further validated. Skyline indicated the precursor ions that were utilized in the PRM (Parallel Reaction Monitoring) method. For each precursor ion, 2-5 transitions were used for the relative quantification. Data analysis was performed with the Skyline software and all chromatograms were manually inspected to ensure the good quality and accurate peak picking. The top signal producing transition was selected as the quantifier transition in all cases, while the remaining transitions were used as qualifier transitions, for accurate peak profile and retention time confirmation. For pooled samples the IL-33 peptide intensity was normalized by dividing with the MPO intensity.

SUPPLEMENTAL FIGURE S1

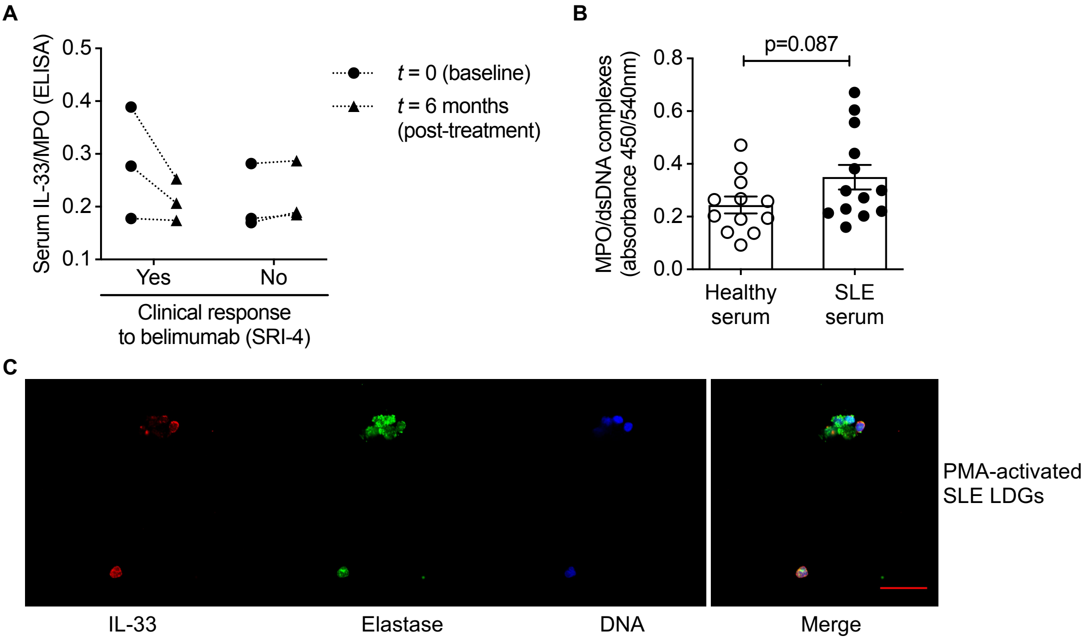

SUPPLEMENTAL FIGURE S2

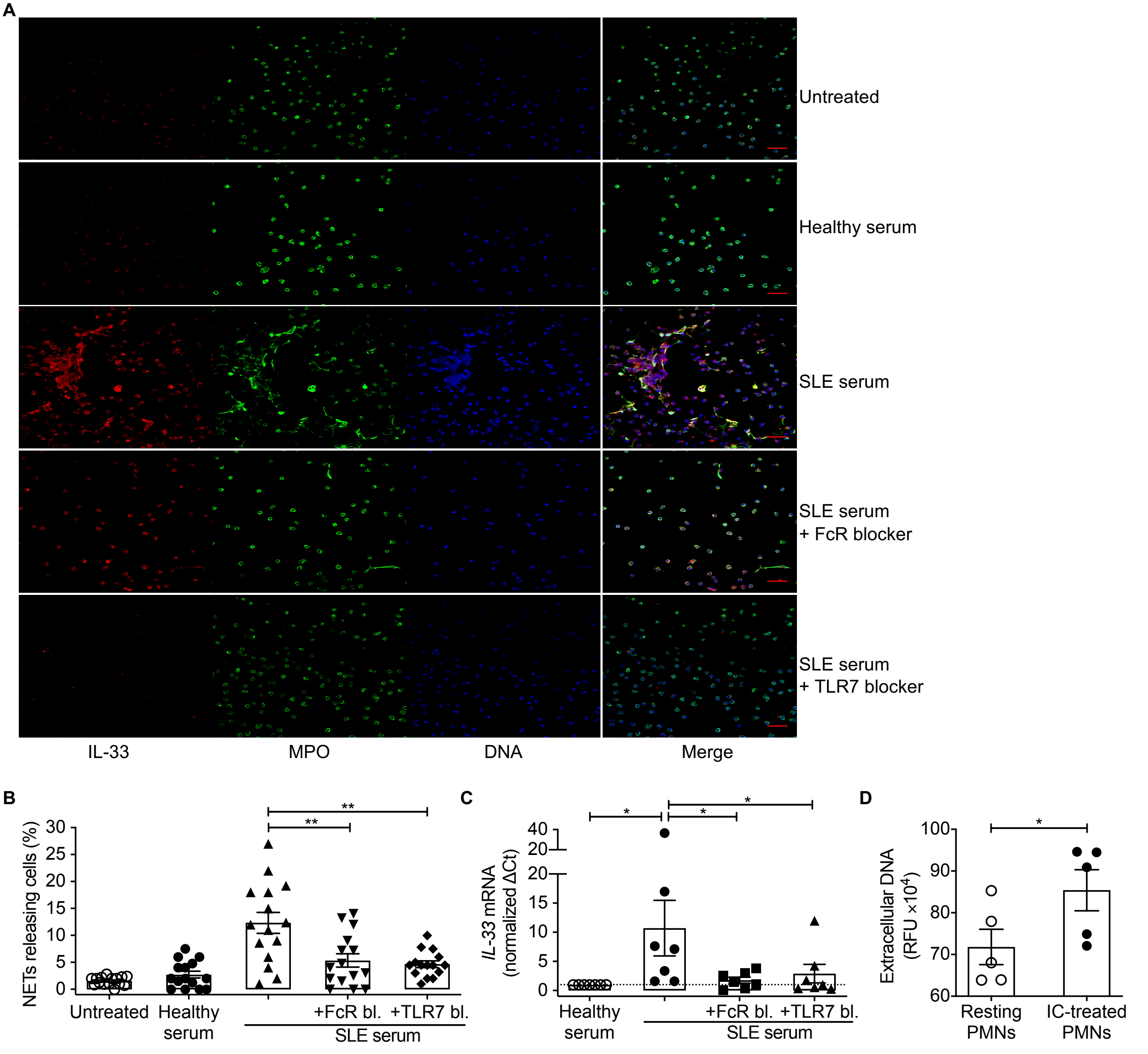

SUPPLEMENTAL FIGURE S3

A

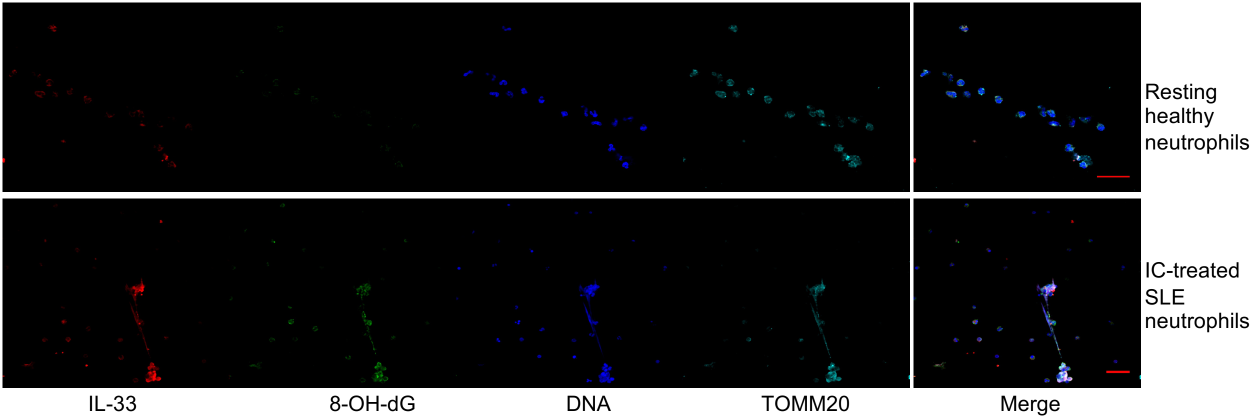

B

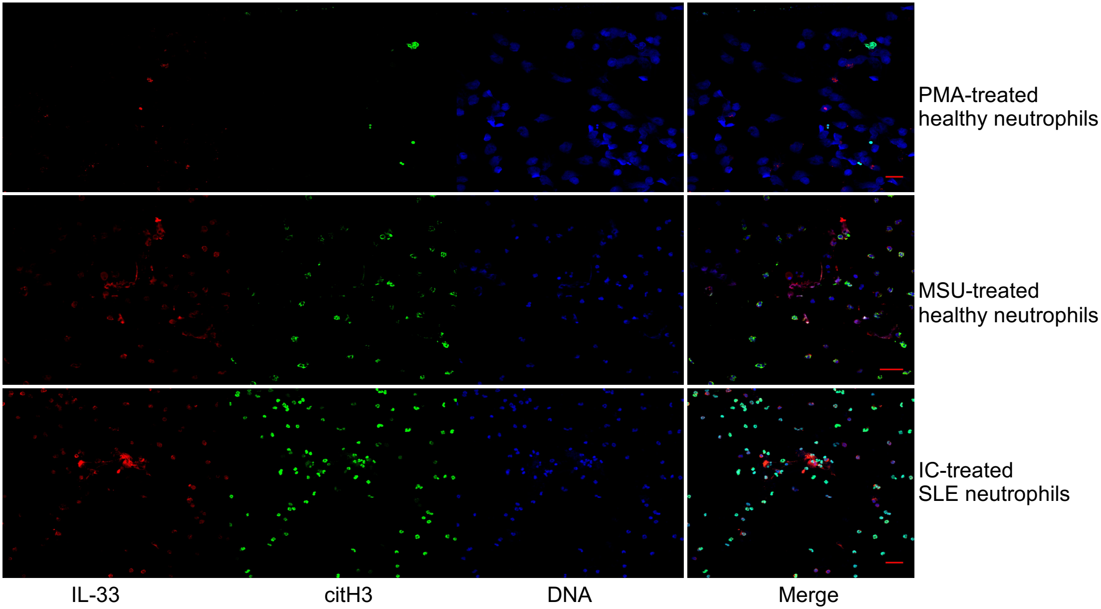

SUPPLEMENTAL FIGURE S4

A

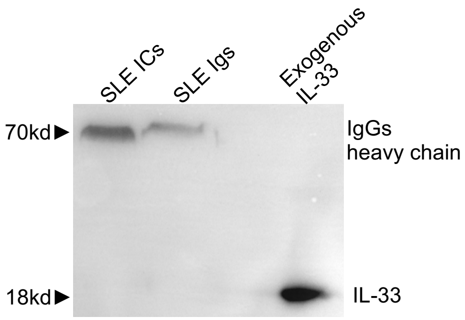

B

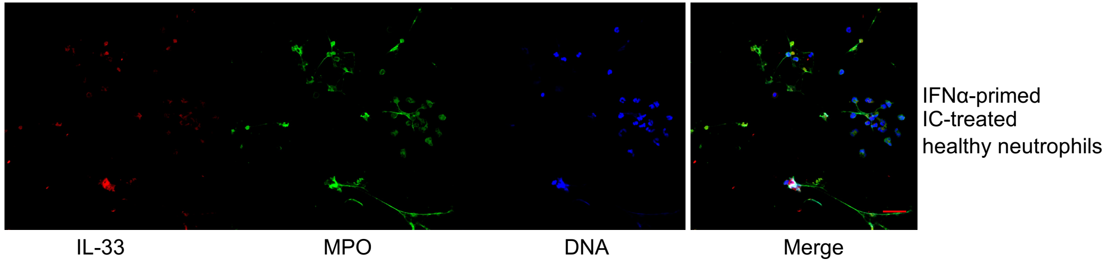

SUPPLEMENTAL FIGURE S5

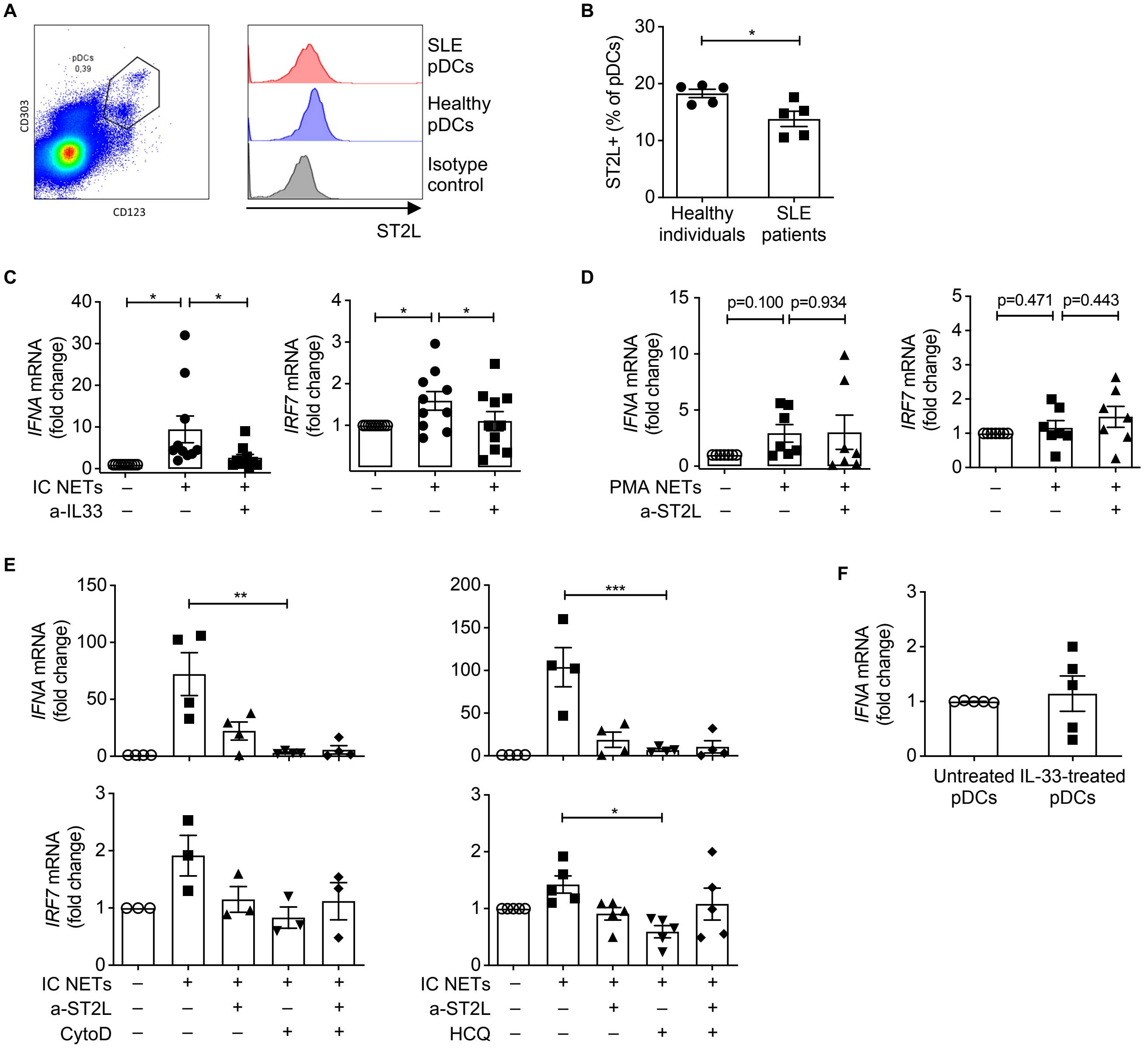

SUPPLEMENTAL FIGURE S6

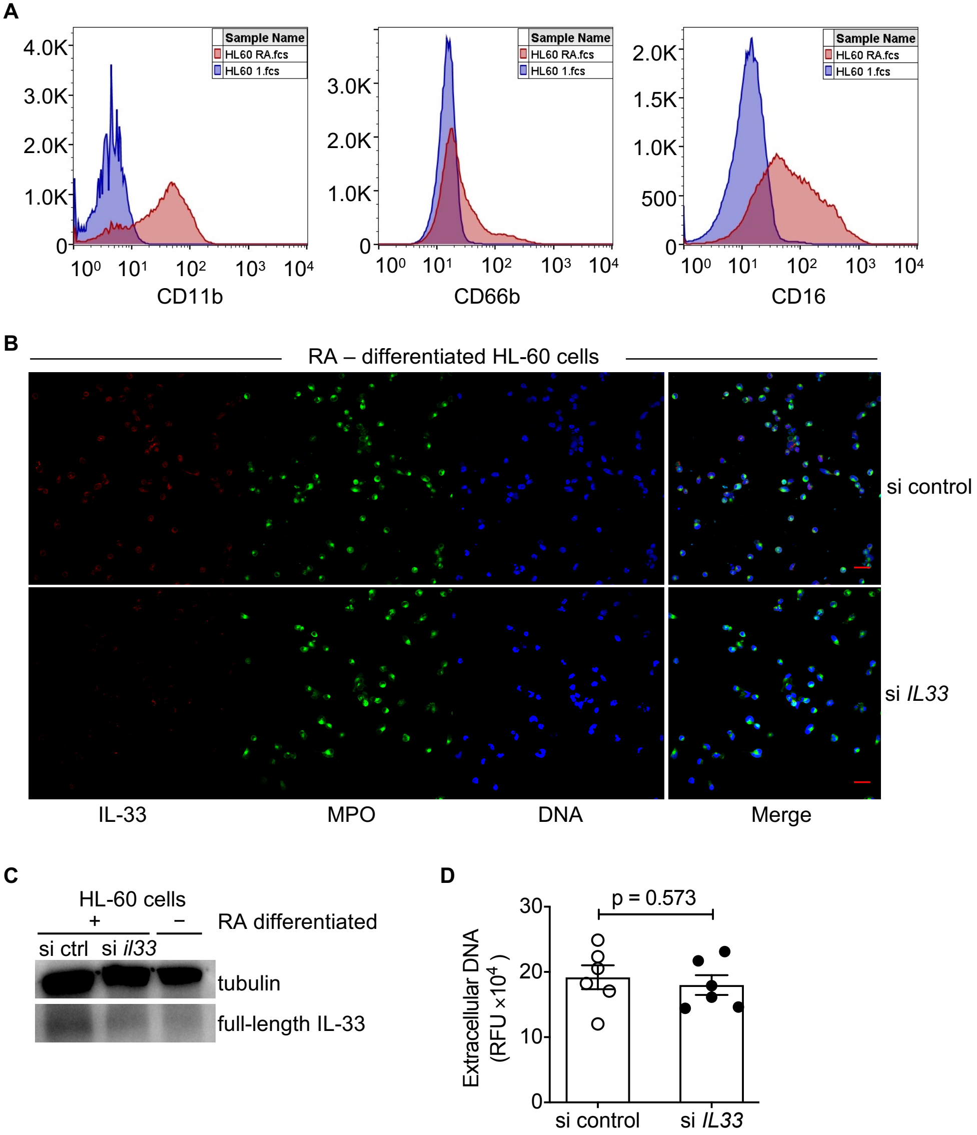

SUPPLEMENTAL FIGURE S7

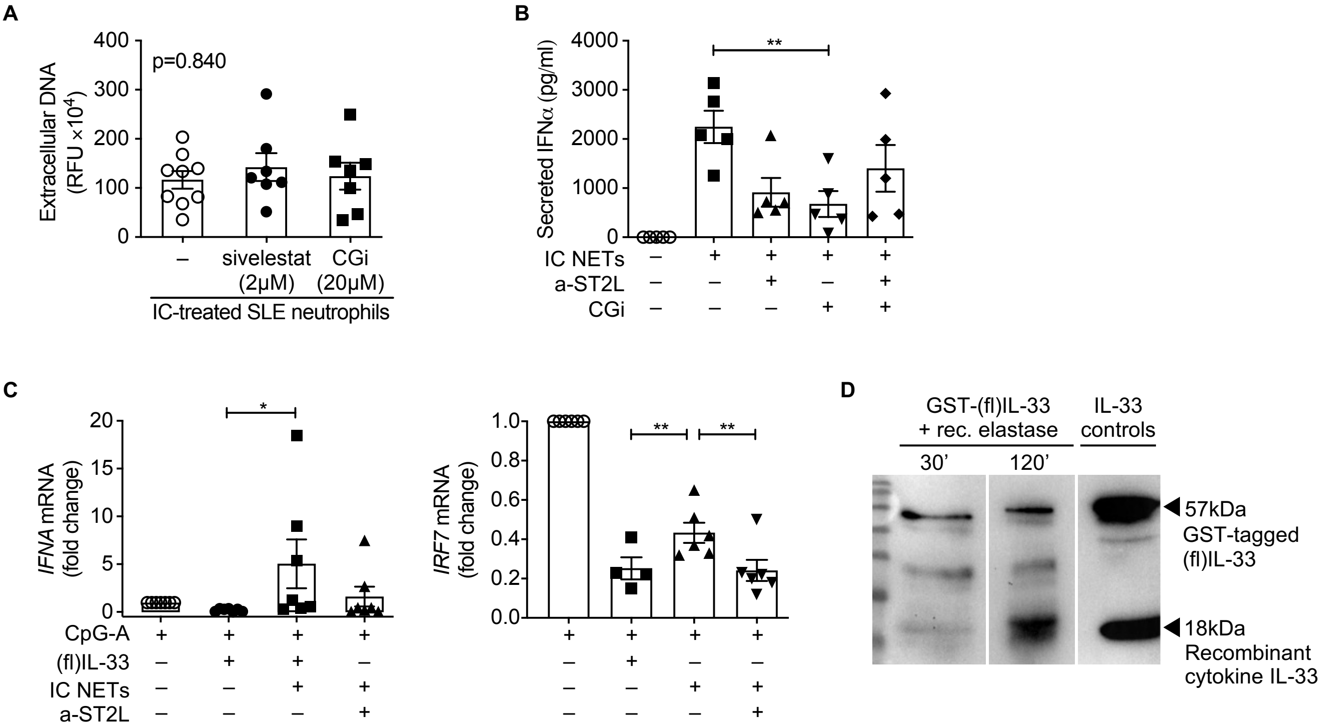

SUPPLEMENTAL FIGURES LEGENDS

Supplemental Figure S1. Serum IL-33–containing NETs in patients with SLE

(A) IL-33/MPO complexes were quantified by sandwich ELISA in paired longitudinal serum samples collected from *n*=6 SLE patients at the time of treatment initiation (t=0) and after 6 months (t=6) of treatment with belimumab. Clinical response to treatment was evaluated according to the validated SLE Responder Index-4 (SRI-4). Each dot represents a different donor tested at the aforementioned time points. Mean ± SEM (standard error of the mean) change (6 months minus baseline) in serum IL-33/MPO concentration was -20.8 ± 9.7% in responders as compared to 5.8 ± 3.0% in non-responders (two-tailed p-value = 0.058, unpaired t-test). **(B)** MPO/double stranded(ds)DNA complexes were detected and quantified by sandwich ELISA in serum samples from healthy donors (*n*=12) and SLE patients (*n*=13). Each dot represents a different donor and bar plots show the mean ± SEM absorbance (450/540nm) of MPO/dsDNA complexes (two-tailed p-value = 0.087, Mann-Whitney test). **(C)** Low-density granulocytes (LDGs) were sorted as $CD15^{+}$ $CD14^{lo}$ $HLA\text{-}DR^{int}$ $CD10^{+}$ cells from the peripheral blood of patients with SLE and cultured in standard medium. At 3 hours, cells were stained with anti-IL-33 (IL-33), anti-elastase (Elastase) specific antibodies and DAPI for DNA staining. Representative confocal image (scale bar, 30μm) from a single patient is shown.

Supplemental Figure S2. SLE serum immunocomplexes induce IL-33-decorated NETs in an FcR- and TLR-7-dependent manner

(A) Healthy blood neutrophils were cultured in serum (10% v/v) obtained from two SLE patients who were positive for anti-dsDNA and anti-RNP autoantibodies for 3 hours, with or without pretreatment with FcR blocking agent or TLR-7 inhibitor (1μM, IRS661) for 45 minutes. Staining was performed with anti-IL-33 (IL-33), anti-myeloperoxidase (MPO) antibodies and DAPI for DNA. Representative confocal images (scale bar, 30μm) show induction of IL-33-containing NETs by SLE serum, which is

reversed upon FcR or TLR-7 blockade (*n*=3 experiments were performed all exhibiting the same pattern). **(B)** Quantification of NETotic cells was conducted using the FIJI software as previously described (2). Each dot represents the percentage of NETotic cells measured in a randomly selected coverslip field (*n*=15) and bar plots show the mean ± standard error of the mean (SEM). **p<0.01 (two-tailed, repeated measures ANOVA with Holm-Sidak correction). **(C)** Real-time PCR was used to assess *IL33* mRNA in healthy neutrophils cultured in presence of healthy serum or serum from two SLE patients who were positive for anti-dsDNA and anti-RNP autoantibodies, with/without FcR or TLR-7 blocking agent as described above. Quantification was performed using the double delta Ct method ($2^{-\Delta\Delta CT}$ where ΔCτ=*IL33* Cτ minus *HPRT1* Cτ). Each dot represents a different neutrophil donor (*n*=7) and bar plots show the mean ± SEM. *p<0.05 (two-tailed, repeated measures ANOVA with Holm-Sidak correction). **(D)** Unstimulated (resting) and immunocomplexes (ICs)-treated neutrophils from SLE patients were assessed for NETs production assayed by the extracellular DNA dye SYTOX Green. Each dot represents the relative fluorescence intensity in neutrophils derived from *n*=4 SLE patients and bar plots show the mean ± SEM. *p<0.05 (two tailed, paired t-test).

Supplemental Figure S3. SLE neutrophils release IL-33–decorated NETs consisting of oxidized mitochondrial DNA in a NOX-independent manner

(A) Resting healthy neutrophils and IC-treated SLE neutrophils were cultured for 3 hours and then stained using anti-8-Oxo-2'-deoxyguanosine (8-OH-dG), anti-TOMM20 (TOMM20, Translocase Of Outer Mitochondrial Membrane 20), and anti-IL-33 (IL-33) antibodies. DAPI was used for DNA staining. Representative confocal images in one of *n*=3 SLE and *n*=2 healthy neutrophils (scale bar, 30μm) are shown. **(B)** Healthy neutrophils were activated with PMA (phorbol myristate acetate, 100nM) or MSU (monosodium urate, 100μg/mL), and SLE neutrophils were activated with ICs as previously described. At 3 hours, cells were stained with anti-IL-33 (IL-33) and anti-citrullinated histone-3 (citH3) antibodies. DAPI was used for DNA staining. Representative confocal images (scale

bar, 30μm) of *n*=3 experiments indicate enhanced production of IL-33-decorated NETs by MSU- and IC-treated neutrophils.

Supplemental Figure S4. Healthy neutrophils release IL-33–decorated NETs upon IFNα priming and administration of SLE immunocomplexes

(A) Western blot was performed in precipitates from SLE ICs and SLE immunoglobulins (IgGs) to assess for possible IL-33 carryover effect. Recombinant cytokine-isoform of IL-33 was used as control. **(B)** Neutrophils from healthy donors were primed with recombinant IFN-a (2000IU/mL, 1 hour) followed by addition of SLE ICs. At 3 hours, cells were stained with anti-IL-33, anti-MPO antibodies and DAPI for DNA. Under these lupus-inducing conditions, neutrophils produced IL-33-decorated NETs. Representative confocal image in one of *n*=5 experiments (scale bar, 30μm) is shown.

Supplemental Figure S5. SLE NETs activate ST2L-expressing pDCs to produce IFNα

(A) pDCs were identified as $CD123^+$ $CD303^+$ peripheral blood mononuclear cells. A representative graph of the gating strategy in a patient with SLE is shown (*left panel*). Membrane ST2L within $CD123^+$ $CD303^+$ pDCs was measured by flow cytometry (representative histogram shown on the *right panel*). **(B)** Following the procedure outlined in (A), the proportion of $ST2L^+$ pDCs was determined in the peripheral blood of healthy individuals (*n*=5) and SLE patients (*n*=5). *$p<0.05$ (two-tailed, independent samples t-test). **(C)** Real-time PCR to quantify *IRF7* and *IFNA* mRNA in healthy pDCs treated overnight with IC-induced NETs-containing supernatants (IC NETs) (25% v/v). The contribution of IL-33/ST2L axis was assessed by pretreating supernatants for 45 min with an antibody against IL-33 (a-IL33, 4μg/ml). FcR blocking reagent was used to avoid any IC-carry over effect or non-specific a-IL33 binding. Each dot represents a different pDC donor (*n*=10) and bar plots show the mean ± standard error of the mean (SEM). *$p<0.05$ (two-tailed, repeated measures(RM-) ANOVA

with Holm-Sidak correction). **(D)** Real-time PCR to determine *IRF7* and *IFNA* mRNA levels in healthy pDCs treated with PMA-induced NETs-containing supernatants (PMA-NETs) (25% v/v) from healthy neutrophils. The contribution of IL-33/ST2L axis was assessed by pretreating pDCs with a monoclonal antibody against ST2L (a-ST2L, 3μg/ml). FcR blocking reagent was used to avoid any non-specific a-ST2L binding. Each dot represents a different pDC donor (*n*=7). Two-tailed p-values are shown (RM-ANOVA with Holm-Sidak correction). **(E)** Real-time PCR to monitor *IFNA* and *IRF7* mRNA in purified pDCs treated with IC-induced SLE NETs (25% v/v). Endocytosis and TLR trafficking were blocked by pre-treating pDCs for 30 min with cytochalasin D (5μg/ml) or chloroquine (4μM), respectively. The contribution of IL-33/ST2L was determined by pretreating pDCs with a-ST2L and FcR blocking reagent was also added. Each dot represents a different donor (*n*=4 for *IFNA*; *n*=3–5 for *IRF7*). **$p<0.01$; ***$p<0.001$ (two-tailed, RM-ANOVA with Holm-Sidak correction). **(F)** Peripheral blood pDCs from *n*=5 healthy donors were cultured for 18 hours in the presence or not of recombinant IL-33 (18 kd cytokine isoform, 100ng/mL). Real-time PCR was performed to monitor *IFNA* mRNA. Each dot represents a different donor and bar plots show the mean ± SEM (two-tailed p-value = 0.673, paired t-test).

Supplemental Figure S6. *IL33* silencing studies in neutrophil-like HL-60 cells

(A) Surface expression of the neutrophil markers CD11b, CD16 and CD66b was assayed by flow cytometry on retinoic acid-differentiated *versus* control (undifferentiated) HL-60 cells. A total *n*=3 replicates were performed and representative histograms from a single experiment are shown. **(B)** Control (scramble) and *il33*-silenced retinoic acid-differentiated HL-60 cells were stimulated using PMA (100nM) for 1 hour followed by staining using anti-IL-33 (IL-33), anti-myeloperoxidase (MPO) specific antibodies and DAPI for DNA. Representative confocal image of *n*=3 experiments (scale bar, 30μm) is shown. **(C)** Protein extracts from the cells used in (B) were obtained and western blot analysis for IL-33 was performed to validate silencing efficiency. Representative blot of *n*=2 experiments is

shown **(D)** NETs-containing supernatants from control (scramble) and *IL33*-silenced retinoic acid-differentiated HL-60 cells were primed with recombinant IFNα (2000U/ml, 1 hour), treated with SLE ICs for 3 hours and then stained with the extracellular DNA dye SYTOX Green. Relative fluorescence intensities were measured from *n*=6 independent replicates. Each dot represents a technical replicate and bar plots show the mean ± SEM (two tailed p-value = 0.573, paired t-test).

Supplemental Figure S7. NET proteases-cleaved IL-33 demonstrates interferogenic potential

(A) SLE patient-derived neutrophils were stimulated with ICs for 75 minutes, followed by addition of the elastase inhibitor sivelestat (2μM) or the cathepsin G inhibitor CGi (20μM). At 3 hours, NETs-containing supernatants were collected and stained using the extracellular DNA dye SYTOX Green. Each dot represents the relative fluorescence intensities from independent blood donors (*n* = 7 to 9 in each condition) and bar plots show the mean ± SEM (standard error of the mean) fluorescence intensity values (p=0.840; two-tailed, repeated measures mixed model to account for missing data). **(B)** SLE neutrophils were stimulated with ICs as above, followed by addition of the cathepsin G inhibitor CGi (20μM). At 3 hours, NETs-containing supernatants were collected and administered (25% v/v) to pDCs as previously described. After overnight culture, supernatants were assayed with ELISA for secreted IFNα protein. Each dot represents a different donor (*n*=5). **p<0.01 (repeated measures ANOVA with Holm-Sidak correction). **(C)** pDCs were cultured with CpG-A (0.1μM) and either full-length (fl)IL-33 (100nM) or supernatants from the incubation of IC-treated SLE neutrophils with (fl)IL-33. The contribution of IL-33/ST2L was determined by pretreating pDCs with a monoclonal antibody against ST2L (a-ST2L, 3μg/ml) and FcR blocking agent was used. NETs-cleaved supernatants were treated with recombinant DNAse (200U/ml) for 30 min at 37°C to minimize NET-DNA carryover. pDCs were assayed for *IFNA* (*n*=7) and *IRF7* (*n*=6) mRNA levels by real-time PCR. Each dot represents a different donor (*n*=9) and bar plots show the mean ± SEM expression. **p<0.01 (two-tailed, repeated measures mixed model to account for missing data). **(D)** Immunoblotting was

employed to verify that in vitro co-incubation (30 min, 2 hours) of recombinant elastase and (fl)IL-33 leads to the generation of a $\simeq$19-kd band resembling the previously described bioactive IL-33 isoform. IL-33 controls (recombinant (fl)IL-33 and recombinant cytokine-isoform IL-33) were loaded (3rd lane) to assess specificity. Representative blot of *n*=3 experiments is shown.

References

1. Makridakis M, and Vlahou A. GeLC-MS: A Sample Preparation Method for Proteomics Analysis of Minimal Amount of Tissue. *Methods Mol Biol.* 2018;1788:165-75.

2. Papadaki G, Kambas K, Choulaki C, Vlachou K, Drakos E, Bertsias G, et al. Neutrophil extracellular traps exacerbate Th1-mediated autoimmune responses in rheumatoid arthritis by promoting DC maturation. *Eur J Immunol.* 2016;46(11):2542-54.

SUPPLEMENTAL TABLES

Supplemental Table S1. Clinical characteristics of SLE patients included in the study

Supplemental Table S2. List of identified proteins (total proteome analysis of pooled samples) in PMA-induced healthy NETs

Supplemental Table S3. List of identified proteins (total proteome analysis of pooled samples) in spontaneously released SLE NETs

Supplemental Table S4. List of identified proteins (total proteome analysis of pooled samples) in IC-induced SLE NETs

Supplemental Table S5. Primer sequences used in qPCR assays

Supplemental Table S1. Clinical characteristics of SLE patients included in the study

No.	Gender	Age	Autoantibodies	Medications	SLEDAI-2K
SLE1	F	31	ANA, anti-Sm, anti-RNP, anti-SSA	HCQ, prednisolone	8
SLE2	F	41	ANA, anti-dsDNA, anti-SSA	prednisolone	14
SLE3	M	32	ANA	-	6
SLE4	F	70	ANA, anti-CCP, anti-SSA	AZA	8
SLE5	F	39	ANA, anti-dsDNA	-	8
SLE6	F	64	ANA, anti-dsDNA	HCQ, MTX, prednisolone	8
SLE7	F	16	ANA, anti-dsDNA, anti-Sm, anti-RNP	HCQ, prednisolone	12
SLE8	F	47	ANA	HCQ, MTX	8
SLE9	F	38	ANA, anti-dsDNA, anti-SSA, anti-cardiolipin IgG	-	8
SLE10	F	35	ANA, anti-Sm, anti-dsDNA, anti-RNP	HCQ, AZA	10
SLE11	M	18	ANA, anti-dsDNA, anti-Sm, anti-RNP	HCQ, prednisolone	18
SLE12	F	73	ANA, anti-dsDNA, RF	MMF, prednisolone	12
SLE13	F	48	ANA, anti-dsDNA, anti-ENA	HCQ, MTX	2
SLE14	F	25	ANA, anti-RNP	HCQ	4
SLE15	F	54	ANA, anti-SSA/SSB, anti-dsDNA	HCQ	10
SLE16	F	57	ANA	HCQ, MTX	2
SLE17	F	19	ANA, anti-dsDNA, anti-Sm, anti-RNP	HCQ, prednisolone	10
SLE18	F	44	ANA, anti-dsDNA	HCQ, AZA	8
SLE19	M	24	ANA, anti-dsDNA, anti-RNP, lupus anticoagulant	HCQ, prednisolone	12
SLE20	F	36	ANA, anti-dsDNA, anti-SSA	HCQ, MTX, prednisolone	8
SLE21	F	19	ANA, anti-dsDNA, anti-SSA/SSB, lupus anticoagulant, anti-cardiolipin IgG/IgM, anti-β2GPI IgG/IgM	Prednisolone	6
SLE22	F	49	ANA	-	8
SLE23	F	56	ANA, anti-dsDNA, anti-SSA	-	6
SLE24	F	56	ANA	MTX, prednisolone	4
SLE25	F	54	ANA	belimumab, MTX, HCQ	6
SLE26	F	62	ANA, anti-dsDNA	AZA, prednisolone	2
SLE27	F	75	ANA, anti-dsDNA	prednisolone	12
SLE28	F	42	-	-	10
SLE29	M	44	ANA, anti-SSA/SSB, anti-Sm, anti-β2GPI IgG	-	8
SLE30	F	35	ANA, anti-dsDNA	HCQ, MTX	6
SLE31	F	38	ANA, anti-dsDNA	HCQ	3
SLE32	F	37	ANA, anti-SSA, anti-RNP, anti-dsDNA, anti-β2GPI IgG/IgM	HCQ	8

SLE33	F	52	ANA, anti-dsDNA	-	8
SLE34	M	51	ANA, anti-SSA, anti-CCP	MTX, thalidomide	6
SLE35	F	74	ANA, anti-dsDNA	-	6
SLE36	F	32	ANA, anti-Sm, anti-SSB	HCQ, prednisolone, CsA	6
SLE37	F	51	ANA, anti-dsDNA, anti-Sm, anti-RNP, anti-JO-1	HCQ, MMF	6
SLE38	F	43	ANA	HCQ, MMF	6
SLE39	F	40	ANA, anti-cardiolipin IgG	-	6
SLE40	F	69	ANA, anti-dsDNA, anti-Sm, anti-SSA, anti-RNP	AZA, prednisolone	5
SLE41	F	37	ANA	HCQ	8
SLE42	F	35	ANA	HCQ	10
SLE43	F	33	ANA, anti-cardiolipin IgG	MTX, HCQ	6
SLE44	F	42	ANA, lupus anticoagulant	MMF, HCQ, prednisolone	6
SLE45	F	47	ANA, anti-dsDNA, anti-Sm	prednisolone	6
SLE46	M	76	ANA, anti-dsDNA	AZA, HCQ, prednisolone	12
SLE47	F	48	ANA	MTX	9
SLE48	F	32	ANA, anti-dsDNA, anti-RNP	HCQ, AZA, prednizolone	16
SLE49	F	87	ANA, C3, C4,	MTX	6
SLE50	F	60	ANA, C4	Belimumab, MTX, HCQ, prednisolone	4
SLE51	F	62	ANA, anti-cardiolipinIgM	Belimumab, HCQ, AZA,	6
SLE52	F	59	C3, C4	MTX, HCQ	6
SLE53	F	57	ANA, anti-dsDNA	MTX, HCQ	10
SLE54	F	36	ANA, C3	Belimumab, MMF, HCQ, prednisolone	1
SLE55	M	16	ANA, anti-dsDNA, C3, C4 anti-SSA, anti-Sm, anti-RNP, άμεση coombs	Belimumab, AZA, HCQ	14
SLE56	F	52	ANA, anti-SSA	AZA, HCQ	10
SLE57	F	47	C3	AZA, HCQ, prednisolone	8
SLE58	F	51	ANA, C3, C4, anti-dsDNA, anti-cardiolipin IgM, antib2GPI IgM, LA	AZA, prednisolone	8
SLE59	F	65	ANA	AZA	12

F, female; M, male; ANA, anti-nuclear antibodies; anti-dsDNA, anti-double stranded(ds)DNA; anti-RNP, anti-ribonucleoprotein; anti-ENA, anti- extractable nuclear antigen antibodies; anti-β2GPI, anti-β2 glycoprotein I; HCQ, hydroxychloroquine; AZA, azathioprine; MTX, methotrexate; MMF, mycophenolate mofetil; CsA, cyclosporin A; SLEDAI-2K, SLE disease activity index-2000

Supplemental Table S2

Accession	Description	Score	Coverage	# Proteins	# Unique Peptides	# Peptides	# PSMs	Area	# AAs	MW [kDa]	calc. pI
P02768	Serum albumin OS=Homo sapiens GN=ALB PE=1 SV=2 - [ALBU_HUMAN]	137,25	6,73	1	5	5	82	8,290E9	609	69,3	6,28
P02788	Lactotransferrin OS=Homo sapiens GN=LTF PE=1 SV=6 - [TRFL_HUMAN]	130,20	47,61	1	27	27	48	4,030E9	710	78,1	8,12
P05109	Protein S100-A8 OS=Homo sapiens GN=S100A8 PE=1 SV=1 - [S10A8_HUMAN]	105,49	41,94	1	6	6	66	8,828E9	93	10,8	7,03
P62805	Histone H4 OS=Homo sapiens GN=HIST1H4A PE=1 SV=2 - [H4_HUMAN]	89,92	41,75	1	5	5	56	6,289E9	103	11,4	11,36
P05164	Myeloperoxidase OS=Homo sapiens GN=MPO PE=1 SV=1 - [PERM_HUMAN]	89,89	38,79	2	22	22	27	1,601E9	745	83,8	8,97
P60709	Actin, cytoplasmic 1 OS=Homo sapiens GN=ACTB PE=1 SV=1 - [ACTB_HUMAN]	83,45	52,53	10	7	16	25	2,140E9	375	41,7	5,48
O60814	Histone H2B type 1-K OS=Homo sapiens GN=HIST1H2BK PE=1 SV=3 - [H2B1K_HUMAN]	75,48	26,98	10	2	4	55	7,088E9	126	13,9	10,32
P06899	Histone H2B type 1-J OS=Homo sapiens GN=HIST1H2BJ PE=1 SV=3 - [H2B1J_HUMAN]	73,93	26,98	8	2	4	56	6,653E9	126	13,9	10,32
P06702	Protein S100-A9 OS=Homo sapiens GN=S100A9 PE=1 SV=1 - [S10A9_HUMAN]	53,79	81,58	1	7	7	20	7,226E9	114	13,2	6,13
P04406	Glyceraldehyde-3-phosphate dehydrogenase OS=Homo sapiens GN=GAPDH PE=1 SV=3 - [G3P_HUMAN]	48,43	47,46	1	10	10	13	7,065E8	335	36,0	8,46
P13796	Plastin-2 OS=Homo sapiens GN=LCP1 PE=1 SV=6 - [PLSL_HUMAN]	44,85	25,04	3	12	12	15	3,954E8	627	70,2	5,43
P0C0S5	Histone H2A.Z OS=Homo sapiens GN=H2AFZ PE=1 SV=2 - [H2AZ_HUMAN]	40,17	7,03	15	1	1	28	6,636E9	128	13,5	10,58
P68871	Hemoglobin subunit beta OS=Homo sapiens GN=HBB PE=1 SV=2 - [HBB_HUMAN]	35,60	71,43	5	9	9	13	1,141E9	147	16,0	7,28
P29401	Transketolase OS=Homo sapiens GN=TKT PE=1 SV=3 - [TKT_HUMAN]	34,97	22,31	1	12	12	13	2,927E8	623	67,8	7,66
P08311	Cathepsin G OS=Homo sapiens GN=CTSG PE=1 SV=2 - [CATG_HUMAN]	31,30	31,76	1	6	6	8	6,812E8	255	28,8	11,19
P68431	Histone H3.1 OS=Homo sapiens GN=HIST1H3A PE=1 SV=2 - [H31_HUMAN]	29,18	40,44	3	1	4	10	1,234E9	136	15,4	11,12
P68032	Actin, alpha cardiac muscle 1 OS=Homo sapiens GN=ACTC1 PE=1 SV=1 - [ACTC_HUMAN]	28,57	25,73	7	1	10	14	1,577E9	377	42,0	5,39
P69905	Hemoglobin subunit alpha OS=Homo sapiens GN=HBA1 PE=1 SV=2 - [HBA_HUMAN]	27,38	47,18	2	4	4	7	6,824E8	142	15,2	8,68
P08246	Neutrophil elastase OS=Homo sapiens GN=ELANE PE=1 SV=1 - [ELNE_HUMAN]	27,07	30,71	1	4	4	6	1,785E9	267	28,5	9,35
P12814	Alpha-actinin-1 OS=Homo sapiens GN=ACTN1 PE=1 SV=2 - [ACTN1_HUMAN]	27,06	11,21	4	9	9	10	2,313E8	892	103,0	5,41
P04083	Annexin A1 OS=Homo sapiens GN=ANXA1 PE=1 SV=2 - [ANXA1_HUMAN]	25,70	27,17	1	8	8	9	2,368E8	346	38,7	7,02
P12429	Annexin A3 OS=Homo sapiens GN=ANXA3 PE=1 SV=3 - [ANXA3_HUMAN]	24,62	27,55	1	8	8	9	1,992E8	323	36,4	5,92
Q71DI3	Histone H3.2 OS=Homo sapiens GN=HIST2H3A PE=1 SV=3 - [H32_HUMAN]	22,48	40,44	3	1	4	9	1,234E9	136	15,4	11,27
P04040	Catalase OS=Homo sapiens GN=CAT PE=1 SV=3 - [CATA_HUMAN]	21,84	16,70	1	6	6	6	1,161E8	527	59,7	7,39
P84243	Histone H3.3 OS=Homo sapiens GN=H3F3A PE=1 SV=2 - [H33_HUMAN]	20,71	40,44	3	1	4	9	1,234E9	136	15,3	11,27
P20160	Azurocidin OS=Homo sapiens GN=AZU1 PE=1 SV=3 - [CAP7_HUMAN]	19,64	20,32	1	3	3	5	7,768E8	251	26,9	9,50
P35579	Myosin-9 OS=Homo sapiens GN=MYH9 PE=1 SV=4 - [MYH9_HUMAN]	19,50	4,34	4	8	8	9	9,581E7	1960	226,4	5,60
P06744	Glucose-6-phosphate isomerase OS=Homo sapiens GN=GPI PE=1 SV=4 - [G6PI_HUMAN]	18,37	16,31	1	7	7	8	2,316E8	558	63,1	8,32
P00558	Phosphoglycerate kinase 1 OS=Homo sapiens GN=PGK1 PE=1 SV=3 - [PGK1_HUMAN]	17,76	11,27	1	3	3	5	1,901E8	417	44,6	8,10
P30740	Leukocyte elastase inhibitor OS=Homo sapiens GN=SERPINB1 PE=1 SV=1 - [ILEU_HUMAN]	17,23	20,84	1	7	7	7	1,929E8	379	42,7	6,28
P80188	Neutrophil gelatinase-associated lipocalin OS=Homo sapiens GN=LCN2 PE=1 SV=2 - [NGAL_HUMAN]	17,18	40,40	1	6	6	6	4,323E8	198	22,6	8,91
P61626	Lysozyme C OS=Homo sapiens GN=LYZ PE=1 SV=1 - [LYSC_HUMAN]	15,90	35,81	1	4	4	4	3,760E8	148	16,5	9,16
P14618	Pyruvate kinase PKM OS=Homo sapiens GN=PKM PE=1 SV=4 - [KPYM_HUMAN]	15,43	12,05	1	5	5	5	1,801E8	531	57,9	7,84
P52209	6-phosphogluconate dehydrogenase, decarboxylating OS=Homo sapiens GN=PGD PE=1 SV=3 - [6PGD_HUMAN]	15,03	13,87	1	5	5	5	1,088E8	483	53,1	7,23
P06396	Gelsolin OS=Homo sapiens GN=GSN PE=1 SV=1 - [GELS_HUMAN]	13,70	5,50	1	3	3	4	1,568E8	782	85,6	6,28

P04075	Fructose-bisphosphate aldolase A OS=Homo sapiens GN=ALDOA PE=1 SV=2 - [ALDOA_HUMAN]	11,22	14,29	1	3	3	3	9,307E7	364	39,4	8,09
P07737	Profilin-1 OS=Homo sapiens GN=PFN1 PE=1 SV=2 - [PROF1_HUMAN]	11,21	45,71	1	5	5	5	4,474E8	140	15,0	8,27
P50395	Rab GDP dissociation inhibitor beta OS=Homo sapiens GN=GDI2 PE=1 SV=2 - [GDIB_HUMAN]	11,13	8,54	2	2	2	3	4,720E7	445	50,6	6,47
P09211	Glutathione S-transferase P OS=Homo sapiens GN=GSTP1 PE=1 SV=2 - [GSTP1_HUMAN]	11,04	27,14	1	4	4	4	1,649E8	210	23,3	5,64
P08670	Vimentin OS=Homo sapiens GN=VIM PE=1 SV=4 - [VIME_HUMAN]	10,27	10,73	1	5	5	5	1,463E8	466	53,6	5,12
P41218	Myeloid cell nuclear differentiation antigen OS=Homo sapiens GN=MNDA PE=1 SV=1 - [MNDA_HUMAN]	10,26	11,79	1	3	3	4	7,414E7	407	45,8	9,76
Q01518	Adenylyl cyclase-associated protein 1 OS=Homo sapiens GN=CAP1 PE=1 SV=5 - [CAP1_HUMAN]	9,54	9,89	1	3	3	3	1,113E8	475	51,9	8,06
P63104	14-3-3 protein zeta/delta OS=Homo sapiens GN=YWHAZ PE=1 SV=1 - [1433Z_HUMAN]	9,43	21,63	6	3	4	4	1,134E8	245	27,7	4,79
P80511	Protein S100-A12 OS=Homo sapiens GN=S100A12 PE=1 SV=2 - [S10AC_HUMAN]	9,06	19,57	1	1	1	2	3,132E8	92	10,6	6,25
P12724	Eosinophil cationic protein OS=Homo sapiens GN=RNASE3 PE=1 SV=2 - [ECP_HUMAN]	8,56	22,50	1	3	3	3	1,165E8	160	18,4	10,02
P00338	L-lactate dehydrogenase A chain OS=Homo sapiens GN=LDHA PE=1 SV=2 - [LDHA_HUMAN]	8,55	11,14	4	3	3	3	2,629E8	332	36,7	8,27
P52790	Hexokinase-3 OS=Homo sapiens GN=HK3 PE=1 SV=2 - [HXK3_HUMAN]	8,41	2,06	1	1	1	2	3,322E7	923	99,0	5,40
P31946	14-3-3 protein beta/alpha OS=Homo sapiens GN=YWHAB PE=1 SV=3 - [1433B_HUMAN]	8,02	8,94	6	1	2	3	1,083E8	246	28,1	4,83
P59998	Actin-related protein 2/3 complex subunit 4 OS=Homo sapiens GN=ARPC4 PE=1 SV=3 - [ARPC4_HUMAN]	8,02	11,31	1	2	2	3	1,114E8	168	19,7	8,43
P06733	Alpha-enolase OS=Homo sapiens GN=ENO1 PE=1 SV=2 - [ENOA_HUMAN]	7,34	7,37	2	2	2	2	1,573E8	434	47,1	7,39
P24158	Myeloblastin OS=Homo sapiens GN=PRTN3 PE=1 SV=3 - [PRTN3_HUMAN]	7,13	11,33	1	3	3	3	4,608E8	256	27,8	8,35
O75594	Peptidoglycan recognition protein 1 OS=Homo sapiens GN=PGLYRP1 PE=1 SV=1 - [PGRP1_HUMAN]	6,93	15,82	1	2	2	2	8,724E7	196	21,7	8,59
P61158	Actin-related protein 3 OS=Homo sapiens GN=ACTR3 PE=1 SV=3 - [ARP3_HUMAN]	6,01	5,50	3	2	2	2	4,677E7	418	47,3	5,88
P60660	Myosin light polypeptide 6 OS=Homo sapiens GN=MYL6 PE=1 SV=2 - [MYL6_HUMAN]	5,93	19,21	2	2	2	2	1,055E8	151	16,9	4,65
P49913	Cathelicidin antimicrobial peptide OS=Homo sapiens GN=CAMP PE=1 SV=1 - [CAMP_HUMAN]	5,43	7,65	1	1	1	2	1,361E8	170	19,3	9,41
Q00610	Clathrin heavy chain 1 OS=Homo sapiens GN=CLTC PE=1 SV=5 - [CLH1_HUMAN]	5,18	1,25	2	2	2	2	4,836E7	1675	191,5	5,69
P08758	Annexin A5 OS=Homo sapiens GN=ANXA5 PE=1 SV=2 - [ANXA5_HUMAN]	5,15	5,00	1	1	1	1	5,862E7	320	35,9	5,05
P15259	Phosphoglycerate mutase 2 OS=Homo sapiens GN=PGAM2 PE=1 SV=3 - [PGAM2_HUMAN]	5,03	9,88	3	2	2	2	2,033E8	253	28,7	8,88
P04264	Keratin, type II cytoskeletal 1 OS=Homo sapiens GN=KRT1 PE=1 SV=6 - [K2C1_HUMAN]	4,98	3,42	8	2	2	2	9,079E7	644	66,0	8,12
P01023	Alpha-2-macroglobulin OS=Homo sapiens GN=A2M PE=1 SV=3 - [A2MG_HUMAN]	4,78	2,92	1	2	4	4	3,193E8	1474	163,2	6,46
P0DMV9	Heat shock 70 kDa protein 1B OS=Homo sapiens GN=HSPA1B PE=1 SV=1 - [HS71B_HUMAN]	4,75	3,12	7	2	2	2	6,904E7	641	70,0	5,66
P08133	Annexin A6 OS=Homo sapiens GN=ANXA6 PE=1 SV=3 - [ANXA6_HUMAN]	4,33	2,97	1	2	2	2	5,892E7	673	75,8	5,60
P11413	Glucose-6-phosphate 1-dehydrogenase OS=Homo sapiens GN=G6PD PE=1 SV=4 - [G6PD_HUMAN]	4,28	4,47	1	2	2	2	9,030E7	515	59,2	6,84
P28676	Grancalcin OS=Homo sapiens GN=GCA PE=1 SV=2 - [GRAN_HUMAN]	4,09	12,90	1	3	3	3	1,134E8	217	24,0	5,21
P60174	Triosephosphate isomerase OS=Homo sapiens GN=TPI1 PE=1 SV=3 - [TPIS_HUMAN]	3,86	4,20	1	1	1	1	1,141E8	286	30,8	5,92
P31146	Coronin-1A OS=Homo sapiens GN=CORO1A PE=1 SV=4 - [COR1A_HUMAN]	3,71	3,47	1	1	1	1	1,014E8	461	51,0	6,68
P02765	Alpha-2-HS-glycoprotein OS=Homo sapiens GN=AHSG PE=1 SV=1 - [FETUA_HUMAN]	3,64	3,27	1	1	1	1	1,717E8	367	39,3	5,72
Q9Y536	Peptidyl-prolyl cis-trans isomerase A-like 4A OS=Homo sapiens GN=PPIAL4A PE=2 SV=1 - [PAL4A_HUMAN]	3,42	8,54	2	1	1	1	2,853E8	164	18,2	9,22
Q8WVE0	EEF1A lysine methyltransferase 1 OS=Homo sapiens GN=EEF1AKMT1 PE=1 SV=1 - [EFMT1_HUMAN]	3,33	5,61	1	1	1	7	6,233E7	214	24,5	4,55
P05089	Arginase-1 OS=Homo sapiens GN=ARG1 PE=1 SV=2 - [ARGI1_HUMAN]	3,32	3,73	1	1	1	1	4,761E7	322	34,7	7,21
P25815	Protein S100-P OS=Homo sapiens GN=S100P PE=1 SV=2 - [S100P_HUMAN]	3,31	13,68	1	1	1	1	2,310E8	95	10,4	4,88
P22894	Neutrophil collagenase OS=Homo sapiens GN=MMP8 PE=1 SV=1 - [MMP8_HUMAN]	3,25	3,85	1	1	1	1		467	53,4	6,87

P23528	Cofilin-1 OS=Homo sapiens GN=CFL1 PE=1 SV=3 - [COF1_HUMAN]	3,20	10,24	2	1	1	1	5,562E7	166	18,5	8,09
P37837	Transaldolase OS=Homo sapiens GN=TALDO1 PE=1 SV=2 - [TALDO_HUMAN]	3,07	3,86	1	1	1	1	2,337E8	337	37,5	6,81
O15511	Actin-related protein 2/3 complex subunit 5 OS=Homo sapiens GN=ARPC5 PE=1 SV=3 - [ARPC5_HUMAN]	2,86	7,95	1	1	1	1	1,072E8	151	16,3	5,67
P31949	Protein S100-A11 OS=Homo sapiens GN=S100A11 PE=1 SV=2 - [S10AB_HUMAN]	2,85	15,24	1	1	1	1	1,004E8	105	11,7	7,12
P59665	Neutrophil defensin 1 OS=Homo sapiens GN=DEFA1 PE=1 SV=1 - [DEF1_HUMAN]	2,77	9,57	2	1	1	1	1,989E8	94	10,2	6,99
P46976	Glycogenin-1 OS=Homo sapiens GN=GYG1 PE=1 SV=4 - [GLYG_HUMAN]	2,57	2,86	1	1	1	1	8,433E7	350	39,4	5,53
O15143	Actin-related protein 2/3 complex subunit 1B OS=Homo sapiens GN=ARPC1B PE=1 SV=3 - [ARC1B_HUMAN]	2,52	2,69	1	1	1	1	5,623E7	372	40,9	8,35
P20742	Pregnancy zone protein OS=Homo sapiens GN=PZP PE=1 SV=4 - [PZP_HUMAN]	2,31	2,29	1	1	3	3	2,399E8	1482	163,8	6,38
P00491	Purine nucleoside phosphorylase OS=Homo sapiens GN=PNP PE=1 SV=2 - [PNPH_HUMAN]	2,29	3,46	1	1	1	1	9,747E7	289	32,1	6,95
P14780	Matrix metalloproteinase-9 OS=Homo sapiens GN=MMP9 PE=1 SV=3 - [MMP9_HUMAN]	2,21	2,55	1	2	2	2	7,733E7	707	78,4	6,06
O75367	Core histone macro-H2A.1 OS=Homo sapiens GN=H2AFY PE=1 SV=4 - [H2AY_HUMAN]	2,20	2,42	2	1	1	1	7,296E7	372	39,6	9,79
P26038	Moesin OS=Homo sapiens GN=MSN PE=1 SV=3 - [MOES_HUMAN]	2,16	1,56	3	1	1	1	1,333E8	577	67,8	6,40
P52566	Rho GDP-dissociation inhibitor 2 OS=Homo sapiens GN=ARHGDIB PE=1 SV=3 - [GDIR2_HUMAN]	2,14	12,94	1	1	1	1	4,280E7	201	23,0	5,21
P06703	Protein S100-A6 OS=Homo sapiens GN=S100A6 PE=1 SV=1 - [S10A6_HUMAN]	2,04	8,89	1	1	1	1	1,456E8	90	10,2	5,48
P11217	Glycogen phosphorylase, muscle form OS=Homo sapiens GN=PYGM PE=1 SV=6 - [PYGM_HUMAN]	2,02	0,95	3	1	1	1	5,674E7	842	97,0	7,03
P01008	Antithrombin-III OS=Homo sapiens GN=SERPINC1 PE=1 SV=1 - [ANT3_HUMAN]	1,86	3,45	1	2	2	2	5,054E7	464	52,6	6,71
O75131	Copine-3 OS=Homo sapiens GN=CPNE3 PE=1 SV=1 - [CPNE3_HUMAN]	1,78	1,68	8	1	1	1	5,118E7	537	60,1	5,85
Q8NG11	Tetraspanin-14 OS=Homo sapiens GN=TSPAN14 PE=1 SV=1 - [TSN14_HUMAN]	1,77	2,96	1	1	1	1	2,647E9	270	30,7	6,84
P05543	Thyroxine-binding globulin OS=Homo sapiens GN=SERPINA7 PE=1 SV=2 - [THBG_HUMAN]	1,75	2,41	1	1	1	1	4,980E7	415	46,3	6,30
P01024	Complement C3 OS=Homo sapiens GN=C3 PE=1 SV=2 - [CO3_HUMAN]	1,71	0,42	1	1	1	1	6,278E7	1663	187,0	6,40
Q9P225	Dynein heavy chain 2, axonemal OS=Homo sapiens GN=DNAH2 PE=2 SV=3 - [DYH2_HUMAN]	1,63	0,20	1	1	1	1	7,585E7	4427	507,4	6,37
Q6ZRS2	Helicase SRCAP OS=Homo sapiens GN=SRCAP PE=1 SV=3 - [SRCAP_HUMAN]	1,62	0,25	1	1	1	1	8,869E7	3230	343,3	5,96
Q9UKU7	Isobutyryl-CoA dehydrogenase, mitochondrial OS=Homo sapiens GN=ACAD8 PE=1 SV=1 - [ACAD8_HUMAN]	0,00	2,17	1	1	1	1		415	45,0	7,85
Q92667	A-kinase anchor protein 1, mitochondrial OS=Homo sapiens GN=AKAP1 PE=1 SV=1 - [AKAP1_HUMAN]	0,00	1,00	1	1	1	1	3,359E7	903	97,3	4,94
Q86WK7	Amphoterin-induced protein 3 OS=Homo sapiens GN=AMIGO3 PE=2 SV=1 - [AMGO3_HUMAN]	0,00	2,38	1	1	1	8	3,191E6	504	55,2	7,87
A6NKF2	AT-rich interactive domain-containing protein 3C OS=Homo sapiens GN=ARID3C PE=3 SV=1 - [ARI3C_HUMAN]	0,00	1,46	1	1	1	1		412	44,0	5,41
Q2TB18	Protein asteroid homolog 1 OS=Homo sapiens GN=ASTE1 PE=1 SV=1 - [ASTE1_HUMAN]	0,00	1,33	1	1	1	1	1,099E7	679	77,0	8,06
P51861	Cerebellar degeneration-related antigen 1 OS=Homo sapiens GN=CDR1 PE=1 SV=2 - [CDR1_HUMAN]	0,00	2,67	1	1	1	1	3,624E7	262	31,3	4,54
P29973	cGMP-gated cation channel alpha-1 OS=Homo sapiens GN=CNGA1 PE=1 SV=3 - [CNGA1_HUMAN]	0,00	3,33	1	1	1	1	2,828E7	690	79,5	7,81
P12109	Collagen alpha-1(VI) chain OS=Homo sapiens GN=COL6A1 PE=1 SV=3 - [CO6A1_HUMAN]	0,00	2,33	1	1	1	1	1,196E7	1028	108,5	5,43
A2RUB1	Meiosis-specific coiled-coil domain-containing protein MEIOC OS=Homo sapiens GN=MEIOC PE=2 SV=3 - [MEIOC_HUMAN]	0,00	1,89	1	1	1	1	2,224E7	952	107,5	7,12
P02771	Alpha-fetoprotein OS=Homo sapiens GN=AFP PE=1 SV=1 - [FETA_HUMAN]	0,00	1,64	1	1	1	1	4,050E8	609	68,6	5,68
P11488	Guanine nucleotide-binding protein G(t) subunit alpha-1 OS=Homo sapiens GN=GNAT1 PE=1 SV=5 - [GNAT1_HUMAN]	0,00	3,14	12	1	1	1	3,709E7	350	40,0	5,62
A7E2F4	Golgin subfamily A member 8A OS=Homo sapiens GN=GOLGA8A PE=2 SV=3 - [GOG8A_HUMAN]	0,00	1,74	1	1	1	1	5,831E7	631	70,1	6,24
Q8N8K9	Uncharacterized protein KIAA1958 OS=Homo sapiens GN=KIAA1958 PE=1 SV=1 - [K1958_HUMAN]	0,00	0,98	1	1	1	105		716	79,2	6,83
Q05315	Galectin-10 OS=Homo sapiens GN=CLC PE=1 SV=3 - [LEG10_HUMAN]	0,00	9,15	1	2	2	2	7,351E7	142	16,4	7,37
P11226	Mannose-binding protein C OS=Homo sapiens GN=MBL2 PE=1 SV=2 - [MBL2_HUMAN]	0,00	5,24	1	1	1	8	1,375E7	248	26,1	5,49

Q9NRN9	Methyltransferase-like protein 5 OS=Homo sapiens GN=METTL5 PE=1 SV=1 - [METL5_HUMAN]	0,00	4,78	1	1	1	1	3,665E7	209	23,7	6,68
Q9UJH8	Meteorin OS=Homo sapiens GN=METRN PE=2 SV=2 - [METRN_HUMAN]	0,00	4,78	1	1	1	1	1,665E8	293	31,2	8,09
Q13459	Unconventional myosin-IXb OS=Homo sapiens GN=MYO9B PE=1 SV=3 - [MYO9B_HUMAN]	0,00	0,60	1	1	1	1	4,030E6	2157	243,2	8,75
E9PQ53	NADH dehydrogenase [ubiquinone] 1 subunit C2, isoform 2 OS=Homo sapiens GN=NDUFC2-KCTD14 PE=1 SV=1 - [NDUCR	0,00	5,26	2	1	1	1	2,350E7	114	13,4	8,29
O15460	Prolyl 4-hydroxylase subunit alpha-2 OS=Homo sapiens GN=P4HA2 PE=1 SV=1 - [P4HA2_HUMAN]	0,00	2,24	1	1	1	2		535	60,9	5,71
Q9Y5I2	Protocadherin alpha-10 OS=Homo sapiens GN=PCDHA10 PE=2 SV=1 - [PCDAA_HUMAN]	0,00	1,58	2	1	1	1	3,307E8	948	102,8	5,17
Q9H6A9	Pecanex-like protein 3 OS=Homo sapiens GN=PCNX3 PE=1 SV=2 - [PCX3_HUMAN]	0,00	1,08	1	1	1	1	7,531E6	2034	221,9	6,64
P07237	Protein disulfide-isomerase OS=Homo sapiens GN=P4HB PE=1 SV=3 - [PDIA1_HUMAN]	0,00	1,57	1	1	1	1	2,997E7	508	57,1	4,87
P35354	Prostaglandin G/H synthase 2 OS=Homo sapiens GN=PTGS2 PE=1 SV=2 - [PGH2_HUMAN]	0,00	2,98	1	1	1	1	3,565E6	604	69,0	7,39
Q5H9R7	Serine/threonine-protein phosphatase 6 regulatory subunit 3 OS=Homo sapiens GN=PPP6R3 PE=1 SV=2 - [PP6R3_HUMAN]	0,00	1,49	2	1	1	1		873	97,6	4,60
Q96EY7	Pentatricopeptide repeat domain-containing protein 3, mitochondrial OS=Homo sapiens GN=PTCD3 PE=1 SV=3 - [PTCD3_H	0,00	1,45	1	1	1	1	5,524E7	689	78,5	6,42
Q14679	Tubulin polyglutamylase TTLL4 OS=Homo sapiens GN=TTLL4 PE=1 SV=2 - [TTLL4_HUMAN]	0,00	0,50	1	1	1	1		1199	133,3	8,85
O95155	Ubiquitin conjugation factor E4 B OS=Homo sapiens GN=UBE4B PE=1 SV=1 - [UBE4B_HUMAN]	0,00	0,69	1	1	1	1	5,140E7	1302	146,1	6,55
P25311	Zinc-alpha-2-glycoprotein OS=Homo sapiens GN=AZGP1 PE=1 SV=2 - [ZA2G_HUMAN]	0,00	4,03	1	1	1	1		298	34,2	6,05
P52747	Zinc finger protein 143 OS=Homo sapiens GN=ZNF143 PE=1 SV=2 - [ZN143_HUMAN]	0,00	2,66	1	1	1	2		638	68,9	6,05
Q8WTR7	Zinc finger protein 473 OS=Homo sapiens GN=ZNF473 PE=1 SV=1 - [ZN473_HUMAN]	0,00	2,18	1	1	1	1	1,446E7	871	100,1	8,27

Supplemental Table S3

Accession	Description	Score	Coverage	# Proteins	# Unique Peptides	# Peptides	# PSMs	Area	# AAs	MW [kDa]	calc. pI
P02768	Serum albumin OS=Homo sapiens GN=ALB PE=1 SV=2 - [ALBU_HUMAN]	229,65	8,21	1	6	6	126	5,229E9	609	69,3	6,28
P02788	Lactotransferrin OS=Homo sapiens GN=LTF PE=1 SV=6 - [TRFL_HUMAN]	91,67	40,14	1	24	24	29	1,227E9	710	78,1	8,12
P60709	Actin, cytoplasmic 1 OS=Homo sapiens GN=ACTB PE=1 SV=1 - [ACTB_HUMAN]	64,95	48,53	8	7	13	17	1,294E9	375	41,7	5,48
P68871	Hemoglobin subunit beta OS=Homo sapiens GN=HBB PE=1 SV=2 - [HBB_HUMAN]	34,50	71,43	4	4	9	11	1,577E9	147	16,0	7,28
P06702	Protein S100-A9 OS=Homo sapiens GN=S100A9 PE=1 SV=1 - [S10A9_HUMAN]	34,36	67,54	1	5	5	10	1,801E9	114	13,2	6,13
P69905	Hemoglobin subunit alpha OS=Homo sapiens GN=HBA1 PE=1 SV=2 - [HBA_HUMAN]	31,96	49,30	1	5	5	9	1,034E9	142	15,2	8,68
P04406	Glyceraldehyde-3-phosphate dehydrogenase OS=Homo sapiens GN=GAPDH PE=1 SV=3 - [G3P_HUMAN]	29,40	36,72	2	8	8	9	3,703E8	335	36,0	8,46
P05164	Myeloperoxidase OS=Homo sapiens GN=MPO PE=1 SV=1 - [PERM_HUMAN]	23,65	11,54	1	7	7	7	2,839E8	745	83,8	8,97
P63267	Actin, gamma-enteric smooth muscle OS=Homo sapiens GN=ACTG2 PE=1 SV=1 - [ACTH_HUMAN]	20,77	21,81	5	1	7	8	7,710E8	376	41,8	5,48
P02042	Hemoglobin subunit delta OS=Homo sapiens GN=HBD PE=1 SV=2 - [HBD_HUMAN]	20,29	41,50	4	1	6	7	1,555E9	147	16,0	8,05
P04264	Keratin, type II cytoskeletal 1 OS=Homo sapiens GN=KRT1 PE=1 SV=6 - [K2C1_HUMAN]	19,65	12,89	12	7	7	7	1,777E8	644	66,0	8,12
P08246	Neutrophil elastase OS=Homo sapiens GN=ELANE PE=1 SV=1 - [ELNE_HUMAN]	17,53	22,85	1	4	4	5	2,756E8	267	28,5	9,35
P62805	Histone H4 OS=Homo sapiens GN=HIST1H4A PE=1 SV=2 - [H4_HUMAN]	16,58	40,78	1	4	4	6	6,401E8	103	11,4	11,36
P05109	Protein S100-A8 OS=Homo sapiens GN=S100A8 PE=1 SV=1 - [S10A8_HUMAN]	15,76	24,73	1	3	3	6	2,289E9	93	10,8	7,03
P13796	Plastin-2 OS=Homo sapiens GN=LCP1 PE=1 SV=6 - [PLSL_HUMAN]	14,40	8,61	3	5	5	5	9,511E7	627	70,2	5,43
P01023	Alpha-2-macroglobulin OS=Homo sapiens GN=A2M PE=1 SV=3 - [A2MG_HUMAN]	12,74	3,46	1	3	5	6	5,069E8	1474	163,2	6,46
P68431	Histone H3.1 OS=Homo sapiens GN=HIST1H3A PE=1 SV=2 - [H31_HUMAN]	12,14	35,29	4	1	3	4	1,622E8	136	15,4	11,12
P06744	Glucose-6-phosphate isomerase OS=Homo sapiens GN=GPI PE=1 SV=4 - [G6PI_HUMAN]	11,76	5,73	1	2	2	3	1,359E8	558	63,1	8,32
P14780	Matrix metalloproteinase-9 OS=Homo sapiens GN=MMP9 PE=1 SV=3 - [MMP9_HUMAN]	11,57	7,07	1	4	4	4	1,542E8	707	78,4	6,06
P80188	Neutrophil gelatinase-associated lipocalin OS=Homo sapiens GN=LCN2 PE=1 SV=2 - [NGAL_HUMAN]	9,62	23,74	1	3	3	3	3,172E8	198	22,6	8,91
P06396	Gelsolin OS=Homo sapiens GN=GSN PE=1 SV=1 - [GELS_HUMAN]	9,49	3,58	1	2	2	3	8,403E7	782	85,6	6,28
P31946	14-3-3 protein beta/alpha OS=Homo sapiens GN=YWHAB PE=1 SV=3 - [1433B_HUMAN]	8,85	9,76	6	1	2	3	5,775E7	246	28,1	4,83
O60814	Histone H2B type 1-K OS=Homo sapiens GN=HIST1H2BK PE=1 SV=3 - [H2B1K_HUMAN]	8,75	19,05	14	2	2	3	6,841E8	126	13,9	10,32
Q71DI3	Histone H3.2 OS=Homo sapiens GN=HIST2H3A PE=1 SV=3 - [H32_HUMAN]	8,54	35,29	4	1	3	3	1,551E8	136	15,4	11,27
P01024	Complement C3 OS=Homo sapiens GN=C3 PE=1 SV=2 - [CO3_HUMAN]	7,40	1,32	1	1	1	2	8,168E7	1663	187,0	6,40
P20742	Pregnancy zone protein OS=Homo sapiens GN=PZP PE=1 SV=4 - [PZP_HUMAN]	6,80	2,29	1	1	3	3	3,304E8	1482	163,8	6,38
P13645	Keratin, type I cytoskeletal 10 OS=Homo sapiens GN=KRT10 PE=1 SV=6 - [K1C10_HUMAN]	6,35	3,94	1	1	1	1	9,562E7	584	58,8	5,21
P12814	Alpha-actinin-1 OS=Homo sapiens GN=ACTN1 PE=1 SV=2 - [ACTN1_HUMAN]	6,26	4,15	3	3	3	3	7,179E7	892	103,0	5,41
P00558	Phosphoglycerate kinase 1 OS=Homo sapiens GN=PGK1 PE=1 SV=3 - [PGK1_HUMAN]	6,16	8,15	1	2	2	2	1,018E8	417	44,6	8,10
P35527	Keratin, type I cytoskeletal 9 OS=Homo sapiens GN=KRT9 PE=1 SV=3 - [K1C9_HUMAN]	6,10	3,69	1	1	1	1	2,863E7	623	62,0	5,24
P14618	Pyruvate kinase PKM OS=Homo sapiens GN=PKM PE=1 SV=4 - [KPYM_HUMAN]	6,10	4,52	1	2	2	2	7,968E7	531	57,9	7,84
P04083	Annexin A1 OS=Homo sapiens GN=ANXA1 PE=1 SV=2 - [ANXA1_HUMAN]	6,04	8,96	1	2	2	2	1,206E8	346	38,7	7,02
P49913	Cathelicidin antimicrobial peptide OS=Homo sapiens GN=CAMP PE=1 SV=1 - [CAMP_HUMAN]	5,92	12,94	1	2	2	2	9,576E7	170	19,3	9,41
P29401	Transketolase OS=Homo sapiens GN=TKT PE=1 SV=3 - [TKT_HUMAN]	5,80	3,85	1	2	2	2	1,054E8	623	67,8	7,66
P19823	Inter-alpha-trypsin inhibitor heavy chain H2 OS=Homo sapiens GN=ITIH2 PE=1 SV=2 - [ITIH2_HUMAN]	5,66	1,90	1	2	2	3	1,473E8	946	106,4	6,86

P00338	L-lactate dehydrogenase A chain OS=Homo sapiens GN=LDHA PE=1 SV=2 - [LDHA_HUMAN]	5,36	9,34	4	3	3	3	1,287E8	332	36,7	8,27
P08311	Cathepsin G OS=Homo sapiens GN=CTSG PE=1 SV=2 - [CATG_HUMAN]	5,15	8,63	1	2	2	2	8,664E7	255	28,8	11,19
P06733	Alpha-enolase OS=Homo sapiens GN=ENO1 PE=1 SV=2 - [ENOA_HUMAN]	5,10	6,68	1	2	2	2	1,005E8	434	47,1	7,39
P24158	Myeloblastin OS=Homo sapiens GN=PRTN3 PE=1 SV=3 - [PRTN3_HUMAN]	5,04	8,20	1	2	2	2	1,879E8	256	27,8	8,35
Q96KK5	Histone H2A type 1-H OS=Homo sapiens GN=HIST1H2AH PE=1 SV=3 - [H2A1H_HUMAN]	4,78	21,88	15	2	2	2	4,056E8	128	13,9	10,89
P22894	Neutrophil collagenase OS=Homo sapiens GN=MMP8 PE=1 SV=1 - [MMP8_HUMAN]	4,57	3,43	1	1	1	1	8,390E7	467	53,4	6,87
P63104	14-3-3 protein zeta/delta OS=Homo sapiens GN=YWHAZ PE=1 SV=1 - [1433Z_HUMAN]	4,50	8,98	6	1	2	2	5,899E7	245	27,7	4,79
P20160	Azurocidin OS=Homo sapiens GN=AZU1 PE=1 SV=3 - [CAP7_HUMAN]	3,66	5,18	1	1	1	1	1,082E8	251	26,9	9,50
Q8WVE0	EEF1A lysine methyltransferase 1 OS=Homo sapiens GN=EEF1AKMT1 PE=1 SV=1 - [EFMT1_HUMAN]	3,32	5,61	1	1	1	15	3,570E8	214	24,5	4,55
O75594	Peptidoglycan recognition protein 1 OS=Homo sapiens GN=PGLYRP1 PE=1 SV=1 - [PGRP1_HUMAN]	3,27	7,65	1	1	1	1	6,271E7	196	21,7	8,59
P02765	Alpha-2-HS-glycoprotein OS=Homo sapiens GN=AHSG PE=1 SV=1 - [FETUA_HUMAN]	3,25	3,27	1	1	1	1	1,858E8	367	39,3	5,72
P02647	Apolipoprotein A-I OS=Homo sapiens GN=APOA1 PE=1 SV=1 - [APOA1_HUMAN]	3,09	5,99	1	1	1	1	2,143E8	267	30,8	5,76
P35579	Myosin-9 OS=Homo sapiens GN=MYH9 PE=1 SV=4 - [MYH9_HUMAN]	2,88	0,77	2	1	1	1	5,995E6	1960	226,4	5,60
P02771	Alpha-fetoprotein OS=Homo sapiens GN=AFP PE=1 SV=1 - [FETA_HUMAN]	2,84	2,63	1	1	1	1	1,075E8	609	68,6	5,68
P37837	Transaldolase OS=Homo sapiens GN=TALDO1 PE=1 SV=2 - [TALDO_HUMAN]	2,75	3,86	1	1	1	1	8,378E7	337	37,5	6,81
Q9C0K3	Actin-related protein 3C OS=Homo sapiens GN=ACTR3C PE=2 SV=1 - [ARP3C_HUMAN]	2,72	5,24	3	1	1	1	1,622E7	210	23,7	5,58
P59665	Neutrophil defensin 1 OS=Homo sapiens GN=DEFA1 PE=1 SV=1 - [DEF1_HUMAN]	2,49	9,57	2	1	1	1	2,221E8	94	10,2	6,99
Q01518	Adenylyl cyclase-associated protein 1 OS=Homo sapiens GN=CAP1 PE=1 SV=5 - [CAP1_HUMAN]	2,48	2,74	1	1	1	1	4,301E7	475	51,9	8,06
P26038	Moesin OS=Homo sapiens GN=MSN PE=1 SV=3 - [MOES_HUMAN]	2,45	2,08	3	1	1	1	7,087E6	577	67,8	6,40
P07737	Profilin-1 OS=Homo sapiens GN=PFN1 PE=1 SV=2 - [PROF1_HUMAN]	2,45	17,14	1	2	2	2	1,141E8	140	15,0	8,27
P30740	Leukocyte elastase inhibitor OS=Homo sapiens GN=SERPINB1 PE=1 SV=1 - [ILEU_HUMAN]	2,10	2,64	1	1	1	1	1,459E7	379	42,7	6,28
P02774	Vitamin D-binding protein OS=Homo sapiens GN=GC PE=1 SV=1 - [VTDB_HUMAN]	1,98	1,69	1	1	1	1	1,236E7	474	52,9	5,54
Q9Y6V0	Protein piccolo OS=Homo sapiens GN=PCLO PE=1 SV=4 - [PCLO_HUMAN]	1,75	0,18	1	1	1	3	4,946E9	5065	552,9	6,51
P59998	Actin-related protein 2/3 complex subunit 4 OS=Homo sapiens GN=ARPC4 PE=1 SV=3 - [ARPC4_HUMAN]	1,70	4,76	1	1	1	1	3,435E7	168	19,7	8,43
Q6UWY0	Arylsulfatase K OS=Homo sapiens GN=ARSK PE=1 SV=1 - [ARSK_HUMAN]	1,61	1,31	1	1	1	1	4,305E7	536	61,4	8,92
Q8NG11	Tetraspanin-14 OS=Homo sapiens GN=TSPAN14 PE=1 SV=1 - [TSN14_HUMAN]	1,27	2,96	1	1	1	1		270	30,7	6,84
P01008	Antithrombin-III OS=Homo sapiens GN=SERPINC1 PE=1 SV=1 - [ANT3_HUMAN]	0,00	3,88	1	2	2	2	4,254E7	464	52,6	6,71
Q9BXJ4	Complement C1q tumor necrosis factor-related protein 3 OS=Homo sapiens GN=C1QTNF3 PE=1 SV=1 - [C1QT3_HUMAN]	0,00	7,72	1	1	1	1		246	27,0	6,52
P51861	Cerebellar degeneration-related antigen 1 OS=Homo sapiens GN=CDR1 PE=1 SV=2 - [CDR1_HUMAN]	0,00	2,67	1	1	1	1	5,639E7	262	31,3	4,54
Q9Y6F8	Testis-specific chromodomain protein Y 1 OS=Homo sapiens GN=CDY1 PE=1 SV=1 - [CDY1_HUMAN]	0,00	1,11	2	1	1	1	5,583E6	540	60,4	9,10
P31146	Coronin-1A OS=Homo sapiens GN=CORO1A PE=1 SV=4 - [COR1A_HUMAN]	0,00	2,17	1	1	1	1	3,111E7	461	51,0	6,68
Q9P225	Dynein heavy chain 2, axonemal OS=Homo sapiens GN=DNAH2 PE=2 SV=3 - [DYH2_HUMAN]	0,00	0,50	1	1	1	1		4427	507,4	6,37
A0FGR9	Extended synaptotagmin-3 OS=Homo sapiens GN=ESYT3 PE=1 SV=1 - [ESYT3_HUMAN]	0,00	0,79	1	1	1	1	8,058E7	886	100,0	8,37
Q9Y4F4	TOG array regulator of axonemal microtubules protein 1 OS=Homo sapiens GN=TOGARAM1 PE=1 SV=4 - [TGRM1_HUMAN]	0,00	0,52	1	1	1	1		1720	189,2	8,50
Q01415	N-acetylgalactosamine kinase OS=Homo sapiens GN=GALK2 PE=1 SV=1 - [GALK2_HUMAN]	0,00	2,62	1	1	1	1	3,712E7	458	50,3	6,61
P80217	Interferon-induced 35 kDa protein OS=Homo sapiens GN=IFI35 PE=1 SV=5 - [IN35_HUMAN]	0,00	12,59	1	1	1	1	1,608E7	286	31,5	6,09
Q6ZN16	Mitogen-activated protein kinase kinase kinase 15 OS=Homo sapiens GN=MAP3K15 PE=1 SV=2 - [M3K15_HUMAN]	0,00	0,61	1	1	1	1	6,622E7	1313	147,3	5,63

P43246	DNA mismatch repair protein Msh2 OS=Homo sapiens GN=MSH2 PE=1 SV=1 - [MSH2_HUMAN]	0,00	4,39	1	1	1	1		934	104,7	5,77
Q7RTR0	NACHT, LRR and PYD domains-containing protein 9 OS=Homo sapiens GN=NLRP9 PE=1 SV=1 - [NLRP9_HUMAN]	0,00	1,21	1	1	1	1		991	113,2	6,51
P37198	Nuclear pore glycoprotein p62 OS=Homo sapiens GN=NUP62 PE=1 SV=3 - [NUP62_HUMAN]	0,00	1,34	1	1	1	1		522	53,2	5,31
Q149M9	NACHT domain- and WD repeat-containing protein 1 OS=Homo sapiens GN=NWD1 PE=1 SV=3 - [NWD1_HUMAN]	0,00	0,51	1	1	1	2		1564	174,4	6,74
Q96D21	GTP-binding protein Rhes OS=Homo sapiens GN=RASD2 PE=1 SV=1 - [RHES_HUMAN]	0,00	4,89	1	1	1	7	5,258E7	266	30,3	8,90
Q9UGH3	Solute carrier family 23 member 2 OS=Homo sapiens GN=SLC23A2 PE=1 SV=1 - [S23A2_HUMAN]	0,00	4,15	1	1	1	1		650	70,3	7,68
Q9UQD0	Sodium channel protein type 8 subunit alpha OS=Homo sapiens GN=SCN8A PE=1 SV=1 - [SCN8A_HUMAN]	0,00	0,61	1	1	1	1	5,198E7	1980	225,1	6,28
Q5SQN1	Synaptosomal-associated protein 47 OS=Homo sapiens GN=SNAP47 PE=1 SV=3 - [SNP47_HUMAN]	0,00	2,80	1	1	1	1		464	52,5	8,69
Q8NBK3	Sulfatase-modifying factor 1 OS=Homo sapiens GN=SUMF1 PE=1 SV=3 - [SUMF1_HUMAN]	0,00	1,60	1	1	1	1	1,139E7	374	40,5	6,65
Q9NZQ8	Transient receptor potential cation channel subfamily M member 5 OS=Homo sapiens GN=TRPM5 PE=2 SV=1 - [TRPM5_HUMAN]	0,00	0,60	1	1	1	1		1165	131,4	6,77
Q9BS34	Zinc finger protein 670 OS=Homo sapiens GN=ZNF670 PE=1 SV=1 - [ZN670_HUMAN]	0,00	3,08	1	1	1	1	3,022E10	389	44,6	8,46

Supplemental Table S4

Accession	Description	Score	Coverage	# Proteins	# Unique Peptides	# Peptides	# PSMs	Area	# AAs	MW [kDa]	calc. pI
P02768	Serum albumin OS=Homo sapiens GN=ALB PE=1 SV=2 - [ALBU_HUMAN]	492,96	77,83	1	48	48	309	9,370E9	609	69,3	6,28
P0DOX5	Immunoglobulin gamma-1 heavy chain OS=Homo sapiens PE=1 SV=1 - [IGG1_HUMAN]	284,57	48,11	2	7	15	218	1,047E10	449	49,3	8,72
P01860	Immunoglobulin heavy constant gamma 3 OS=Homo sapiens GN=IGHG3 PE=1 SV=2 - [IGHG3_HUMAN]	135,50	50,93	1	6	13	98	1,013E10	377	41,3	7,90
P01859	Immunoglobulin heavy constant gamma 2 OS=Homo sapiens GN=IGHG2 PE=1 SV=2 - [IGHG2_HUMAN]	117,46	49,39	1	4	10	57	9,051E9	326	35,9	7,59
P01834	Immunoglobulin kappa constant OS=Homo sapiens GN=IGKC PE=1 SV=2 - [IGKC_HUMAN]	99,81	82,24	1	2	7	91	9,058E9	107	11,8	6,52
P0DOX7	Immunoglobulin kappa light chain OS=Homo sapiens PE=1 SV=1 - [IGK_HUMAN]	81,98	45,79	2	2	7	79	8,691E9	214	23,4	7,17
P60709	Actin, cytoplasmic 1 OS=Homo sapiens GN=ACTB PE=1 SV=1 - [ACTB_HUMAN]	57,24	50,40	8	6	13	17	6,273E8	375	41,7	5,48
P01861	Immunoglobulin heavy constant gamma 4 OS=Homo sapiens GN=IGHG4 PE=1 SV=1 - [IGHG4_HUMAN]	50,23	50,15	1	4	10	27	7,824E9	327	35,9	7,36
P02788	Lactotransferrin OS=Homo sapiens GN=LTF PE=1 SV=6 - [TRFL_HUMAN]	48,55	22,25	1	12	12	15	2,715E8	710	78,1	8,12
P06702	Protein S100-A9 OS=Homo sapiens GN=S100A9 PE=1 SV=1 - [S10A9_HUMAN]	41,06	67,54	1	5	5	13	4,825E8	114	13,2	6,13
P68871	Hemoglobin subunit beta OS=Homo sapiens GN=HBB PE=1 SV=2 - [HBB_HUMAN]	32,02	77,55	4	5	10	12	5,120E8	147	16,0	7,28
P0DOY2	Immunoglobulin lambda constant 2 OS=Homo sapiens GN=IGLC2 PE=1 SV=1 - [IGLC2_HUMAN]	25,50	74,53	4	2	5	10	3,835E9	106	11,3	7,24
P0CG04	Immunoglobulin lambda constant 1 OS=Homo sapiens GN=IGLC1 PE=1 SV=1 - [IGLC1_HUMAN]	22,63	65,09	3	1	4	8	3,395E9	106	11,3	7,87
P02042	Hemoglobin subunit delta OS=Homo sapiens GN=HBD PE=1 SV=2 - [HBD_HUMAN]	18,77	41,50	4	1	6	8	4,537E8	147	16,0	8,05
P68032	Actin, alpha cardiac muscle 1 OS=Homo sapiens GN=ACTC1 PE=1 SV=1 - [ACTC_HUMAN]	18,21	23,87	5	1	8	9	3,250E8	377	42,0	5,39
P04406	Glyceraldehyde-3-phosphate dehydrogenase OS=Homo sapiens GN=GAPDH PE=1 SV=3 - [G3P_HUMAN]	15,50	21,79	1	4	4	4	1,260E8	335	36,0	8,46
P00738	Haptoglobin OS=Homo sapiens GN=HP PE=1 SV=1 - [HPT_HUMAN]	13,83	15,27	2	5	5	5	8,808E7	406	45,2	6,58
P62805	Histone H4 OS=Homo sapiens GN=HIST1H4A PE=1 SV=2 - [H4_HUMAN]	13,50	40,78	1	4	4	5	1,640E8	103	11,4	11,36
P05109	Protein S100-A8 OS=Homo sapiens GN=S100A8 PE=1 SV=1 - [S10A8_HUMAN]	12,58	47,31	1	4	4	6	5,741E8	93	10,8	7,03
P69905	Hemoglobin subunit alpha OS=Homo sapiens GN=HBA1 PE=1 SV=2 - [HBA_HUMAN]	12,37	49,30	1	4	4	5	3,930E8	142	15,2	8,68
P13796	Plastin-2 OS=Homo sapiens GN=LCP1 PE=1 SV=6 - [PLSL_HUMAN]	9,63	9,89	3	4	4	4	5,022E7	627	70,2	5,43
P01717	Immunoglobulin lambda variable 3-25 OS=Homo sapiens GN=IGLV3-25 PE=1 SV=2 - [LV325_HUMAN]	8,24	18,75	4	2	2	3	5,986E7	112	12,0	4,50
P01599	Immunoglobulin kappa variable 1-17 OS=Homo sapiens GN=IGKV1-17 PE=1 SV=2 - [KV117_HUMAN]	7,92	13,68	1	1	1	2	2,891E8	117	12,8	8,68
O60814	Histone H2B type 1-K OS=Homo sapiens GN=HIST1H2BK PE=1 SV=3 - [H2B1K_HUMAN]	7,84	19,05	15	2	2	3	2,392E8	126	13,9	10,32
P01782	Immunoglobulin heavy variable 3-9 OS=Homo sapiens GN=IGHV3-9 PE=1 SV=2 - [HV309_HUMAN]	7,79	25,42	5	2	2	2	1,603E8	118	12,9	7,08
P05164	Myeloperoxidase OS=Homo sapiens GN=MPO PE=1 SV=1 - [PERM_HUMAN]	7,78	5,64	2	3	3	3	6,530E7	745	83,8	8,97
P04075	Fructose-bisphosphate aldolase A OS=Homo sapiens GN=ALDOA PE=1 SV=2 - [ALDOA_HUMAN]	7,61	8,79	1	2	2	3	4,644E7	364	39,4	8,09
P01619	Immunoglobulin kappa variable 3-20 OS=Homo sapiens GN=IGKV3-20 PE=1 SV=2 - [KV320_HUMAN]	7,27	21,55	1	1	2	2	3,537E8	116	12,5	4,96
A0A0C4DH25	Immunoglobulin kappa variable 3D-20 OS=Homo sapiens GN=IGKV3D-20 PE=3 SV=1 - [KVD20_HUMAN]	7,16	21,55	1	1	2	2	2,115E8	116	12,5	4,59
P06744	Glucose-6-phosphate isomerase OS=Homo sapiens GN=GPI PE=1 SV=4 - [G6PI_HUMAN]	6,98	5,73	1	2	2	2	4,661E7	558	63,1	8,32
A0A0C4DH42	Immunoglobulin heavy variable 3-66 OS=Homo sapiens GN=IGHV3-66 PE=3 SV=1 - [HV366_HUMAN]	6,81	18,97	9	2	2	2	3,369E8	116	12,7	8,16
P68104	Elongation factor 1-alpha 1 OS=Homo sapiens GN=EEF1A1 PE=1 SV=1 - [EF1A1_HUMAN]	6,23	8,66	3	2	2	2	7,324E7	462	50,1	9,01
P00338	L-lactate dehydrogenase A chain OS=Homo sapiens GN=LDHA PE=1 SV=2 - [LDHA_HUMAN]	5,93	6,63	4	2	2	2	6,884E7	332	36,7	8,27
P12814	Alpha-actinin-1 OS=Homo sapiens GN=ACTN1 PE=1 SV=2 - [ACTN1_HUMAN]	5,42	2,47	2	2	2	2	5,426E7	892	103,0	5,41
P08238	Heat shock protein HSP 90-beta OS=Homo sapiens GN=HSP90AB1 PE=1 SV=4 - [HS90B_HUMAN]	4,64	2,62	3	1	2	2	5,754E7	724	83,2	5,03

A0A075B6K5	Immunoglobulin lambda variable 3-9 OS=Homo sapiens GN=IGLV3-9 PE=3 SV=1 - [LV39_HUMAN]	4,62	13,91	2	1	1	1	1,927E8	115	12,3	7,39
P08246	Neutrophil elastase OS=Homo sapiens GN=ELANE PE=1 SV=1 - [ELNE_HUMAN]	4,58	3,75	1	1	1	2	5,170E7	267	28,5	9,35
A0A0C4DH72	Immunoglobulin kappa variable 1-6 OS=Homo sapiens GN=IGKV1-6 PE=3 SV=1 - [KV106_HUMAN]	4,35	13,68	4	1	1	1	1,682E8	117	12,7	8,29
P01871	Immunoglobulin heavy constant mu OS=Homo sapiens GN=IGHM PE=1 SV=4 - [IGHM_HUMAN]	4,01	6,84	2	2	2	2	5,440E7	453	49,4	6,77
P01593	Immunoglobulin kappa variable 1D-33 OS=Homo sapiens GN=IGKV1D-33 PE=1 SV=2 - [KVD33_HUMAN]	3,86	13,68	1	1	1	1	1,159E8	117	12,8	4,78
A0A0C4DH67	Immunoglobulin kappa variable 1-8 OS=Homo sapiens GN=IGKV1-8 PE=3 SV=1 - [KV108_HUMAN]	3,59	13,91	3	1	1	1	2,834E8	115	12,5	9,01
P02765	Alpha-2-HS-glycoprotein OS=Homo sapiens GN=AHSG PE=1 SV=1 - [FETUA_HUMAN]	3,56	3,27	1	1	1	1	7,448E7	367	39,3	5,72
P01700	Immunoglobulin lambda variable 1-47 OS=Homo sapiens GN=IGLV1-47 PE=1 SV=2 - [LV147_HUMAN]	3,54	11,11	1	1	1	1	1,041E8	117	12,3	5,91
P80188	Neutrophil gelatinase-associated lipocalin OS=Homo sapiens GN=LCN2 PE=1 SV=2 - [NGAL_HUMAN]	3,50	7,58	1	1	1	1	8,313E7	198	22,6	8,91
P14780	Matrix metalloproteinase-9 OS=Homo sapiens GN=MMP9 PE=1 SV=3 - [MMP9_HUMAN]	3,07	2,12	1	1	1	1	4,314E7	707	78,4	6,06
Q16513	Serine/threonine-protein kinase N2 OS=Homo sapiens GN=PKN2 PE=1 SV=1 - [PKN2_HUMAN]	2,96	2,95	1	1	1	1	5,776E7	984	112,0	6,30
P07900	Heat shock protein HSP 90-alpha OS=Homo sapiens GN=HSP90AA1 PE=1 SV=5 - [HS90A_HUMAN]	2,94	2,60	4	1	2	2	5,321E7	732	84,6	5,02
P02647	Apolipoprotein A-I OS=Homo sapiens GN=APOA1 PE=1 SV=1 - [APOA1_HUMAN]	2,84	5,99	1	1	1	1	6,116E7	267	30,8	5,76
P06312	Immunoglobulin kappa variable 4-1 OS=Homo sapiens GN=IGKV4-1 PE=1 SV=1 - [KV401_HUMAN]	2,68	7,44	1	1	1	1	1,772E8	121	13,4	5,25
A0A0A0MRZ8	Immunoglobulin kappa variable 3D-11 OS=Homo sapiens GN=IGKV3D-11 PE=3 SV=6 - [KVD11_HUMAN]	2,65	7,83	2	1	1	1	3,895E8	115	12,6	5,29
A0A075B6P5	Immunoglobulin kappa variable 2-28 OS=Homo sapiens GN=IGKV2-28 PE=3 SV=1 - [KV228_HUMAN]	2,59	10,83	7	1	1	1	2,786E8	120	12,9	5,94
P60174	Triosephosphate isomerase OS=Homo sapiens GN=TPI1 PE=1 SV=3 - [TPIS_HUMAN]	2,57	5,24	1	1	1	1	4,075E7	286	30,8	5,92
P04264	Keratin, type II cytoskeletal 1 OS=Homo sapiens GN=KRT1 PE=1 SV=6 - [K2C1_HUMAN]	2,56	1,86	1	1	1	1	3,848E7	644	66,0	8,12
P48741	Putative heat shock 70 kDa protein 7 OS=Homo sapiens GN=HSPA7 PE=5 SV=2 - [HSP77_HUMAN]	2,53	3,00	7	1	1	1	3,802E7	367	40,2	7,87
P06733	Alpha-enolase OS=Homo sapiens GN=ENO1 PE=1 SV=2 - [ENOA_HUMAN]	2,50	5,76	1	2	2	2	5,462E7	434	47,1	7,39
P59665	Neutrophil defensin 1 OS=Homo sapiens GN=DEFA1 PE=1 SV=1 - [DEF1_HUMAN]	2,48	9,57	2	1	1	1	5,445E7	94	10,2	6,99
A0A0A0MS15	Immunoglobulin heavy variable 3-49 OS=Homo sapiens GN=IGHV3-49 PE=3 SV=1 - [HV349_HUMAN]	2,44	7,56	1	1	1	1	8,333E7	119	13,0	8,62
P01023	Alpha-2-macroglobulin OS=Homo sapiens GN=A2M PE=1 SV=3 - [A2MG_HUMAN]	2,42	1,36	2	2	2	2	1,997E8	1474	163,2	6,46
P01624	Immunoglobulin kappa variable 3-15 OS=Homo sapiens GN=IGKV3-15 PE=1 SV=2 - [KV315_HUMAN]	2,37	7,83	2	1	1	1	4,531E8	115	12,5	5,19
A0A0C4DH34	Immunoglobulin heavy variable 4-28 OS=Homo sapiens GN=IGHV4-28 PE=3 SV=1 - [HV428_HUMAN]	2,35	7,69	1	1	1	1	1,842E7	117	13,1	9,29
P01714	Immunoglobulin lambda variable 3-19 OS=Homo sapiens GN=IGLV3-19 PE=1 SV=2 - [LV319_HUMAN]	2,30	8,04	1	1	1	1	2,623E7	112	12,0	4,96
P02771	Alpha-fetoprotein OS=Homo sapiens GN=AFP PE=1 SV=1 - [FETA_HUMAN]	2,30	4,27	1	2	2	2	8,025E7	609	68,6	5,68
P31946	14-3-3 protein beta/alpha OS=Homo sapiens GN=YWHAB PE=1 SV=3 - [1433B_HUMAN]	2,26	9,76	7	2	2	3	5,235E7	246	28,1	4,83
P01825	Immunoglobulin heavy variable 4-59 OS=Homo sapiens GN=IGHV4-59 PE=1 SV=2 - [HV459_HUMAN]	2,24	7,76	6	1	1	1	1,995E8	116	12,9	9,29
P49913	Cathelicidin antimicrobial peptide OS=Homo sapiens GN=CAMP PE=1 SV=1 - [CAMP_HUMAN]	2,21	5,29	1	1	1	1	2,355E7	170	19,3	9,41
Q66K66	Transmembrane protein 198 OS=Homo sapiens GN=TMEM198 PE=1 SV=1 - [TM198_HUMAN]	2,18	2,50	1	1	1	1	8,548E8	360	39,4	9,92
P14618	Pyruvate kinase PKM OS=Homo sapiens GN=PKM PE=1 SV=4 - [KPYM_HUMAN]	2,15	1,51	1	1	1	1	5,278E7	531	57,9	7,84
P26038	Moesin OS=Homo sapiens GN=MSN PE=1 SV=3 - [MOES_HUMAN]	2,13	1,56	3	1	1	1	7,015E7	577	67,8	6,40
Q9BQE3	Tubulin alpha-1C chain OS=Homo sapiens GN=TUBA1C PE=1 SV=1 - [TBA1C_HUMAN]	2,12	2,00	3	1	1	1	2,748E7	449	49,9	5,10
Q00610	Clathrin heavy chain 1 OS=Homo sapiens GN=CLTC PE=1 SV=5 - [CLH1_HUMAN]	2,10	0,54	1	1	1	1	1,622E7	1675	191,5	5,69
Q8NI35	InaD-like protein OS=Homo sapiens GN=PATJ PE=1 SV=3 - [INADL_HUMAN]	1,93	0,33	1	1	1	1	4,104E8	1801	196,2	4,94
A0A0B4J1V0	Immunoglobulin heavy variable 3-15 OS=Homo sapiens GN=IGHV3-15 PE=3 SV=1 - [HV315_HUMAN]	1,87	5,88	3	1	1	1	8,768E7	119	12,9	8,62

P07437	Tubulin beta chain OS=Homo sapiens GN=TUBB PE=1 SV=2 - [TBB5_HUMAN]	1,74	5,41	9	2	2	2	2,749E7	444	49,6	4,89
O60292	Signal-induced proliferation-associated 1-like protein 3 OS=Homo sapiens GN=SIPA1L3 PE=1 SV=3 - [SI1L3_HUMAN]	1,72	0,73	1	1	1	1	1,317E8	1781	194,5	8,32
A0A0B4J1U7	Immunoglobulin heavy variable 6-1 OS=Homo sapiens GN=IGHV6-1 PE=3 SV=1 - [HV601_HUMAN]	1,70	5,79	1	1	1	1		121	13,5	9,20
P02774	Vitamin D-binding protein OS=Homo sapiens GN=GC PE=1 SV=1 - [VTDB_HUMAN]	1,63	1,48	1	1	1	1	4,954E7	474	52,9	5,54
P27216	Annexin A13 OS=Homo sapiens GN=ANXA13 PE=1 SV=3 - [ANX13_HUMAN]	0,00	2,22	1	1	1	1	7,903E7	316	35,4	5,60
Q96G01	Protein bicaudal D homolog 1 OS=Homo sapiens GN=BICD1 PE=1 SV=3 - [BICD1_HUMAN]	0,00	1,13	1	1	1	1	2,570E7	975	110,7	5,81
Q8NG31	Kinetochore scaffold 1 OS=Homo sapiens GN=KNL1 PE=1 SV=3 - [KNL1_HUMAN]	0,00	0,51	1	1	1	1	3,888E6	2342	265,2	5,47
Q14839	Chromodomain-helicase-DNA-binding protein 4 OS=Homo sapiens GN=CHD4 PE=1 SV=2 - [CHD4_HUMAN]	0,00	0,89	1	1	1	1	1,117E8	1912	217,9	5,86
P0CG12	Chromosome transmission fidelity protein 8 homolog isoform 2 OS=Homo sapiens GN=CHTF8 PE=1 SV=1 - [CTF8A_HUMAN]	0,00	2,67	1	1	1	1	1,614E6	524	51,4	12,41
Q9H5Z1	Probable ATP-dependent RNA helicase DHX35 OS=Homo sapiens GN=DHX35 PE=1 SV=2 - [DHX35_HUMAN]	0,00	1,56	1	1	1	1	6,599E7	703	78,9	8,59
Q9Y6K1	DNA (cytosine-5)-methyltransferase 3A OS=Homo sapiens GN=DNMT3A PE=1 SV=4 - [DNM3A_HUMAN]	0,00	1,43	1	1	1	1		912	101,8	6,57
A6ND36	Protein FAM83G OS=Homo sapiens GN=FAM83G PE=1 SV=2 - [FA83G_HUMAN]	0,00	1,94	1	1	1	1		823	90,8	6,39
Q16676	Forkhead box protein D1 OS=Homo sapiens GN=FOXD1 PE=1 SV=1 - [FOXD1_HUMAN]	0,00	2,37	1	1	1	1		465	46,1	5,14
P16260	Graves disease carrier protein OS=Homo sapiens GN=SLC25A16 PE=1 SV=3 - [GDC_HUMAN]	0,00	7,23	1	1	1	2		332	36,2	9,85
P14866	Heterogeneous nuclear ribonucleoprotein L OS=Homo sapiens GN=HNRNPL PE=1 SV=2 - [HNRPL_HUMAN]	0,00	5,94	1	1	1	1	7,677E6	589	64,1	8,22
Q9P267	Methyl-CpG-binding domain protein 5 OS=Homo sapiens GN=MBD5 PE=1 SV=3 - [MBD5_HUMAN]	0,00	0,94	1	1	1	1	1,061E8	1494	159,8	8,98
Q86YW9	Mediator of RNA polymerase II transcription subunit 12-like protein OS=Homo sapiens GN=MED12L PE=1 SV=2 - [MD12L_HUI	0,00	0,89	1	1	1	1	5,676E6	2145	240,0	7,77
Q9NXD2	Myotubularin-related protein 10 OS=Homo sapiens GN=MTMR10 PE=1 SV=3 - [MTMRA_HUMAN]	0,00	3,99	1	1	1	1	2,167E8	777	88,2	8,53
P35579	Myosin-9 OS=Homo sapiens GN=MYH9 PE=1 SV=4 - [MYH9_HUMAN]	0,00	0,77	2	1	1	1	2,257E6	1960	226,4	5,60
Q8NEV4	Myosin-IIIa OS=Homo sapiens GN=MYO3A PE=1 SV=2 - [MYO3A_HUMAN]	0,00	1,05	1	1	1	1		1616	186,1	8,91
Q8WVE0	EEF1A lysine methyltransferase 1 OS=Homo sapiens GN=EEF1AKMT1 PE=1 SV=1 - [EFMT1_HUMAN]	0,00	5,61	1	1	1	2		214	24,5	4,55
Q6P3X8	PiggyBac transposable element-derived protein 2 OS=Homo sapiens GN=PGBD2 PE=2 SV=1 - [PGBD2_HUMAN]	0,00	4,73	1	1	1	1		592	68,0	8,54
Q15269	Periodic tryptophan protein 2 homolog OS=Homo sapiens GN=PWP2 PE=2 SV=2 - [PWP2_HUMAN]	0,00	2,07	1	1	1	1		919	102,4	6,15
P24928	DNA-directed RNA polymerase II subunit RPB1 OS=Homo sapiens GN=POLR2A PE=1 SV=2 - [RPB1_HUMAN]	0,00	0,81	1	1	1	1		1970	217,0	7,37
Q8TEQ0	Sorting nexin-29 OS=Homo sapiens GN=SNX29 PE=1 SV=3 - [SNX29_HUMAN]	0,00	3,69	1	1	1	1		813	91,2	6,21
Q6ZRS2	Helicase SRCAP OS=Homo sapiens GN=SRCAP PE=1 SV=3 - [SRCAP_HUMAN]	0,00	0,25	1	1	1	1	3,281E7	3230	343,3	5,96
Q9ULQ1	Two pore calcium channel protein 1 OS=Homo sapiens GN=TPCN1 PE=1 SV=3 - [TPC1_HUMAN]	0,00	1,84	1	1	1	1	1,189E8	816	94,1	8,27
Q8NG11	Tetraspanin-14 OS=Homo sapiens GN=TSPAN14 PE=1 SV=1 - [TSN14_HUMAN]	0,00	2,96	1	1	1	1	2,766E9	270	30,7	6,84
P42681	Tyrosine-protein kinase TXK OS=Homo sapiens GN=TXK PE=1 SV=3 - [TXK_HUMAN]	0,00	4,36	1	1	1	1		527	61,2	7,97
P15498	Proto-oncogene vav OS=Homo sapiens GN=VAV1 PE=1 SV=4 - [VAV_HUMAN]	0,00	0,83	1	1	1	1	3,805E7	845	98,3	6,62
P52747	Zinc finger protein 143 OS=Homo sapiens GN=ZNF143 PE=1 SV=2 - [ZN143_HUMAN]	0,00	2,66	1	1	1	1	2,626E6	638	68,9	6,05

Supplemental Table S5. Primer sequences used in qPCR assays

Gene	Forward primer	Reverse primer
IFNA	GGTGACAGAGACTCCCCTGA	CAGGCACAAGGGCTGTATTTCTT
IRF7	GCTGGACGTGACCATCATGTA	GGGCCGTATAGGAACGTGC
IL33	GTGACGGTGTTGATGGTAAGAT	AGCTCCACAGAGTGTTCCTTG
GAPDH	CATGTTCCAATATGATTCCACC	GATGGGATTTCCATTGATGAC
HPRT1	GACCAGTCAACAGGGGACAT	CTTGCGACCTTGACCATCTT

Full unedited blot for Figure 3B

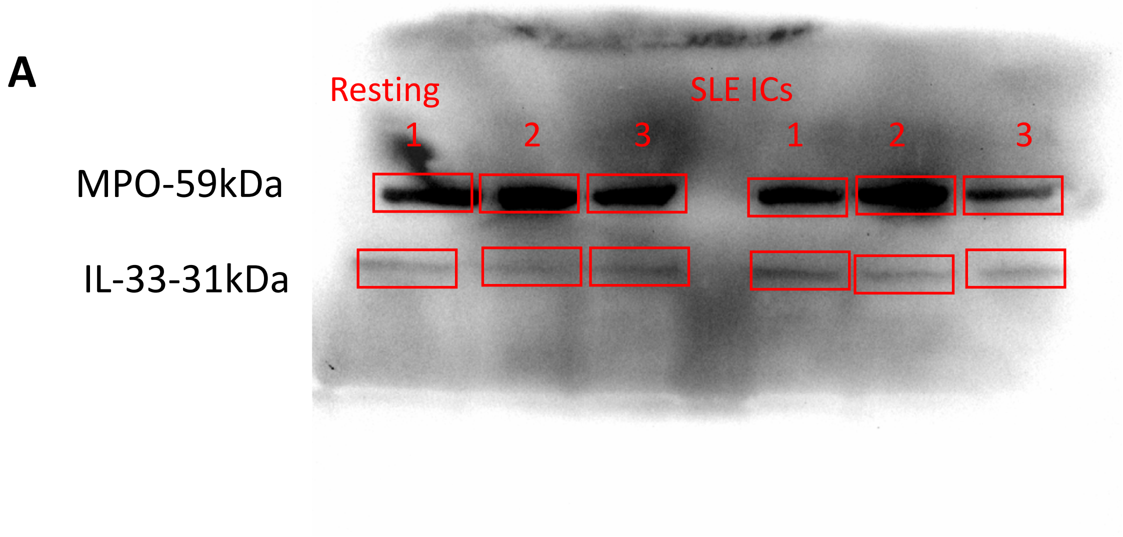

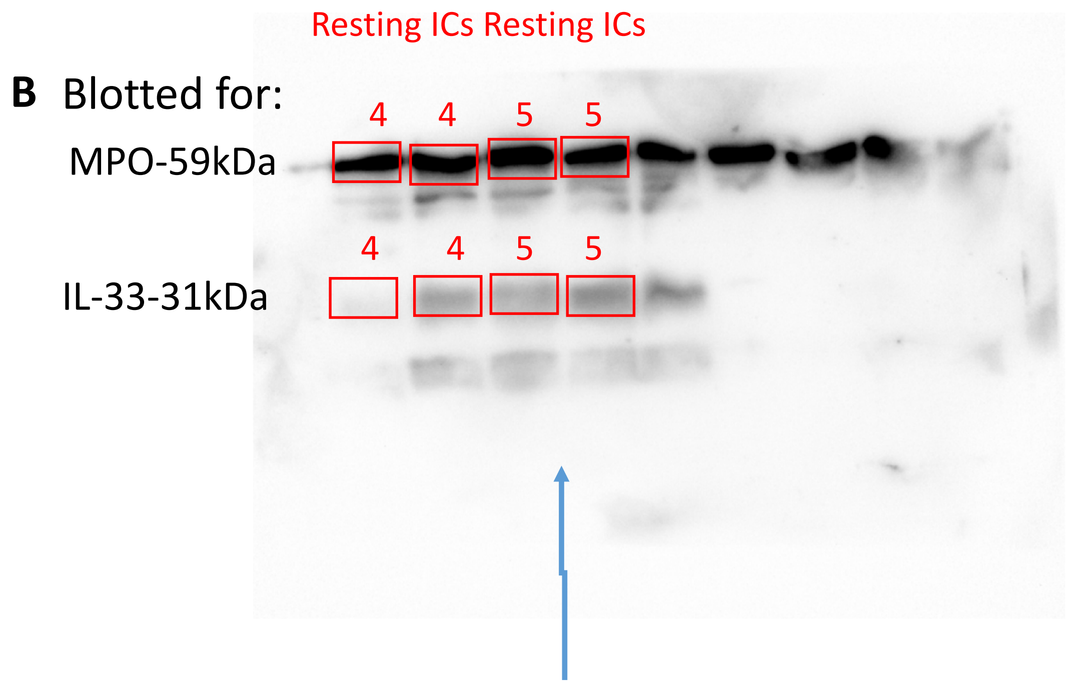

*Used only for IL-33 relative quantification, not shown in the manuscript

Full unedited blot for Figure 3D

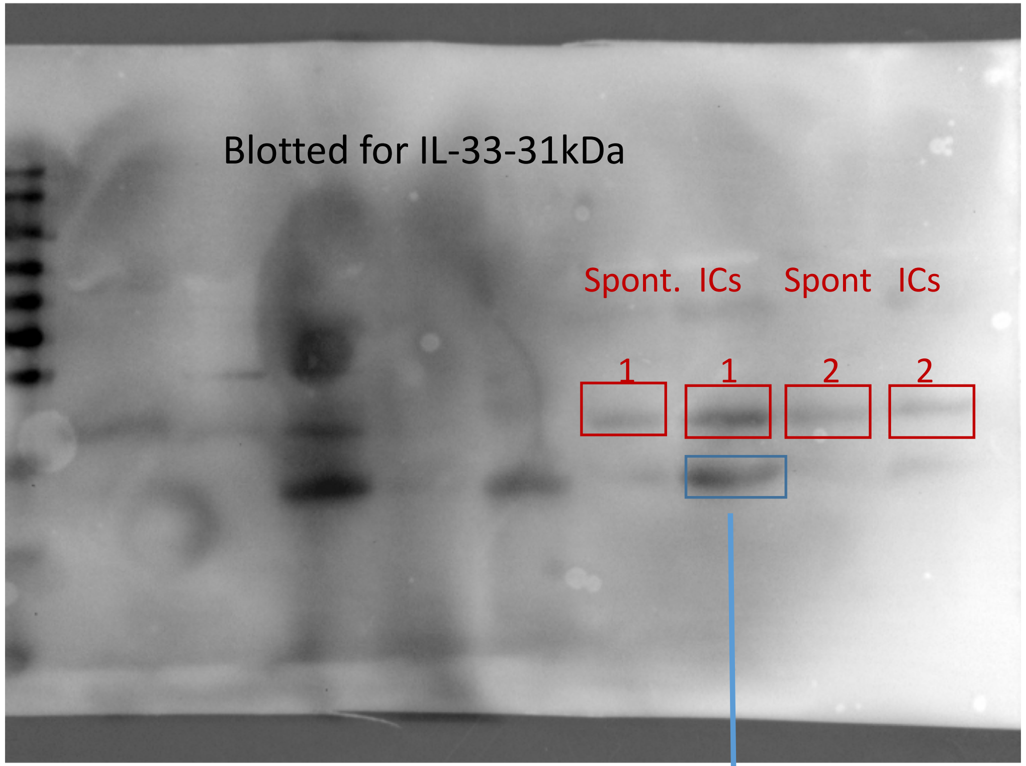

Light chain of IgG Ab (26kDa)-non specific signal
Detected only in IC-mediated NET extracts

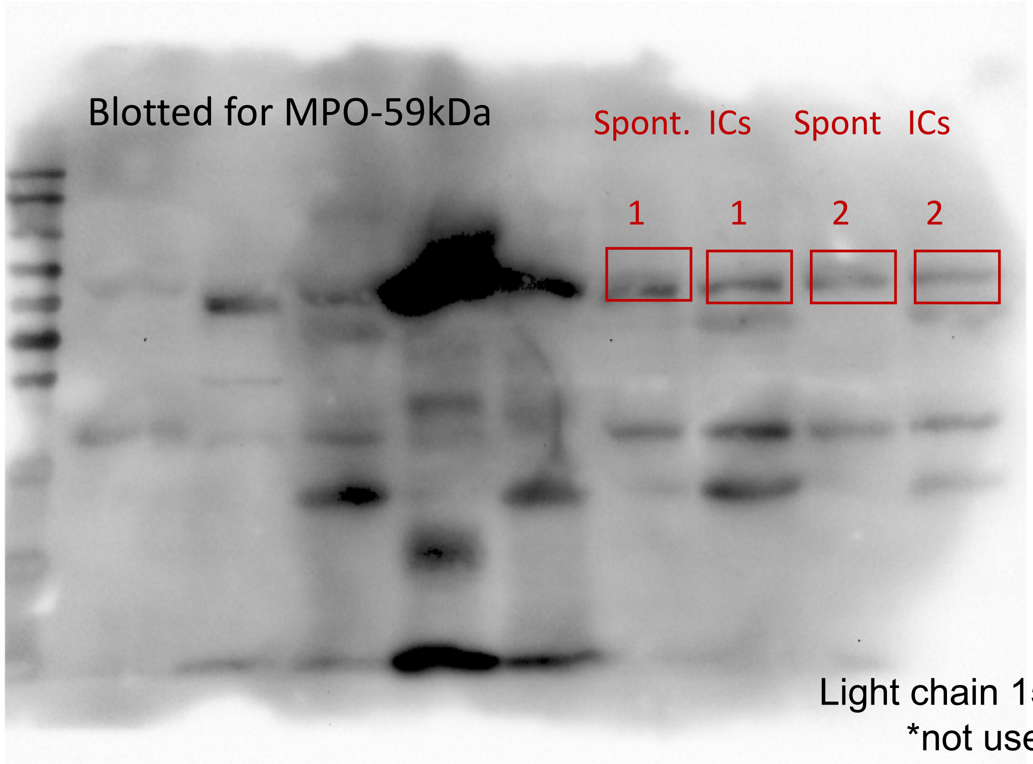

MPO
Light chain 15kDa
*not used for
quantification

Full unedited blot for Figure 7A

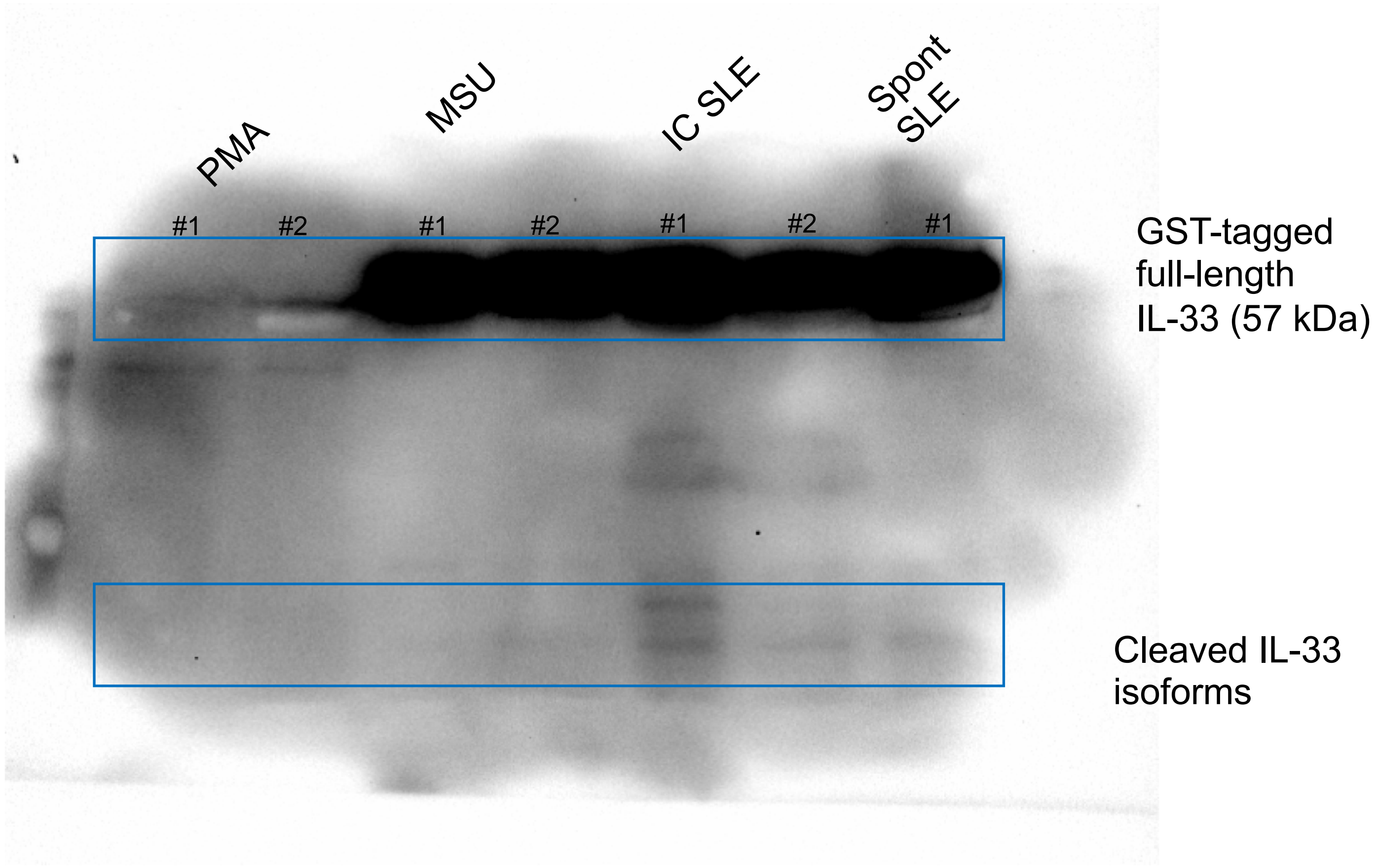

Full unedited blot for Figure 7B

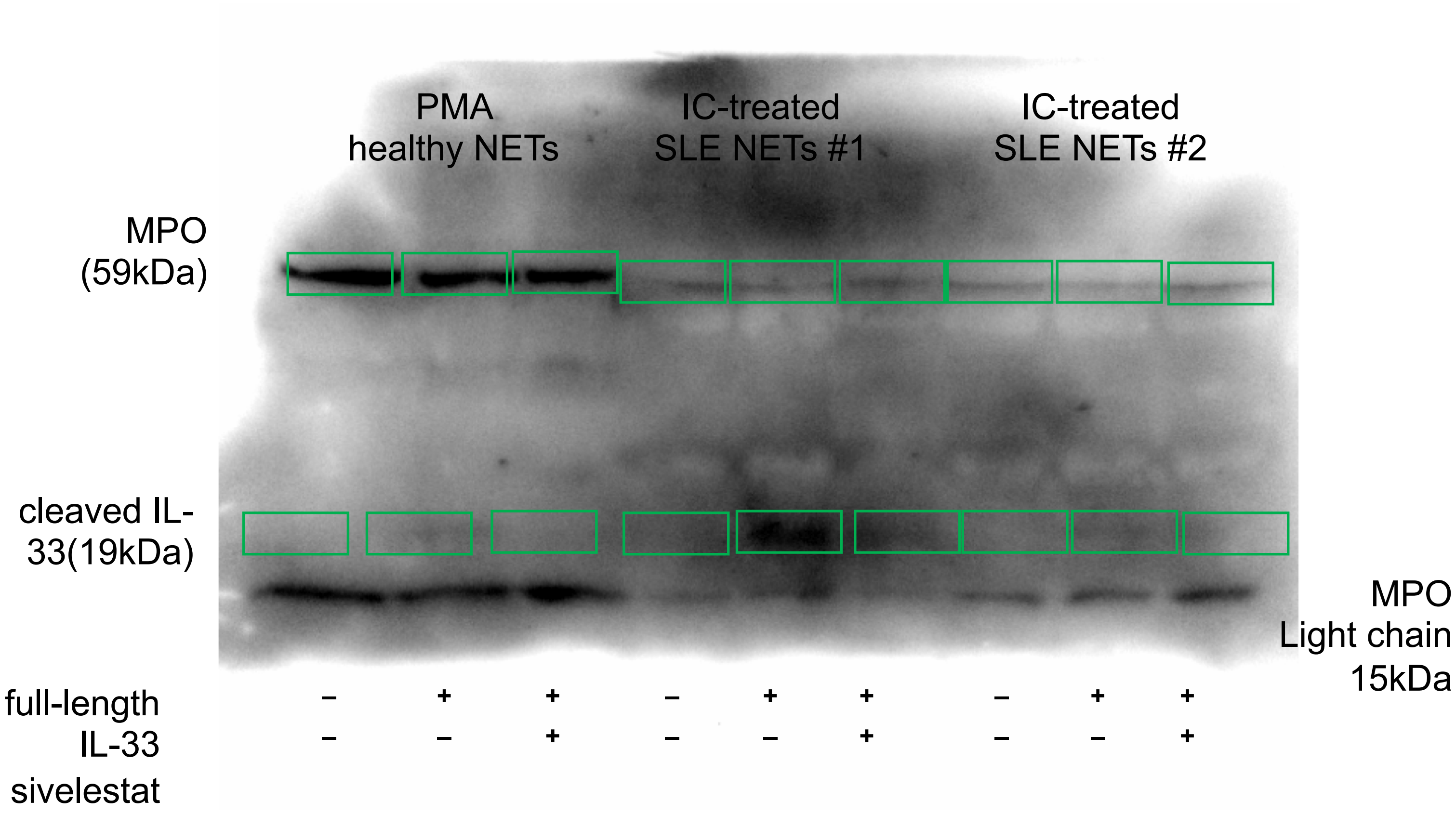

Full unedited blot for Figure S4A

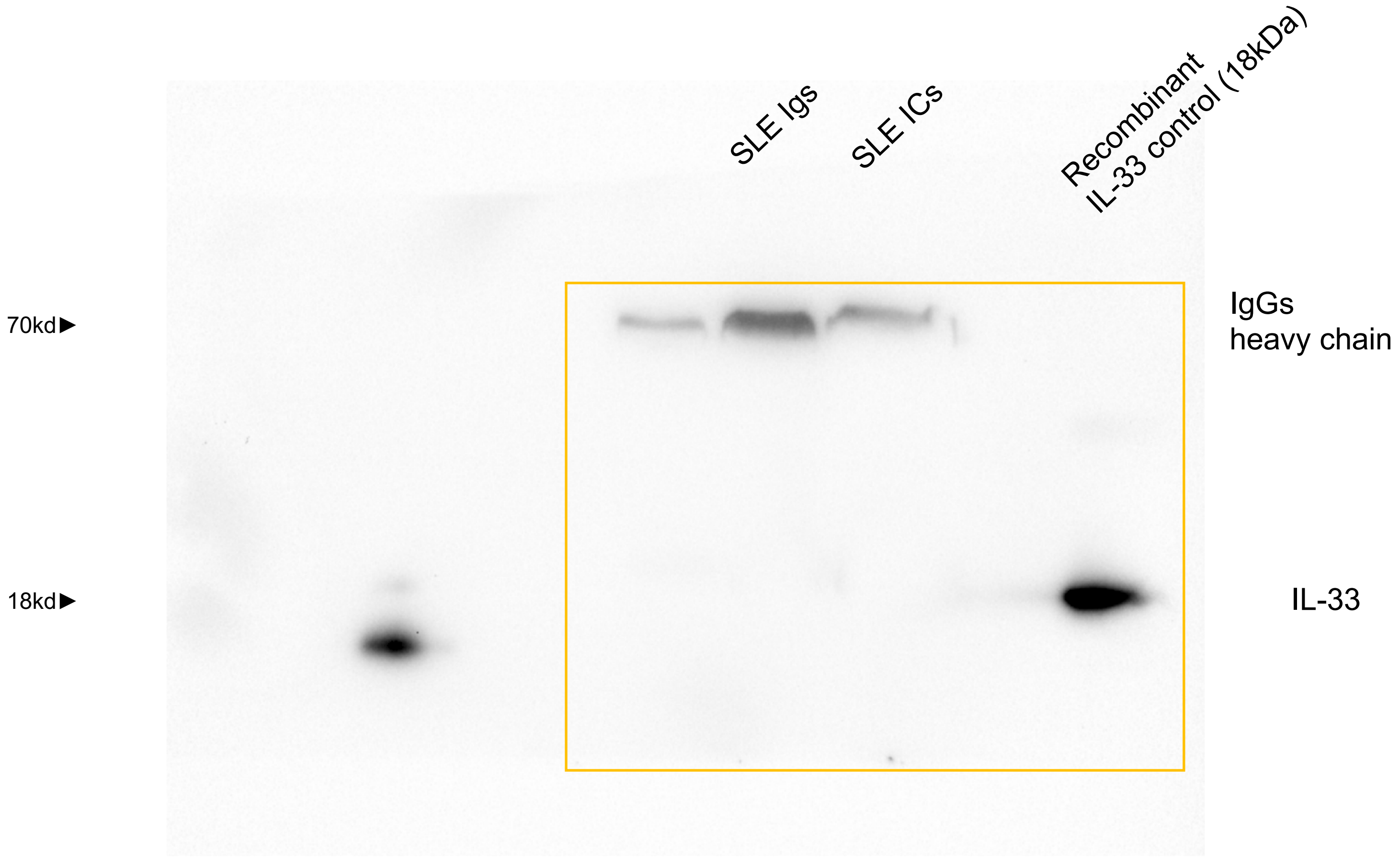

Full unedited blot for Figure S6C

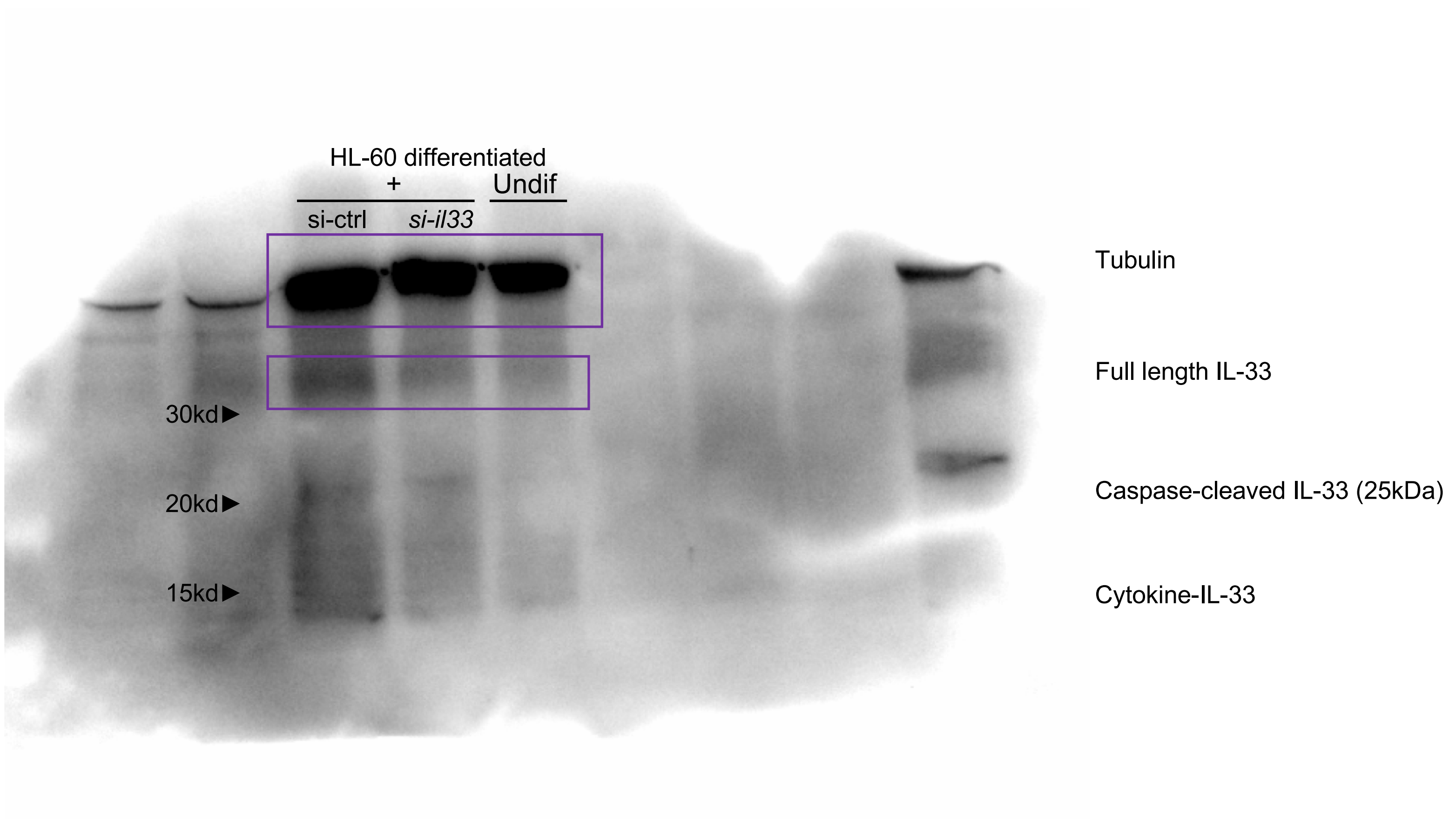

Full unedited blot for Figure S7D?

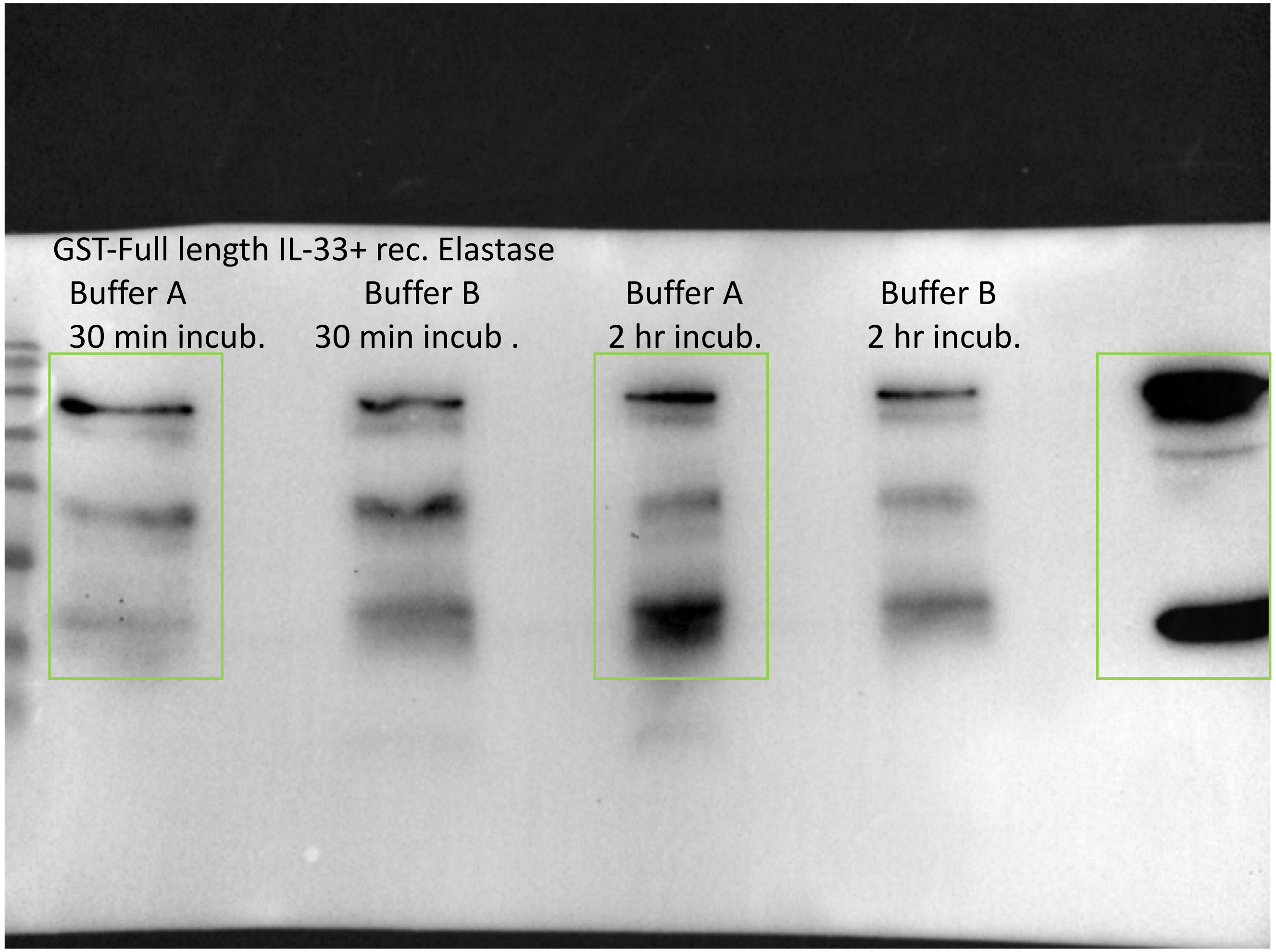

Controls

GST-tagged full-length IL-33 (57 kDa)

Recombinant cytokine IL-33 (18kDa)
